# scTour: a deep learning architecture for robust inference and accurate prediction of cellular dynamics

**DOI:** 10.1186/s13059-023-02988-9

**Published:** 2023-06-23

**Authors:** Qian Li

**Affiliations:** Department of Pathology, University of Cambridge, Cambridge, UK

**Keywords:** Deep learning, Cellular dynamics inference and prediction, Developmental pseudotime, Vector field, Latent space

## Abstract

Despite the continued efforts, a batch-insensitive tool that can both infer and predict the developmental dynamics using single-cell genomics is lacking. Here, I present scTour, a novel deep learning architecture to perform robust inference and accurate prediction of cellular dynamics with minimal influence from batch effects. For inference, scTour simultaneously estimates the developmental pseudotime, delineates the vector field, and maps the transcriptomic latent space under a single, integrated framework. For prediction, scTour precisely reconstructs the underlying dynamics of unseen cellular states or a new independent dataset. scTour’s functionalities are demonstrated in a variety of biological processes from 19 datasets.

**Supplementary Information:**

The online version contains supplementary material available at 10.1186/s13059-023-02988-9.

## Background

Among the challenges that decoding developmental processes at single-cell resolution using single-cell RNA sequencing (scRNA-seq) poses, a unique difficulty is that scRNA-seq can only capture static snapshots of cells. In addition, experimental assays such as lineage tracing and metabolic labelling are inaccessible to many biological systems particularly those involving human tissues [1–5]. Many computational tools have been developed to analyze these dynamic processes, the most prevalent of which are pseudotime-based ordering of cells along their trajectory and RNA velocity-based directing of future cell states [6–10]. Despite the wide usefulness of these tools, they have several limitations which restrict their scope: (1) the majority of tools for pseudotime estimation require the users to explicitly designate the starting cells, meaning that they are limited to well-studied biological processes. (2) The existing RNA velocity-based tools are largely focused on the modelling of transcriptional kinetics. This requires either the extraction of spliced and unspliced mRNAs within cells, a rate-limiting step especially for large-scale datasets, or information from metabolic labelling which is often not possible especially when applied to human tissues [9]. This could also lead to inaccurate inference due to the assumption of constant kinetic rates and the noisy approximation of nascent transcripts by intronic reads [11]. Moreover, they are not readily adaptable to use cases beyond scRNA-seq. (3) Current algorithms are affected by batch effects to varying degrees, often involving the use of external batch correction tools to derive a batch-free embedding for velocity visualization or pseudotime inference. This is particularly difficult for time-course experiments. (4) The prediction functionality is lacking or quite limited in the current methods. Neither the pseudotime nor the vector field can be made predictable for unseen data. Although two recent studies did use the vector field to predict the transcriptomic space forward or backward given an initial cell state [9, 12], predicting unseen cellular states is challenging for these tools. All these issues restrict the current methods to the data they have modelled and hinder the transfer and generalization to new datasets.

Here I introduce scTour, an innovative deep learning-based architecture that, in addition to overcoming the limitations detailed above, achieves multifaceted dissection of a variety of biological processes under a single model in an unsupervised manner. scTour simultaneously infers the developmental pseudotime, transcriptomic vector field, and latent space of cells, with all these inferences largely unaffected by batch effects inherent in the datasets. Another advantage is that the pseudotime estimation does not need input of a starting cell, and the vector field inference does not rely on the discrimination between spliced and unspliced mRNAs, rendering scTour applicable to other genomic data. Importantly, the inference of a low-dimensional latent space which combines the intrinsic transcriptome and extrinsic time information provides richer information for reconstructing a finer cell trajectory. Its insensitivity to batch effects also allows for unbiased integration of different datasets. Uniquely in scTour, the resulting model can be further employed to predict the transcriptomic properties and dynamics of unseen cellular states and even to predict the characteristics of a different dataset new to the model. These together make scTour a generative and powerful method for single-cell developmental data analysis. To demonstrate the superiority of scTour, I have applied it to a wide variety of dynamic biological processes including neurogenesis, pancreatic endocrinogenesis, skeletal muscle, thymic epithelial cell and embryonic development, hematopoiesis, and brain vasculature zonation (scRNA-seq), as well as reprogramming (single-nucleus RNA sequencing (snRNA-seq)) and human fetal retinal development (single-cell ATAC-sequencing (scATAC-seq)). In all of these systems, the accuracy and effectiveness of scTour in recapitulating the underlying cellular dynamics was validated. scTour is available as an open-source software at https://github.com/LiQian-XC/sctour.

## Results

### The scTour architecture

**Fig. 1:**
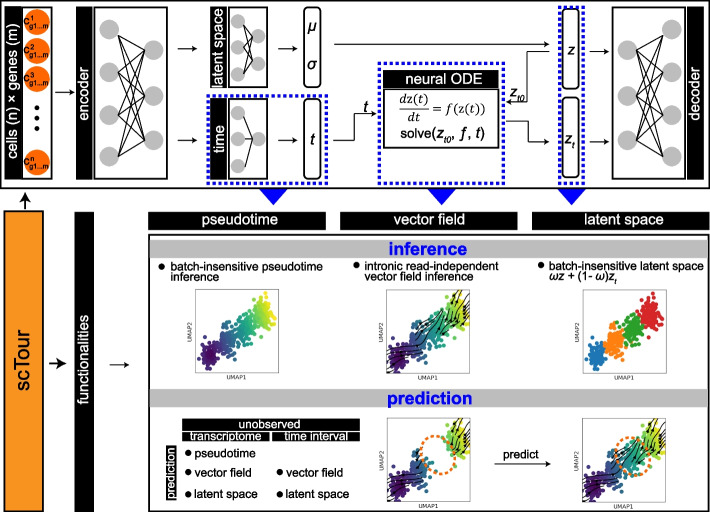
scTour framework. With a gene expression matrix as input, two encoder networks are used to both generate the distribution parameters of the approximate posterior (latent space, $z$) and assign a time point to each cell (time, $t$). The sample from the posterior at the initial state (${z}_{{t}_{0}}$) along with the times (${t}_{0}, {t}_{1}, {t}_{2}, \dots , {t}_{n}$) of cells are input into a neural ODE to yield another series of latent representations ${z}_{t}$. A decoder network then reconstructs the input using the latent $z$ and ${z}_{t}$. This model can be used to infer the developmental pseudotime, transcriptomic vector field, and latent representations of cells in an unsupervised manner, as well as to predict the cellular dynamics of unobserved transcriptomes or time intervals

scTour is a new deep learning architecture that builds on the framework of variational autoencoder (VAE) [13] and neural ordinary differential equation (ODE) [14] accompanied by critical innovations tailored to the analysis of dynamic processes using single-cell genomic data (Fig. 1). Specifically, given a gene expression matrix, scTour leverages a neural network to assign a time point to each cell in parallel to the neural network for latent variable parameterization. The resulting time information allows scTour to spot the initial latent state ${z}_{{t}_{0}}$, which is further combined with the estimated time of each cell to solve an ODE, with the derivative of latent states with respect to time defined by another neural network (Fig. 1). The ODE solver yields another series of latent representations, together with the ones from the variational inference, to serve as the input for reconstructing the transcriptomes in a weighted manner (see “Methods”).

Compared to the latent ODE model proposed in the original neural ODE publication [14], scTour delivers three major innovations. Firstly, scTour introduces a neural network for inferring the developmental time of a given cell based on its transcriptome. This operation enables the model to bypass the dependence on the prior knowledge of the cell timeline, and endows scTour with the ability to suit any data beyond the timestamped ones. Secondly, different from the original model, which adopts a recurrent neural network (RNN) as the recognition net to derive the latent state only at time ${t}_{0}$, scTour employs the typical encoder network to infer the latent states covering all observations. These are then used to reconstruct the transcriptomic space concurrently with the ones from the ODE solver. Such an operation preserves the intrinsic transcriptomic structure of cells and proves a superior strategy in reconstructing the trajectory. Thirdly, scTour utilizes the standard mini-batch training which is less straightforward in the original latent ODE model [14]. With this optimization, scTour’s performance is again improved, being highly efficient and scalable to large-scale datasets.

As a result, scTour provides two main functionalities in deciphering cellular dynamics in a batch-insensitive manner: inference and prediction (Fig. 1). For inference, the time neural network in scTour allows estimates of cell-level pseudotime along the trajectory. The learned differential equation (i.e., the latent state’s derivative with respect to time) by another neural network provides an alternative way of inferring the transcriptomic vector field. This eliminates the time-consuming step of distinguishing spliced from unspliced mRNAs used in RNA velocity-based tools and thus can be extended to other genomic data. The variational inference and ODE solver yield a combined latent representation which contains richer information for reconstructions of developmental trajectories, cellular stratifications, and data integrations. For prediction, given an unobserved cellular state or a new dataset agnostic to the model, the time neural network trained in scTour can predict its developmental pseudotime; the learned differential equation can infer its transcriptomic vector field; the latent space is likewise predictable. Notably, the latent space of an unseen cellular state can also be reconstructed by providing the model with its expected developmental time. All these are novel and powerful features adding to the existing trajectory inference tools.

### scTour’s inference captures the underlying developmental dynamics

**Fig. 2:**
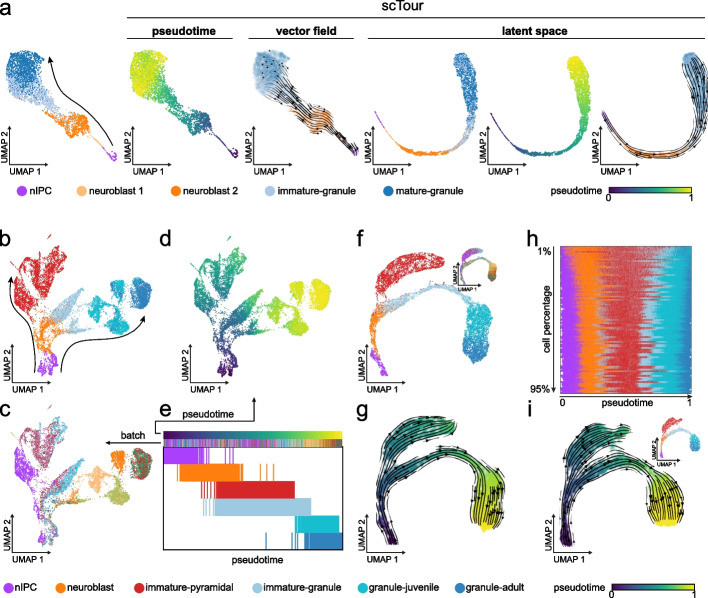
scTour robustly captures the cellular dynamics during dentate gyrus neurogenesis. **a** UMAP visualizations of the cell types from the granule cell lineage (4007 cells) [15], and the developmental pseudotime, transcriptomic vector field, and latent representations inferred by scTour. Leftmost panel shows the PCA space-based UMAP with the arrow indicating the differentiation from nIPCs to mature granule cells. **b** PCA space-based UMAP embedding showing the cell types (colors, 15,174 cells) [15] along the pyramidal and granule cell lineages (arrows). **c** As in **b**, but colored by sample batches. **d** As in **b**, but colored by the developmental pseudotime derived from the scTour model. **e** Developmental ordering of cells by the pseudotime inferred from scTour. Cells are colored from top to bottom by pseudotime, sample batches, and cell types. **f** UMAP visualizations of the latent representations learned from scTour, with colors denoting the cell types and sample batches (inset). **g** Streamline visualization of the transcriptomic vector field from scTour on the same embedding as in **f**, with cells color-coded by the inferred pseudotime. **h** Developmental ordering of cells by the pseudotime estimated from scTour models trained using a range of cell subsets (1 to 95% of total cells from top to bottom). Cells are colored by cell types. **i** UMAP visualizations of the latent representations, developmental pseudotime (colors), and transcriptomic vector field (streamlines) learned from the scTour model trained based on 20% of total cells. The inset shows the same plot but color-coded by cell types

I first evaluated scTour using a scRNA-seq dataset from the mouse dentate gyrus during postnatal development. The focus here was on the granule cell lineage which undergoes sequential transcriptomic changes from neuronal intermediate progenitor cells (nIPCs), neuroblasts, immature granule cells, to mature granule cells [15] (4007 cells, Fig. 2a). Following the scTour model training (see “Methods”), the developmental pseudotime, transcriptomic vector field, and low-dimensional latent space (set as five dimensions) of cells were derived (Fig. 2a). The estimated pseudotime clearly recapitulated the developmental process of granule cells, with the transcriptional continuum from nIPCs to mature granule cells captured. Similarly, analysis of the vector field delineated the expected directional flow along the differentiation path when visualized on the uniform manifold approximation and projection (UMAP) embedding (Fig. 2a). Of note, it performed better than the intronic read-based velocity estimate which failed to capture the immature to mature granule cell transition (Additional file 1: Fig. S1). The latent space computed by scTour through incorporating both the intrinsic transcriptome and extrinsic pseudotime information not only reflected the transcriptomic differences among cell types, but also charted a finer continuous trajectory underlying the developmental process of granule cells when compared to that constructed from the PCA space (Fig. 2a).

### scTour’s inference is insensitive to batch effects and cell subsampling

The advantages of applying scTour to a linear and continuous developmental process are clear. To further test its capability in dealing with more complex processes, I next applied scTour to another scRNA-seq dataset from the developing mouse dentate gyrus which collected some extra immature pyramidal neurons from the hippocampus proper [15]. I focused on the granule cell lineage along with the immature pyramidal neurons; in the original study, it was suggested that they shared a differentiation trajectory (15,174 cells, Fig. 2b). This dataset presented substantial batch effects from different samples that segregated cells significantly within the same cell type (Fig. 2c). Nevertheless, scTour successfully recapitulated the two differentiation branches with a minimal impact from the sample batches due to the continuous-in-time transformations of the latent states by the ODE solver (Fig. 2d–g). Specifically, the estimated pseudotime was in line with the differentiation courses, depicting the gradual progression from nIPCs to both granule cells and pyramidal neurons (Fig. 2d,e). The inferred latent space was also largely batch free and constructed an improved cell differentiation trajectory (Fig. 2f). Projecting the vector field onto this trajectory further, scTour again corroborated the shared trajectory between granule and pyramidal cell lineages, with the immature parts of both cell populations branching out from the neuroblasts (Fig. 2g). This feature of scTour is of critical importance to cross-platform or cross-study data integrations and comparisons because it is not conditioned on batch corrections and thus alleviates the risk of overcorrection when batch confounders and biological signals are entangled (such as two organs from two individuals respectively). When further incorporating more cell lineages including those leading to astrocytes, oligodendrocytes, and pyramidal neurons from hippocampal subfields into this analysis, scTour again captured the branching events albeit with an undetermined root state between immature astrocytes and radial glia possibly due to their shared glia-like traits (Additional file 1: Fig. S2).

Given scTour’s design of model-based prediction and implementation of mini-batch training, it was possible that a scTour model could be trained from a subset of data and the resulting model could be used to derive the characteristics of the entire dataset. To test this possibility, I trained scTour models on the same dataset but used a series of subsets ranging from 1 to 95% of all cells. The results highlighted the robustness of scTour, as both the granule and pyramidal cell lineages already manifested when the model was trained from as small as 1% of all cells (Additional file 1: Fig. S3a-c). Across the subsampling span from 1 to 95%, the inferred full spectrum of cellular dynamics converged quickly (Fig. 2h and Additional file 1: Fig. S3d). To illustrate this, it was clear that the pseudotime, vector field, and latent space learned from 20% of data successfully reconstructed the full granule and pyramidal cell differentiation paths (Fig. 2i). For all these analyses, since the scTour model was trained with a small subset of cells (20%), it took 12 min for the model training using CPU only and 1 s to propagate to full data inference (15,174 cells). All these endow scTour with remarkable efficiency and scalability when dealing with large-scale datasets.

Taken together, scTour can characterize dynamic processes comprehensively, robustly, and efficiently, allowing for its application to diverse datasets from different biological processes, systems, species, and experimental platforms. These include, but are not limited to, mouse embryonic organoids [16] (30,496 cells, Additional file 1: Fig. S4), human thymic epithelial cell development [17] (14,217 cells, Additional file 1: Fig. S5), human embryonic development [18, 19] (1195 cells, Additional file 1: Fig. S6; 90 cells, Additional file 1: Fig. S7), induced pluripotent stem cell (iPSC) reprogramming [20, 21] (251,203 cells, Additional file 1: Fig. S8; 36,597 nuclei, Additional file 1: Fig. S9), hematopoiesis [9] (1947 cells, Additional file 1: Fig. S10), and brain vasculature zonation [22] (3105 cells, Additional file 1: Fig. S11). All these analyses demonstrated the efficiency and accuracy of scTour’s inference. A particular advantage of scTour is that the transcriptomic vector field can be directly obtained from single-nucleus data to elucidate the reprogramming process (Additional file 1: Fig. S9). This is challenging for RNA velocity-based tools due to the disruption of the balance between spliced and unspliced transcripts during the nucleus isolation [11]. Another striking example was the delineation of a dataset focussed on hematopoiesis where the underlying cell trajectory was not captured by the spliced RNA velocity but only by the total RNA velocity from metabolic labelling [9]. With scTour, this process was easily depicted with no dependence on extra information or experimental assays (Additional file 1: Fig. S10).

### scTour’s prediction reconstructs the dynamics of unseen cellular states

**Fig. 3:**
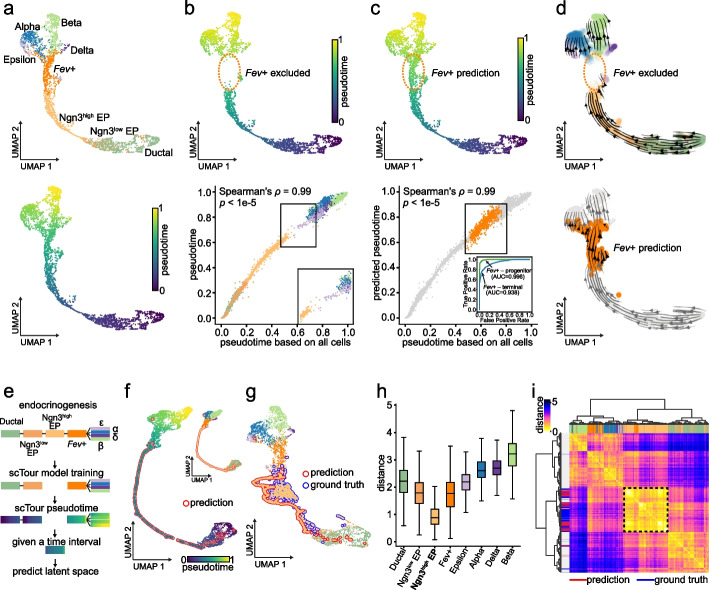
scTour reconstructs the cellular dynamics of unseen cellular states in pancreatic endocrinogenesis. **a** UMAP visualizations of the latent space from scTour based on 3696 cells from [23], colored by cell types (top), and pseudotime (bottom). **b** Top: UMAP representation showing the pseudotime from the model trained with the *Fev* + cells excluded. Bottom: scatter plot comparing the pseudotime estimates (*y*-axis) with those inferred from the full dataset (*x*-axis), with Spearman’s *ρ* and *p*-value between them shown. **c** Top: UMAP representation displaying the predicted pseudotime for the held-out *Fev* + cells (dotted circle). Bottom: scatter plot comparing the prediction (*y*-axis) with the ground truth (*x*-axis) highlighted in orange, with Spearman’s *ρ* and *p*-value between pseudotime in *x* and *y* axes shown. Bottom inset: ROC curve for the binary classifications of endocrine progenitors versus *Fev* + cells (green) and *Fev* + versus terminal cells (blue) based on the predicted (*Fev* + cells) and inferred (remaining cells) pseudotime. **d** Streamline visualizations of the vector field from the *Fev* + -excluded model (top), and the prediction for these held-out cells (bottom). **e** Schematic depicting the model training with the Ngn3^high^ EPs excluded, followed by prediction of their latent space given their expected developmental time. **f** UMAP visualization based on the reconstructed latent representations for the held-out Ngn3^high^ EPs (red outline) and those inferred from training cells, colored by pseudotime and cell identities (inset). **g** As with **f**, but with true Ngn3^high^ EPs (blue outline) incorporated. **h** Box plot displaying the Euclidean distances between the reconstructed latent representations for Ngn3^high^ EPs and those from each cellular state, with the medians, interquantile ranges, 5th, 95th percentiles indicated by center lines, hinges, and whiskers, respectively. **i** Unsupervised hierarchical clustering of the reconstructed Ngn3^high^ EPs and all the other cells based on their Euclidean distances in the latent space. Colors mark the cell types, and the reconstructed (red), true (blue) Ngn3^high^ EPs, and remaining cells (grey)

Given the predictive functionality built in scTour, I next assessed its ability to predict the characteristics of unseen cellular states (i.e., cellular states new to the model). I therefore applied scTour to a scRNA-seq dataset from the development of endocrine compartment of the mouse pancreas, as previously described in the scVelo publication [8, 23] (3696 cells). The mouse pancreatic endocrinogenesis starts from the endocrine progenitors (EPs), goes through the intermediate stage (*Fev* + endocrine cells), and finally commits to four major fates: α-cells, β-cells, δ-cells, and ε-cells. I started by training the scTour model using all the cellular states involved in this process. Here I compared the derived developmental pseudotime with scVelo’s latent time. This was because the latter was shown to delineate this process more accurately than diffusion pseudotime as it captured the earlier emergence of α-cells relative to β-cells [8]. This comparison highlighted the usefulness of scTour’s pseudotime in not only resolving the ordering of α- and β-cells, but also identifying the continuous progression from *Fev* + endocrine cells to terminal fates which was not revealed by scVelo’s latent time (Fig. 3a and Additional file 1: Fig. S12a,b). I further compared the inferred vector field with the RNA velocity estimated by scVelo and κ-velo which previously demonstrated good performance for this dataset [8, 24]. With regard to the entire differentiation course of endocrinogenesis, scTour and scVelo showed an advantage over κ-velo which only illustrated a partial view of this process when no prior knowledge was provided (Additional file 1: Fig. S13a). Further focusing on the cycling cells, scVelo captured both the S to G_2_M transition and the exit of the cell cycle while scTour captured the partial S-G_2_M transition and full cell cycle exit (Additional file 1: Fig. S13b, see “Discussion”). Neither of these two processes were properly delineated by κ-velo (Additional file 1: Fig. S13b).

Next, I excluded one cellular state, the intermediate *Fev* + endocrine cells, and trained a scTour model on the remaining cells. The aim was to test (1) whether scTour can infer the cellular dynamics of a discontinued process; and (2) whether the resulting model can be used to predict the properties of the held-out cellular state. This analysis demonstrated that scTour can recapitulate the discontinuous differentiation course, assigning near-identical pseudotime as compared to that from the analysis of the entire dataset (Fig. 3b), as well as presenting a time gap between EPs and the four terminal states as expected (Fig. 3b). By contrast, scVelo’s latent time was unable to delineate this discontinuous process in full as it failed to disentangle the continuum of early progenitor cells and to recognize the intermediate transitional process by erroneously connecting EPs with terminal states (Additional file 1: Fig. S12c,d).

On the basis of the model trained above, scTour successfully predicted pseudotime of the unseen cellular state—in this case the *Fev* + endocrine cells—filling in the time gap and thus bridging the EPs and terminal cells (Fig. 3c). In parallel, the predicted transcriptomic vector field for this cell type correctly orientated those cells towards terminal fates (Fig. 3d). Likewise, scTour-derived latent space preserved the expected gap corresponding to the held-out *Fev* + endocrine cells and the predicted latent representation reconstructed the full trajectory of endocrinogenesis by placing *Fev* + cells properly along the differentiation path (Additional file 1: Fig. S14). In addition to the intermediate cellular states, scTour was capable of reconstructing the dynamics of unobserved starting or terminal states (Additional file 1: Fig. S14). Taken together, scTour can perform precise out-of-distribution predictions beyond the inference.

### scTour reconstructs the transcriptomic space at unobserved time intervals

During development, some intermediate cell states are often transient or present in small quantities. Reconstructing transcriptomic signatures of these cells will be useful when there is limited coverage of particular cell types. scTour allows inference of the transcriptomic characteristics of uncaptured cellular states based merely on their expected developmental time, achieved by integrating the ODE in a stepwise manner and taking into account the *k*-nearest neighbors in the time space when inferring the latent representation at an unobserved time point (see “Methods”). To test this functionality, a scTour model was trained using the same dataset of pancreatic endocrinogenesis described above but with Ngn3^high^ EPs located between Ngn3^low^ EPs and intermediate *Fev* + endocrine cells excluded. After training, scTour correctly assigned the developmental pseudotime to each cell, leaving an anticipated time gap corresponding to the missing Ngn3^high^ EP population (Fig. 3e).

Next, when this time interval was provided as the only input to the trained scTour model, the transcriptomic latent space corresponding to this time span was reconstructed and shown to locate at the expected position between Ngn3^low^ EPs and *Fev* + endocrine cells, forming a complete continuous trajectory together with other cells (Fig. 3f). Of note, this was a rather long-range prediction covering an entire cellular state. When further projecting all the cells onto the same UMAP embedding, the reconstructed and ground-truth Ngn3^high^ EPs were placed together, indicating their transcriptomic similarity (Fig. 3g). This was reinforced by their shortest distance through the comparison with each cellular state in the latent space, revealing the expected trend of transcriptomic differences following the differentiation progression (Fig. 3h). More specifically, unsupervised clustering using the derived distances rebuilt a tree which not only revealed the developmental relations among cell types but also grouped the predicted and true Ngn3^high^ EPs into a single branch (Fig. 3i). All these results illustrated the accuracy of scTour in reconstructing the transcriptomic space at unobserved intermediate time intervals. Besides, scTour can be leveraged to recover the unobserved starting and terminal states (Additional file 1: Fig. S15). Altogether, scTour allows simulation of cellular states that have not been captured during a scRNA-seq experiment.

### scTour can perform cross-platform, -system, -species predictions

**Fig. 4:**
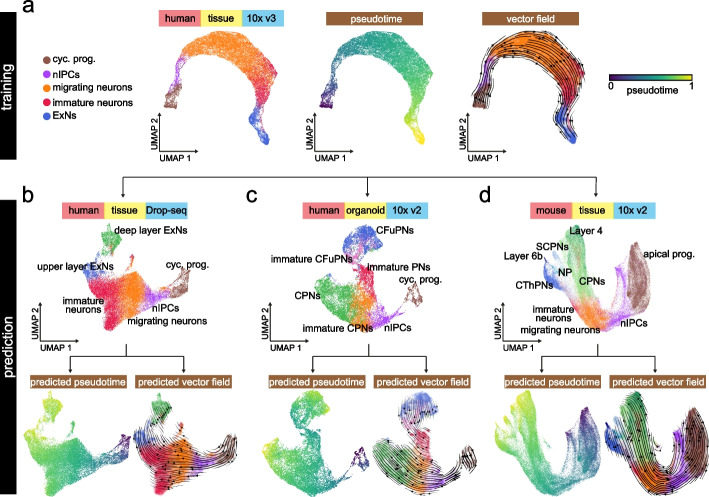
Cross-platform, -system, -species predictions of cellular dynamics during excitatory neuron development by scTour. **a** UMAP visualizations of the latent space (left, colored by cell types), developmental pseudotime (middle), and transcriptomic vector field (right) estimated by the scTour model trained using 60% of the 36,318 cells from the developing human cortex (10x Genomics) [25]. **b–d** Top: UMAP visualizations of the cell types from another developing human cortex dataset (Drop-seq, 27,855 cells) [26] (**b**), a human brain organoid dataset (10x Genomics, 16,032 cells) [27] (**c**), and a developing mouse cortex dataset (10x Genomics, 73,649 cells) [28] (**d**). Bottom: the predicted pseudotime (left panels) as well as transcriptomic vector fields (right panels) for these three test datasets by the scTour model from **a**. cyc. prog., cycling progenitors; nIPCs, neuronal intermediate progenitor cells; ExNs, excitatory neurons; PNs, projection neurons; CPNs, callosal projection neurons; CFuPNs, corticofugal projection neurons; CThPNs, corticothalamic projection neurons; NP, near projecting; SCPNs, subcerebral projection neurons; apical prog., apical progenitors

Given the capability of scTour to characterize unseen cellular states, I next tested in a broader context the ability of scTour to predict the cellular dynamics of datasets that differ in many aspects from the one used to train the model. Here I selected the process of cortical excitatory neuron differentiation which has been well described in different species and biological systems using single-cell genomics [25–28]. Specifically, I trained the scTour model using a scRNA-seq dataset profiling the developing human cortex with the 3′ Kit v3 of 10x Genomics [25]. I analyzed the same set of cells used in the original study for reconstruction of the excitatory neuron trajectory (36,318 cells). Before the model training, the excitatory neurons were relabelled according to their degree of maturity along the differentiation course (Additional file 1: Fig. S16a). The resulting scTour model, as expected, charted the cell differentiation trajectory from cycling progenitors, nIPCs, migrating neurons, immature to mature excitatory neurons, as evidenced by the developmental pseudotime, transcriptomic vector field, and latent space robustly inferred, regardless of the substantial batch effects present in this dataset (Fig. 4a and Additional file 1: Fig. S16b).

Given this model, I next assessed its performance in cross-data predictions by testing three additional datasets covering different experimental platforms, biological systems, and species: (1) Drop-seq-based measuring of the developing human cortex [26] (27,855 cells); (2) an in vitro organoid system modelling the human cerebral cortex [27] (10x Genomics 3′ Kit v2, 16,032 cells); (3) developing cortex from a different species, mouse [28] (10x Genomics 3′ Kit v2, 73,649 cells). Despite large discrepancies between these three test datasets and the one used for training, scTour successfully reconstructed the cell trajectories mirroring excitatory neuron differentiation for all three datasets. This was shown by the precisely predicted pseudotime, vector field, and latent space without any prior corrections of batch effects present across all datasets (Fig. 4b–d and Additional file 1: Fig. S16c-e). Altogether, the dynamic properties of a new dataset can be efficiently decoded by scTour with a negligible time cost in prediction. It is thus a new useful tool for cross-data integrations and comparisons.

### Comparison of scTour with existing algorithms

**Fig. 5:**
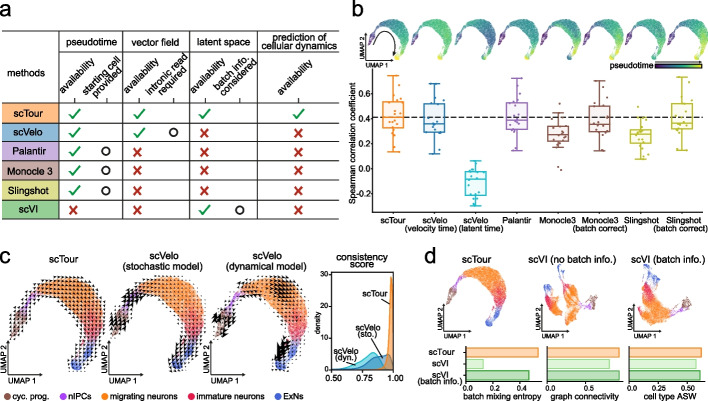
Benchmarking scTour against existing methods. **a** Table summarizing the functionalities and features of scTour versus other methods. **b** Top panels: UMAP visualizations of the pseudotime estimated by different algorithms along the excitatory neuron development (36,318 cells [25]). Bottom panel: Box plot showing the Spearman correlation coefficients calculated between the pseudotime estimates and the expression profiles of 20 well-established marker genes along the excitatory neuron developmental trajectory (see “Methods”). The horizontal dotted line denotes the median value from scTour. **c** UMAP visualizations of the vector fields from scTour (left), scVelo’s stochastic (middle) and dynamical (right) models, with colors indicating different cell types. The rightmost panel illustrates the distributions of the consistency scores calculated between each cell and its neighboring cells based on the vector fields derived from each method. cyc. prog., cycling progenitors; nIPCs, neuronal intermediate progenitor cells; ExNs, excitatory neurons. **d** Top panels: UMAP visualizations based on the latent space from scTour (left), scVI without batch correction (middle), and scVI with batch information incorporated during model training (right). Cells are color-coded by cell types as in **c**. Bottom panels: metrics quantifying the degree of batch correction (batch mixing entropy and graph connectivity) and biological signal conservation (cell type ASW) for each method. ASW, average silhouette width

A clear feature distinguishing scTour from currently available algorithms is its ability to jointly infer the pseudotime, vector field, and latent representations of cells, as well as to predict cellular dynamics of unobserved data (Fig. 5a). To benchmark scTour against widely used methods, I assessed each of these functionalities separately (excluding the prediction functionality which is not available in other tools). Specifically, scTour was compared with scVelo [8], Palantir [29], Monocle 3 [30], and Slingshot [31] for pseudotime estimation, with scVelo’s stochastic and dynamical models for vector field delineation, and with scVI [32] for latent space inference. The benchmarking was conducted based on the process of excitatory neuron development as illustrated above [25]. This process has well-described ground truth for pseudotime and vector field comparisons and the data displayed significant batch effects for latent space assessment (36,318 cells, Additional file 1: Fig. S16b).

Comparison of the pseudotime estimated by different tools highlighted the superiority of scTour in several aspects. Firstly, scTour more accurately recapitulated the continuous progression from cycling progenitors to mature excitatory neurons than did other methods, as evidenced by the higher correlation between the pseudotime estimates and the expression patterns of well-established marker genes along the trajectory (Fig. 5b). Secondly, scTour has no demand for specifying the starting cells which is required by Palantir, Monocle 3, and suggested by Slingshot. Although scVelo has no such requirement, the resulting pseudotime including the velocity pseudotime and latent time were not as accurate as that from scTour (Fig. 5b). Thirdly, the batch effects inherent in this data had minimal influence on scTour, but greatly impacted Monocle 3 and Slingshot as their performance dropped when batch correction was not performed prior (Fig. 5b).

At the level of the transcriptomic vector field, scTour was shown to both capture the underlying cellular dynamics and display considerably high consistency across neighboring cells (Fig. 5c). By contrast, the stochastic model from scVelo exhibited much lower consistency scores and its dynamical model erroneously directed migrating neurons towards progenitor cells (Fig. 5c).

With respect to the latent space, scTour was compared with scVI which likewise yielded latent representations of cells. As expected, without providing the batch information during model training, scTour was able to largely alleviate the influence of these batches and meanwhile preserve the intrinsic biological signals, as illustrated by the latent space-based UMAP visualization and by assessment of the batch mixing and biological conservation (Fig. 5d). By contrast, when the batch information was not considered and incorporated into the scVI model, the resulting latent space was dominated by sample batches, with cells from the same cell type segregated greatly across batches (Fig. 5d). Only after the batch factor was taken into account during modelling can scVI achieve the performance comparable to scTour (Fig. 5d).

To assess the performance of scTour when dealing with more complex topologies, I further benchmarked scTour against the other methods using a dataset profiling mouse gastrulation and early organogenesis [33] (Additional file 1: Fig. S17a). Again, scTour showed favorable performance in all three aspects: (1) the inferred vector field not only delineated the main cell lineages of mesoderm, endoderm, and ectoderm, but also the sub-lineages (Additional file 1: Fig. S17b). Specifically, scTour captured the developmental trajectory of the erythroid lineage (from haemato-endothelial progenitors, blood progenitors to erythroid) as well as the starting cells (epiblast) of the entire process, which were all reversed in the RNA velocity estimates by scVelo (Additional file 1: Fig. S17b). The remaining cells showed a high similarity between the velocity estimates from scTour and scVelo (Additional file 1: Fig. S17c). (2) The estimated pseudotime displayed the highest correlation with the known developmental stages when compared with that from scVelo and Palantir (Additional file 1: Fig. S17d,e). (3) The derived latent space showed comparable performance with scVI even when scVI took additional information (that is, batch factors) into consideration (Additional file 1: Fig. S17f,g).

### Characterization of human skeletal muscle development using scTour

**Fig. 6:**
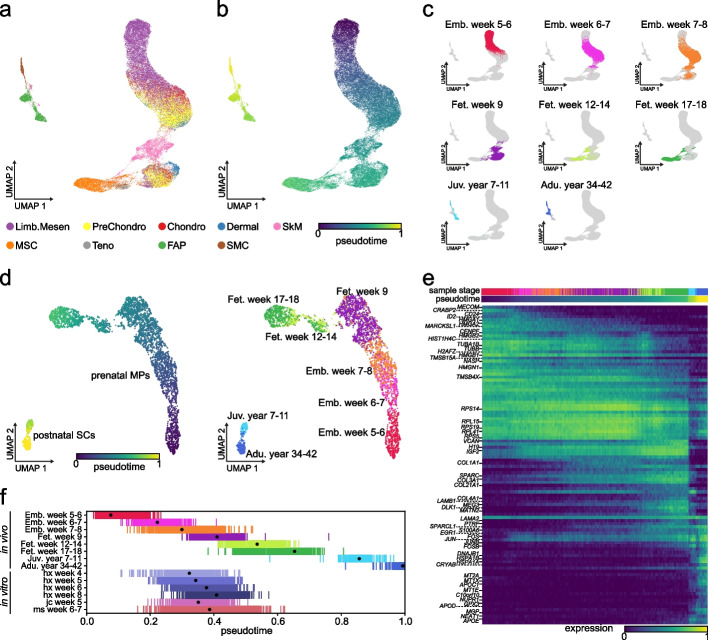
Application of scTour to human skeletal muscle development. **a** scTour’s latent space-based UMAP visualization of the cell types from all stages during human limb development (58,021 cells) [34]. Limb.Mesen, limb mesenchymal cells; PreChondro, prechondrogenic cells; Chondro, chondrogenic cells; Dermal, dermal fibroblasts; SkM, skeletal muscle cells; MSC, mesenchymal stromal cells; Teno, tenogenic cells; FAPs, fibro-adipogenic progenitors; SMC, smooth muscle cells. **b** As in **a**, but colored by the pseudotime estimated by scTour. **c** UMAP plots displaying cells from each of the developmental stages represented by different colors. **d** scTour’s latent space-based UMAP representations of the skeletal muscle progenitor and stem cells collected across prenatal and postnatal development (4816 cells), with colors indicating pseudotime estimates from scTour (left) and real developmental stages (right). **e** Heatmap illustrating the expression dynamics of top 100 most significant genes along the developmental trajectory. Developmental stages and estimated pseudotime are displayed on top. **f** Ordering of the in vivo skeletal muscle progenitor and stem cells based on the inferred pseudotime (upper), and ordering of the in vitro hPSC-derived progenitors based on the predicted pseudotime (lower). Dots denote the median values. Colors represent the developmental stages as in **d** (upper) and directed differentiation protocols and time points (lower)

To further showcase the biological insights that can be delivered through using scTour, I applied scTour to a challenging time series dataset which profiled the human limb muscle tissues over development from embryonic to adult stages (embryonic (prenatal weeks 5–8), fetal (prenatal weeks 9–18), juvenile (postnatal years 7–11), and adult (postnatal years 34–42)) [34]. In the original study, cells from each stage were analyzed separately, impeding the delineation of the whole developmental picture. Given scTour’s insensitivity to batch effects, it is possible to achieve the unbiased integration of cells across different time points to chart a biology-driven developmental trajectory. As a result, scTour reconstructed the full picture of human skeletal muscle ontogeny, with the diverse cell types chronologically placed in the low-dimensional space and the pseudotime estimates in keeping with the real developmental time (Fig. 6a–c). This trajectory also showed that the non-myogenic cell populations including mesenchymal, chondrogenic, and dermal fibroblast cells segregated between embryonic and fetal stages, indicating a pronounced transcriptomic change at this time window (Fig. 6a,c). The skeletal muscle (SkM) cells, on the other hand, displayed continuous changes during prenatal development, with major transcriptomic changes occurring postnatally (Fig. 6a,c).

One of the major directions the original study sought to explore was the molecular changes of the skeletal muscle progenitor and stem cells along development. By using scTour to particularly analyze those cells including the prenatal myogenic progenitors (MPs) and postnatal satellite cells (SCs), the gradual transcriptomic changes for MPs during prenatal development and the evident separation between MPs and SCs were revealed (Fig. 6d). There was a gap in the trajectory between cells from fetal week 9 and those from weeks 12–14, possibly corresponding to the missing sample stage in between (Fig. 6d). Different from the previous work where the progenitor and stem cells were divided and assigned to five developmental stages, the unbiased pseudotime estimates from scTour allowed the investigation of the continuous molecular changes underlying the developmental progression. Indeed, regression analysis of the gene expression changes along pseudotime identified previously undiscovered transcriptional patterns underpinning the cellular dynamics (Fig. 6e). For instance, genes related to cell differentiation including *MECOM*, *CRABP2*, *CD24*, and *ID2* [35–38] were specifically present in the earliest MPs (embryonic weeks 5–7) (Fig. 6e). Moreover, the three long noncoding RNAs identified (*H19*, *MEG3*, and *NEAT1*) were all involved in muscle differentiation [39] and displayed distinctive dynamics: *H19* was mainly expressed from late embryonic to fetal stages while *MEG3* was more enriched in late fetal and juvenile stages, whereas *NEAT1* was exclusive to postnatal SCs (Fig. 6e). For the postnatal SCs, they showed high expression of immediate early genes (*EGR1*, *FOS*, *JUN*, *JUNB*, and *FOSB*) and genes encoding heat shock proteins (*HSPA1A*, *HSPA1B*, and *DNAJB1*), indicating the early activation of those SCs induced by the cell isolation procedure during the experimental collections [40] (Fig. 6e). Besides, the SCs were enriched for genes associated with SC differentiation, regeneration, and survival (*SPARCL1*, *CRYAB*, and *GPX3*) [41–43], as well as genes involved in cell-cycle inhibition (*NUPR1*, *C10orf10/DEPP*) [44, 45] to maintain their quiescence (Fig. 6e).

Another major question the original study aimed to answer was the developmental status of the in vitro skeletal muscle progenitor cells (SMPCs) that were derived from human pluripotent stem cells (hPSCs) through different directed differentiation protocols (HX, JC, MS) [34]. This was partially solved in the earlier work by aligning the in vitro SMPCs with the in vivo progenitor and stem cells in the diffusion map space, as well as by scoring each cell based on genes enriched in postnatal versus embryonic stages [34]. Such an analysis, however, mapped in vitro SMPCs to a broad time window, that was, embryonic week 7 to fetal week 12 [34]. Moreover, how the status of these cells varied across different protocols and directed differentiation time points were not clear. To address these questions, I used scTour to predict the developmental pseudotime of each in vitro SMPC on the basis of the model trained with in vivo SMPCs. This provided a higher-resolution view of the in vitro-in vivo alignment, and revealed the discrepancy among directed differentiation protocols used and time points collected (Fig. 6f). Specifically, by first focusing on the cells from 4–8 weeks of in vitro differentiation under the HX protocol, a clear trend arose: the directed differentiation process followed the patterns of in vivo development, with cells from shorter differentiation time matching an earlier in vivo developmental phase (Fig. 6f). For instance, cells undergoing 4 weeks of in vitro differentiation corresponded to the stage of embryonic weeks 7–8 while those with in vitro differentiation time of 8 weeks aligned to fetal week 9 (Fig. 6f), providing a finer time window (7–9 weeks) compared to the original study (7–12 weeks). Further comparison of the in vitro SMPCs obtained from different protocols showed a high similarity between the JC and HX protocols, with cells derived from them (both collected at week 5) consistently mapping to the in vivo stage of embryonic/fetal weeks 7–9 (Fig. 6f). Different from them, the MS protocol yielded more heterogeneous populations at 6–7 weeks of in vitro differentiation, spanning a broader in vivo period from embryonic weeks 7–8 to fetal weeks 12–14 (Fig. 6f).

## Discussion

Here I present scTour, a novel deep learning architecture to perform multiple tasks in dissecting cellular dynamics. scTour starts from the raw gene expression matrix and ends with the full developmental dynamics revealed under a single framework, including the developmental pseudotime, vector field, and latent representations of cells. The resulting latent space, which is not available in many trajectory inference tools, offers information on trajectory reconstructions, cell stratifications, and data integrations. More importantly, all the inferences from scTour are largely unaffected by batch effects, and the ultimate estimates are driven by intrinsic biological signals. This presents a fascinating feature for exploring the cellular dynamics by integrating datasets from different studies, experimental platforms, and systems. scRNA-seq data integration has been a challenging task and scTour provides an easy way to approach this goal in the context of analysis of various dynamic processes.

The uniqueness of scTour also lies in its prediction functionalities comprising predicting cell characteristics given the transcriptomes and predicting the transcriptomic latent space given the time interval. This prediction is robust across biological systems, species, and experimental platforms and provides a convenient way for cross-data comparisons by propagating the information from existing datasets to new ones.

scTour also introduces an alternative way to calculate transcriptomic vector fields. Compared to the state-of-the-art RNA velocity [7, 8], scTour delivers several superiorities: (1) scTour does not require quantification of spliced and unspliced mRNAs, a rate-limiting but essential step in estimation of RNA velocity. (2) RNA velocity estimates can be affected by genes with partial or no kinetics captured [9, 11]. This has no impact on scTour’s vector field (Additional file 1: Fig. S10). (3) The application of RNA velocity to single-cell epigenetic data is not straightforward and to single-nucleus data is limited, due to the need to model transcriptional kinetics using spliced and unspliced reads. scTour overcomes these limitations as it relies only on the abundance matrix which quantifies the amount of transcripts/chromatin accessibilities across cells. It is thus applicable to datasets of both snRNA-seq (Additional file 1: Fig. S9) and scATAC-seq (Additional file 1: Fig. S18). (4) scTour’s vector field can be predicted based on the learned differential equation for a new dataset agnostic to the scTour model, a feature not available in RNA velocity-based tools. All these features broaden the use of vector field to decode dynamic processes with scTour.

Despite these advantages, scTour’s vector field cannot delineate the cycling processes in full compared to the RNA velocity (Additional file 1: Figs. S13b and S19). This is caused by the intrinsic property of scTour’s vector field which by definition follows the direction of time. It thus characterizes the dynamics over time in a noncyclic manner which cannot point cells with higher pseudotime to those with lower pseudotime (Additional file 1: Fig. S19). Also due to this property, scTour shows limitations in characterizing the absence of velocities across terminally differentiated cellular states where scTour displayed unexpected velocities following the invalid pseudotime ordering forcibly encoded by the distinct transcriptomes among cell types (Additional file 1: Fig. S20). This is possibly a common issue as RNA velocity also failed to unveil the absence of velocities (Additional file 1: Fig. S20). However, when scTour was applied to one terminal state exclusively, no dynamics were detected as expected (Additional file 1: Fig. S20).

When applying scTour to the developing mouse hippocampus dataset involving multiple branching events, the root state for this process was not defined unambiguously, with the astrocytes showing slightly lower pseudotime than the expected root of radial glia (Additional file 1: Fig. S2). This is probably due to the shared glia-like traits of radial glia and immature astrocytes that blur their transcriptomic distinctions and thus pseudotime ordering without the intervention from other complementary information such as unspliced RNAs. The other limitation of scTour is that due to the nature of continuous-in-time integration in the ODE solver, theoretically scTour achieves best performance for non-branching processes. Although it has been shown to accurately delineate the dynamics of various processes such as the bifurcation process in pyramidal and granule cell differentiation (Fig. 2b–g) and multifurcation process during early development (Additional file 1: Figs. S4 and S17), it may yield a unidirectional trajectory by sequentially connecting branches instead of parallelly handling each branch under some circumstances. Since the pseudotime is directly derived from the transcriptome through an encoder network in scTour, the age information imprinted in the data can to some extent alleviate this issue. In the future, the introduction of minimum spanning tree into the model when solving an ODE may further improve scTour’s performance in dealing with more complex topologies.

## Conclusions

scTour is an innovative and comprehensive method for dissecting cellular dynamics by analyzing datasets derived from single-cell genomics. It provides a unifying framework to depict the full picture of developmental processes from multiple angles including developmental pseudotime, vector field, and latent space, and further generalizes these functionalities to a multi-task architecture for within-dataset inference and cross-dataset predictions of cellular dynamics in a batch-insensitive manner. In this study, scTour’s new features and usefulness are obvious in multiple datasets. Combined with its robust performance with respect to batch effects and ability to scale to large datasets, scTour represents a broadly applicable strategy for multifaceted dissection of single-cell dynamics.

## Methods

### The scTour model

scTour models the cellular dynamics under the framework of VAE [13] and neural ODE [14]. By taking as input an abundance matrix (e.g., a gene expression matrix with $n$ cells and $g$ genes) $x\in {R}^{n\times g}$, for a single cell, a probabilistic encoder network ${f}_{z}$ with two fully connected (FC) layers is used (Additional file 2: Table S1) to approximate the posterior $q(z|x)$. It assumes a multivariate Gaussian with a diagonal covariance, with the mean $\mu$ and standard deviation $\sigma$ of the approximate posterior generated from ${f}_{z}$. $z$ is then sampled from $q(z|x)$ through the reparameterization trick [13]:

$$q\left(z|x\right)=\mathcal{N}(z;\mu ,{\sigma }^{2}\mathrm{\rm I})$$

$$\mu , \mathrm{log}{\sigma }^{2}={f}_{z}(x)$$

$$z \sim q(z|x)$$

$$z=\mu +\sigma \odot \epsilon$$

$${\text{where }} \epsilon \sim \mathcal{N}(0, \mathrm{\rm I})$$

A second encoder network ${f}_{t}$, composed of two FC layers (Additional file 2: Table S1) with the first hidden layer shared with ${f}_{z}$, transforms $x$ into a scalar time $t$ in the 0–1 range through the Sigmoid function. This corresponds to the developmental pseudotime of a given cell. By sorting cells based on their time $t$, the latent state $z$ at ${t}_{0}$ can be obtained. Next, given the initial state ${z}_{{t}_{0}}$ and times ${t}_{0},{t}_{1},{t}_{2},\dots ,{t}_{n}$ across cells, an ODE solver generates ${z}_{{t}_{1}}, {z}_{{t}_{2}},{\dots ,z}_{{t}_{n}}$ based on the differential equation (the derivative of the latent states with respect to time) which is defined by another neural network ${f}_{ode}$ (two FC layers, Additional file 2: Table S1):

$$t={f}_{t}\left(x\right)$$

$$\frac{dz(t)}{dt}={f}_{ode}(z(t))$$

$${z}_{{t}_{1}},{z}_{{t}_{2}},\dots ,{z}_{{t}_{n}}=\mathrm{ODESolve}({z}_{{t}_{0}},{f}_{ode},{t}_{0},{t}_{1},\dots ,{t}_{n})$$

$$\mathrm{Euler\,method\,for\,ODESolve}:z_{t_1}=z_{t_0}+f_{ode}(z_{t_0})\cdot (t_1-t_0)$$

$${z}_{{t}_{2}}={z}_{{t}_{1}}+{f}_{ode}\left({z}_{{t}_{1}}\right)\cdot \left({t}_{2}-{t}_{1}\right)$$

$$\dots$$

$${z}_{{t}_{n}}={z}_{{t}_{n-1}}+{f}_{ode}({z}_{{t}_{n-1}})\cdot ({t}_{n}-{t}_{n-1})$$

The “odeint” function within torchdiffeq [14] is used to perform this task. The default method for solving ODE in scTour is the Euler method, which solves the ODE under an initial value by taking a small step each time to estimate the value at the next time point based on the differential equation, under the assumption that the gradient does not change significantly along this small step.

Subsequently, the latent $z$ sampled from the approximate posterior, and the ${z}_{t}$ from the ODE solver parallelly go through a decoder network ${f}_{d}$ (two or three FC layers depending on the mode mentioned below, Additional file 2: Table S1) to reconstruct $x$. The objective function here is a modified lower bound:

$$\mathcal{L}=\alpha \cdot {\text{log}}p\left(x|z\right)+\left(1-\alpha \right)\cdot {\text{log}}p\left(x|{z}_{t}\right)-{D}_{\mathrm{KL}}(q(z|x)||p\left(z\right))-{\Vert z-{z}_{t}\Vert }_{2}^{2}$$

This equation combines the weighted reconstruction errors from both $z$ and ${z}_{t}$, the Kullback–Leibler (KL) divergence of the approximate posterior from the prior, and the mean squared error (MSE) between $z$ and ${z}_{t}$ as a regularizer to tune ${z}_{t}$ towards $z$. The KL divergence is defined here by considering the prior as the standard multivariate Gaussian [13, 46]:

$${D}_{\mathrm{KL}}\left(q\left(z|x\right)||p(z)\right)=\int q(z|x)\mathrm{log}\frac{q\left(z|x\right)}{p\left(z\right)}dz$$

$$\mathrm{where\,} p\left(z\right)=\mathcal{N}(z;0,\mathrm{\rm I})$$

In scTour, there are three modes to calculate the reconstruction errors ($\mathrm{log}p(x|z)$ and $\mathrm{log}p(x|{z}_{t})$), namely, MSE, negative binomial (NB)-conditioned likelihood [32, 47, 48], and zero-inflated negative binomial (ZINB)-conditioned likelihood [32, 48, 49]. MSE is a straightforward metric to measure the distance between the reconstructed and observed $x$, and exhibits good performance at a reduced cost of runtime.

$$\mathrm{MSE}:\mathrm{log}p\left(x|z\right)={-\Vert x-{\widetilde{x}}_{z}\Vert }_{2}^{2}$$

$$\mathrm{log}p\left(x|{z}_{t}\right)={-\Vert x-{\widetilde{x}}_{{z}_{t}}\Vert }_{2}^{2}$$

where ${\widetilde{x}}_{z}$ and ${\widetilde{x}}_{{z}_{t}}$ are the reconstructed $x$ based on $z$ and ${z}_{t}$, respectively.

The NB mode assumes a NB distribution for $p(x|z)$ to calculate the probability of observing the original input $x$ given the probability mass function of NB with its parameters estimated through the decoder network. Specifically, the gene-specific inverse dispersion is estimated as parameters in the neural network. Besides, the decoder network outputs the abundance proportion of each gene in a given cell via the softmax activation (Additional file 2: Table S1). The final reconstructed expression (the mean of the distribution) is obtained through multiplying this proportion by the library size which is approximated by summing the raw counts across genes within a cell here.

$$\mathrm{NB}:p\left(x=y\vert z\right)=\frac{\Gamma(r+y)}{\Gamma(r)\Gamma(y+1)}\left(\frac r{r+L\,\cdot \,d(z)}\right)^r\left(\frac{L\,\cdot \,d(z)}{r+L\,\cdot \,d(z)}\right)^y$$

$$p\left(x=y|{z}_{t}\right)=\frac{\Gamma (r+y)}{\Gamma (r)\Gamma (y+1)}{\left(\frac{r}{r+L\,\cdot \,d({z}_{t})}\right)}^{r}{\left(\frac{L\,\cdot \,d({z}_{t})}{r+L\,\cdot \,d({z}_{t})}\right)}^{y}$$

where $y$ represents the observed count, $r$ is the gene-specific inverse dispersion, *L* is the library size, and $d\left(z\right), d({z}_{t})$ are the abundance proportions of each gene within a certain cell decoded through $z$ and ${z}_{t}$, respectively.

The ZINB mode models the gene expression based on the assumption of a ZINB distribution. Similar with the NB mode, it estimates the dispersion and mean of the NB distribution using the decoder network. Additionally, it employs a neural network to decode the dropout probability as in scVI [32] and DCA [48] (Additional file 2: Table S1).

$$\begin{aligned}\mathrm{ZINB}:&\left\{\begin{array}{c}p\left(x=0\vert z\right)=\pi_z+(1-\pi_z)\left(\frac r{r+L\,\cdot \,d(z)}\right)^r\\p\left(x=y\neq0\vert z\right)=(1-\pi_z)\frac{\Gamma(r+y)}{\Gamma(\mathrm r)\Gamma(\mathrm y+1)}\left(\frac r{r+L\,\cdot \,d(z)}\right)^r\left(\frac{L\,\cdot \,d(z)}{r+L\,\cdot \,d(z)}\right)^y\end{array}\right.\\ &\left\{\begin{array}{c}p\left(x=0\vert z_t\right)=\pi_{z_t}+(1-\pi_{z_t})\left(\frac r{r+L\,\cdot \,d(z_t)}\right)^r\\p\left(x=y\neq0\vert z_t\right)=(1-\pi_{z_t})\frac{\Gamma(r+y)}{\Gamma(\mathrm r)\Gamma(\mathrm y+1)}\left(\frac r{r+L\,\cdot \,d(z_t)}\right)^r\left(\frac{L\,\cdot \,d(z_t)}{r+L\,\cdot \,d(z_t)}\right)^y\end{array}\right.\end{aligned}$$

where $y$, $r$, *L*, $d\left(z\right), d({z}_{t})$ have the same definitions as in the NB mode, and ${\pi }_{z}$, ${\pi }_{{z}_{t}}$ represent the dropout probability decoded from $z$ and ${z}_{t}$, respectively.

All the hidden layers use ReLU as the activation function except for the neural net ${f}_{ode}$ where ELU [50] is used (Additional file 2: Table S1).

### Model training, inference, and prediction

#### Model training

scTour takes the abundance matrix (cell by gene for sc/snRNA-seq and cell by peak for scATAC-seq) as input. Depending on the mode chosen for reconstructing the input, the abundance matrix is required to be log-transformed normalized expression (for MSE mode) or raw counts (for NB and ZINB modes). Selection of highly variable genes is suggested before model training to reduce runtime and improve the performance. Although the application of mini-batches in neural ODE is less straightforward [14], mini-batch training suits the scTour architecture quite well, which offers a number of advantages. Specifically, mini-batch training makes direct backpropagation more feasible, model training faster, and memory more efficient. These together endow scTour with the great scalability to large datasets. Importantly, with mini-batch training, scTour is able to achieve high performance using only a subset of cells sampled. The batch size is set to 1024 throughout the paper and can be adjusted depending on datasets. The default method for solving ODE is “Euler,” with additional methods provided by “torchdiffeq” [14] available through the parameter “ode_method.” The hyperparameter $\alpha$ in the objective function for balancing the reconstruction errors derived from $z$ and ${z}_{t}$ is 0.5 by default, and is adjustable depending on datasets. For linear processes, the inferred developmental pseudotime, transcriptomic vector field, and latent representations are robust to the choice of $\alpha$ (Additional file 1: Fig. S21). For branching processes, the derived pseudotime is insensitive to $\alpha$, but the latent representation and vector field inferred from a small $\alpha$ (with a large weight assigned to the reconstruction error from ${z}_{t}$) tend to order the cells based on their pseudotime and thus cannot well separate cell types with similar developmental orders (Additional file 1: Fig. S22). Under such circumstances, a larger weight is needed for the reconstruction error from $z$ (at least 0.4 for the demonstration dataset of pyramidal neuron and granule cell development, Additional file 1: Fig. S22). For the optimization, scTour uses Adam [51] as the optimizer (learning rate is set to 0.001), with the L2 regularization implemented to strengthen model generalization (weight decay is set to 1e − 6). Since scTour converges faster for large datasets versus small ones, the default number of epochs in scTour is proportional to the number of cells in the dataset of interest. The default parameters for model training are listed in Additional file 3: Table S2.

#### Subsampling-based training

scTour provides the option to train the model with a subset of cells. Specifically, scTour first shuffles the entire dataset and then randomly samples a given proportion of cells from the shuffled data. The two rounds of randomness ensure the preservation of the cellular diversity. This step reduces the training time and has marginal influence on the model performance as shown in multiple datasets.

#### Cellular dynamics inference

After the model training, scTour assigns a developmental pseudotime to each cell based on the learned time neural net ${f}_{t}$ without the need for specifying starting cells. Since there exist two possible integration directions (forward or backward), the inferred pseudotime can be in the correct ordering (ascending), or the reverse (descending). To resolve this, scTour leverages the information of gene counts (i.e., the number of expressed genes) across cells which are demonstrated to correlate with developmental potential [52]. Specifically, a linear regression model is fit between the inferred pseudotime and the gene counts. If the slope is positive, the estimated time will be reversed, and the downstream predictions will be reversed as well. In the cases where the use of gene counts fails to capture the expected trend, scTour provides a post-inference function to reverse the pseudotime.

The transcriptomic vector field is the learned differential equation ${f}_{ode}$, which outputs the gradient given the current latent state and thus provides information regarding the future transcriptomic directions.

The latent representations of cells in scTour are the weighted combination of $z$ from the variational inference and ${z}_{t}$ from the ODE solver:

$$z_{latent}=\omega\cdot z+(1-\omega)\cdot z_t$$

Larger $\omega$ skews the latent space towards the intrinsic transcriptomic structure while smaller $\omega$ is more representative of the extrinsic pseudotime ordering (Additional file 1: Figs. S23-26). Users of scTour have the option to adjust $\omega$ according to their purposes.

#### Cellular dynamics prediction

Given the gene expression matrix of query cells from an unobserved cellular state or a new dataset, scTour predicts their developmental pseudotime by the time neural net ${f}_{t}$, transcriptomic vector field by the function ${f}_{ode}$, and latent representations by the whole framework built from reference cells.

Regarding the prediction of the transcriptomic space given an unobserved time interval ${t}_{1}$, ${t}_{2}$, …, ${t}_{n}$, scTour takes a stepwise integration given the learned differential equation ${f}_{ode}$ by leveraging the *k*-nearest neighbors. Specifically, the developmental pseudotime $T$ and the latent representations $Z$ from the training data are used as a reference. Next, for each time point $t$ within the unobserved interval, its *k*-nearest neighbors in the reference are obtained by comparing $t$ with $T$. Next for each neighbor $j$, the ODE solver takes the latent state of this neighbor ${z}_{j}$ as the initial value, together with the time of this neighbor ${t}_{j}$ and the time $t$, to output the latent state corresponding to $t$. The final latent representation of the time $t$ is calculated as the average across the *k*-nearest neighbors:

$${z}_{t}=\frac{1}{k}\sum_{j}{\text{ODESolve}}({z}_{j},{f}_{ode},{t}_{j},t)$$

For each time point estimated, the resulting latent state ${z}_{t}$ along with the time $t$ are added to the latent state $Z$ and time $T$ pool to update the reference for predicting the next time point. This procedure is stopped until the entire time span has been predicted.

### Visualization of the vector field

The visualization of the transcriptomic vector field on a low-dimensional embedding such as UMAP is obtained using a similar approach as in velocyto [7] and scVelo [8]. The main idea is to position the velocity arrow in the direction where the estimated velocity best matches the transcriptomic difference. To this end, a cell–cell transition probability matrix $P$ is first calculated. Different from velocyto and scVelo which calculate this matrix using the gene-based velocity vector and the gene expression difference, scTour computes the matrix at the level of latent space. Specifically, based on the vector field derived from the learned differential equation ${f}_{ode}$ and the latent state of each cell, scTour calculates the cosine similarity between the gradient and the latent difference:

$${P}_{ij}=\mathrm{exp}\left(\frac{\mathrm{cos}({v}_{i},{l}_{ij})}{\sigma }\right)$$

$${v}_{i}={f}_{ode}({z}_{i})$$

$${l}_{ij}={z}_{j}-{z}_{i}$$

where ${v}_{i}$ is the gradient of cell $i$ inferred from the learned differential equation ${f}_{ode}$ given its latent state ${z}_{i}$, and ${l}_{ij}$ represents the difference between cell $i$ and $j$ at the latent space level. Both ${v}_{i}$ and ${l}_{ij}$ can be optionally transformed using variance-stabilizing transformations before calculating the cosine similarity. Similar with scVelo, for each cell, only the recursive neighbors from the KNN graph are considered for cell–cell transition probability estimation. Differently, scTour also considers the neighbors in the time space based on the developmental pseudotime inferred for each cell. The resulting transition probability matrix $P$ is next row-normalized to let $\sum_{j}{P}_{ij}=1$. The normalized matrix is used as weights to calculate the displacement vector for each cell:

$$\Delta u=\sum_{j\ne i}({P}_{ij}-\frac{1}{n})\frac{{u}_{j}-{u}_{i}}{\Vert {u}_{j}-{u}_{i}\Vert }$$

where ${u}_{i}$ and ${u}_{j}$ are the coordinates of cells $i$ and $j$ in the low-dimensional embedding, and subtracting $\frac{1}{n}$ controls for the non-uniform density of the data points (neighboring cells $j$ of cell $i$) under the embedding. This displacement vector can be visualized for each cell or at the grid level as arrows or streamlines.

### Analysis of mouse dentate gyrus neurogenesis

The two datasets from the mouse dentate gyrus used in Fig. 2 were from [15]. For the first dataset, the raw count matrix and meta information were downloaded from Gene Expression Omnibus (GEO) under the accession number GSE95315 [53]. Only the cell types along the granule cell lineage including nIPCs, neuroblasts (Neuroblast_1, Neuroblast_2), and immature and mature granule cells were used for the following analysis (4007 cells). Before running scTour, the data was preprocessed by filtering genes detected in less than 20 cells and selecting the top 500 highly variable genes using Scanpy [54]. A scTour model was then trained with the raw count matrix from these 500 genes across 4007 cells. The resulting model was used to infer the developmental pseudotime, transcriptomic vector field, and latent representations of these cells (the latent space was generated with 20% $z$ and 80% ${z}_{t}$). UMAP embeddings derived from the inferred latent space and PCA space (40 PCs) were compared. For the comparison of the vector field between scTour and scVelo in Additional file 1: Fig. S1, the cells from the two time points P12 and P35 which were used in the scVelo publication were extracted to run scTour and scVelo.

For the second dataset downloaded from GEO (GSE104323) [55], the cells from the granule cell lineage (nIPCs, neuroblast, immature and mature granule cells) and the pyramidal neuron lineage (immature pyramidal neurons) were considered (15,174 cells). Similarly, genes detected in less than 20 cells were excluded and the top 2000 highly variable genes were used for the scTour model training, which yielded the developmental pseudotime, vector field, and latent space (40% $z$ and 60% ${z}_{t}$) of cells. The latent space from scTour and PCA space (30 PCs) were used to calculate the UMAP embeddings for comparisons. To demonstrate the robustness of scTour to cell subsampling, the models were trained based on cell subsets from 1 to 95% of all cells. The resulting models were used to infer the dynamics (developmental pseudotime, vector field, and latent representations) of all cells. Spearman correlation coefficients between the developmental pseudotime derived from the models trained with < 95% of all cells and that from the model trained with 95% of cells were calculated to show the stable inference.

For the dataset shown in Additional file 1: Fig. S2 which incorporated more cell lineages in the developing mouse hippocampus [7], the meta information and raw count matrix were downloaded from the scVelo package (18,213 cells). For the scTour model training, top 2000 highly variable genes and 20% of cells randomly sampled were used to derive the pseudotime, transcriptomic vector field, and latent representation (90% $z$ and 10% ${z}_{t}$) for the entire dataset. The parameters for the contributions of reconstruction errors from latent $z$ and ${z}_{t}$ in the objective function were set to 0.8 and 0.2, respectively.

### Analysis of mouse pancreatic endocrinogenesis

The dataset from the mouse pancreatic endocrine development [8, 23, 56] used in Fig. 3 was downloaded from the scVelo package. The scTour model training started from the raw count matrix including the top 2000 highly variable genes and 3696 cells, and ended with the estimated developmental pseudotime, transcriptomic vector field, and latent representations (70% $z$ and 30% ${z}_{t}$) of the cells. To compare scTour’s pseudotime with scVelo’s latent time, as well as to compare scTour’s vector field with the RNA velocity from scVelo and κ-velo, the same procedures as in the original scVelo and κ-velo publications were used to reproduce the results. When running κ-velo, the step of prior knowledge filtering was skipped to be comparable with scTour and scVelo’s velocity estimates which were obtained with no prior knowledge provided.

To test the ability of scTour to predict the dynamics of unseen cellular states, the model was trained by excluding one of the cell types and the resulting model from the remaining cell types was used for two purposes: (1) predicting the developmental pseudotime, transcriptomic vector field, and latent representation of the excluded cell type given its gene expression matrix. The accuracy of the prediction for the pseudotime was quantified through calculating the area under the receiver operating characteristic (ROC) curve (AUC) for the binary classifications between the held-out cell type and those preceding or after it. This calculation was based on the predicted pseudotime for the held-out cell type and the inferred pseudotime for the remaining ones using the functions “sklearn.metrics.roc_curve” and “sklearn.metrics.auc” provided in scikit-learn [57]. (2) Predicting the latent representation of the excluded cell type given its expected developmental time along the differentiation path. The comparison of the predicted latent representation with the ground truth (the latent space of the excluded cell type derived from its gene expression matrix) was performed from three angles. Firstly, the predicted latent space, together with the latent space of all cell types during endocrinogenesis, were combined to yield a UMAP embedding. Secondly, the pairwise Euclidean distance was calculated between the predicted latent representation and the latent representation of each cell type. Lastly, unsupervised hierarchical clustering was conducted based on the predicted latent space and the latent space of all the cell types (Euclidean distance as the distance metric and “ward” as the linkage algorithm).

### Analysis of cortical excitatory neuron development

Datasets profiling the cortical excitatory neuron development used in Fig. 4 were from four sources: (1) the developing human cortex measured using 3′ Kit v3 protocol of 10x Genomics [25, 58]. Here I focused on the same set of cells which were used in the original study to reconstruct the excitatory neuron developmental trajectory (36,318 cells). (2) The developing human cortex measured using Drop-seq [26, 59], with the cell types of cycling progenitors, intermediate progenitors, migrating neurons, maturing neurons, and upper and deep layer excitatory neurons (27,855 cells) considered here. (3) The human brain organoid measured using 3′ Kit v2 of 10x Genomics [27, 60]. Here I focused on the cells of cycling progenitors, intermediate progenitors, immature and mature excitatory neurons from the organoids cultured for 3 months (3-month PGP1 organoids 1–3, 16,032 cells). (4) The developing mouse cortex measured using 3′ Kit v2 of 10x Genomics [28, 61]. The cells of apical progenitors, intermediate progenitors, migrating neurons, immature neurons, and excitatory neurons from different layers with different projection properties (73,649 cells) were used.

For the first dataset, since the excitatory neuron subtypes in the original study were labelled with arbitrary numbers, I relabelled those cells according to the second dataset where the excitatory neuronal cells were named on the basis of their maturity along the differentiation path. Specifically, CellTypist [62] was used to train a model based on the reference dataset (i.e., the second one), which was subsequently employed to transfer the cell type labels to cells of the first one.

The scTour model was then trained based on the first dataset (training data) by using 60% of all cells, and 765 genes which were the intersection of the top 1000 highly variable genes from this data with the genes detected in all the other three datasets (test data). This model was used to infer the developmental pseudotime, transcriptomic vector field, and latent space (50% $z$ and 50% ${z}_{t}$) of the training data (Fig. 4a), and to predict the properties of cells from the test data (Fig. 4b–d). For the UMAP embeddings of the three test datasets shown in Fig. 4b–d, the first two were derived from PCA space (30 PCs) and the last one was batch corrected using BBKNN [63] to mitigate the substantial batch effects among donors. For the UMAP embeddings of the three test datasets shown in Additional file 1: Fig. S16c-e, they were all derived from the predicted latent space by scTour without any batch corrections.

### Analysis of other biological processes

In addition to the developmental courses mentioned above, scTour was applied to a number of dynamic biological processes described as follows.

#### Mouse gastruloid

This dataset (30,496 cells) came from a study on embryonic gastruloids measured using 10x Genomics [16, 64]. The cell type classification and UMAP embedding from the original study were used as is here. The developmental pseudotime, transcriptomic vector field, and latent representations (70% $z$ and 30% ${z}_{t}$) of these cells were inferred from the scTour model which was trained with 2000 highly variable genes and 60% of cells randomly sampled from the whole data.

#### Human thymic epithelial cell development

This dataset (14,217 cells) profiled the human thymic epithelial development using 10x Genomics [17, 65]. The cell annotations and UMAP embedding from the publication were used as is. The highly variable genes from the original study (804) and cells randomly sampled from the whole data (60%) were used to train the scTour model, which generated the developmental pseudotime, transcriptomic vector field, and latent representations (70% $z$ and 30% ${z}_{t}$) of all cells.

#### Human gastrulation

This dataset (1195 cells) was from a gastrulating human embryo measured using Smart-seq2 [18, 66]. The cell annotations and UMAP embedding from the original study were used here. For the scTour model training, the top 2000 highly variable genes were considered. The trained model was then used to infer the developmental pseudotime, vector field, and latent representations (80% $z$ and 20% ${z}_{t}$) for these cells.

#### Human preimplantation

This dataset has 90 cells from human preimplantation embryos with single cells isolated by the mouth pipette [19, 67]. For the PCA-based UMAP embedding, the top 30 PCs derived from the 2000 highly variable genes were used. The developmental pseudotime, transcriptomic vector field, and latent representations (70% $z$ and 30% ${z}_{t}$) of these cells were inferred from the scTour model trained with the same set of genes.

#### Reprogramming in mouse

This dataset (251,203 cells) was from a time course of iPSC reprogramming measured using 10x Genomics [20, 68]. The original cell annotations and force-directed layout embedding (FLE) from the publication were used here. The scTour model was trained based on 2000 highly variable genes and 20% of cells, which produced the developmental pseudotime, transcriptomic vector field, and latent representations (30% $z$ and 70% ${z}_{t}$) of all cells.

#### Reprogramming in human

This snRNA-seq dataset (36,597 nuclei) was from a study on human cell reprogramming [21, 69]. Similarly, the cell annotations and UMAP embedding provided by the original study were used to visualize the estimated developmental pseudotime and transcriptomic vector field from the scTour model trained on the basis of 2000 highly variable genes and 60% of all cells. The inferred latent space from the same model (70% $z$ and 30% ${z}_{t}$) was used to generate a new UMAP embedding to illustrate the reprogramming trajectory.

#### Human hematopoiesis

This scNT-seq dataset (1947 cells) was from in vitro culture of the CD34 + human hematopoietic stem and progenitor cells (HSPCs) [9, 70]. The gene set (1956 genes) from the original study was used to train the scTour model, which yielded the pseudotime, transcriptomic vector field, and latent representations (80% $z$ and 20% ${z}_{t}$) of all cells. The cell annotations and UMAP embedding from the publication were used here for visualization.

#### Brain endothelial topography

This dataset (3105 cells) was focused on the endothelial cells of the mouse brain [22, 71]. To be consistent with the original study, the three subclusters (choroid plexus, artery shear stress, and interferon) were excluded from the differentiation trajectory reconstruction. The PCA space-based UMAP embedding was from the top 30 PCs which were obtained from the 2000 highly variable genes. The trajectory reconstruction by scTour (developmental pseudotime, transcriptomic vector field, latent representations (20% $z$ and 80% ${z}_{t}$)) was based on the same set of genes.

#### Mouse retina development

This dataset (2726 cells) was from the E15.5 mouse retina [72, 73] and downloaded from http://pklab.med.harvard.edu/peterk/review2020/ [74]. The scTour model was trained based on 2000 highly variable genes, and the resulting pseudotime and vector field were visualized in the UMAP embedding provided by [74]. For obtaining the RNA velocity from scVelo, the procedures shown in scVelo tutorials were followed.

#### Human fetal retinal chromatin accessibility

This scATAC-seq dataset (4883 cells) was from the fetal human retina [75, 76]. Preprocessing of this dataset was conducted using Signac [77], including normalization by term frequency-inverse document frequency (TF-IDF), feature selection (top 25% of peaks), and dimension reduction by singular value decomposition. The first latent semantic indexing (LSI) component which was highly correlated with sequencing depth was excluded and the 2–30 components were used for UMAP embedding calculation. The TF-IDF matrix (34,670 genomic regions across 4883 cells) was used as input for the scTour model training, which generated the developmental pseudotime, epigenetic vector field, and latent representations (50% $z$ and 50% ${z}_{t}$) for these cells.

### Benchmarking scTour against existing algorithms

To benchmark scTour against existing methods including scVelo [8], Palantir [29], Monocle 3 [30], Slingshot [31], and scVI [32], the dataset profiling the developing human cortex by 10x Genomics (D1, 36,318 cells) [25] as well as the dataset from mouse gastrulation and early organogenesis (D2, 89,267 cells) [33, 78] were used. The second dataset was downloaded from the scVelo package. For all the analyses (pseudotime, vector field, and latent space) performed by these tools, the top 1000 highly variable genes were considered for D1 and 2000 for D2. The analytical procedure for each method is described as follows.

#### scTour

For each dataset, 20% of cells randomly sampled from the entire data were used to train the scTour model, which yielded the developmental pseudotime, transcriptomic vector field, and latent representations (five dimensions; 50% $z$ and 50% ${z}_{t}$ for D1 and 60% $z$ and 40% ${z}_{t}$ for D2) of all cells. During model training for D2, the parameters adjusting the contributions of reconstruction errors from latent $z$ and ${z}_{t}$ in the objective function were set to 0.7 and 0.3, respectively.

#### scVelo

For D1, both the stochastic and dynamical models were performed following the tutorials at https://scvelo.readthedocs.io, with the velocity pseudotime derived from the stochastic model and the latent time derived from the dynamical model. For D2, only the RNA velocity and velocity pseudotime from the stochastic model are shown in Additional file 1: Fig. S17b,d given its better performance for this dataset compared to the dynamical model.

#### Palantir

The pseudotime estimation was conducted based on the tutorial at https://nbviewer.org/github/dpeerlab/Palantir/blob/master/notebooks/Palantir_sample_notebook.ipynb. Forty PCs were considered during the diffusion map construction, and the cell expressing the highest level of *PAX6* (for D1) or *Dnmt3b* (for D2) was designated as the starting cell when determining the pseudotime.

#### Monocle 3

The Seurat Wrappers package was used to run Monocle 3 for D1 on the Seurat objects with or without batch effect correction, respectively. To obtain the batch-corrected Seurat object, the procedures from the tutorial at https://satijalab.org/seurat/articles/integration_introduction.html were followed [79]. The starting cell was specified as the one with the highest expression level of *PAX6* (D1) when estimating the pseudotime.

#### Slingshot

The pseudotime was estimated for D1 based on a two-dimensional UMAP embedding (derived from 30 PCs) and a vector of clustering labels (from Louvain clustering with a resolution of 0.2). The starting cluster was set to the one with the highest proportion of cycling progenitor cells. The final pseudotime was calculated as the average across the lineages. To rerun Slingshot by removing batch effects, the integration process from Seurat as described above was conducted to obtain batch-corrected UMAP and clustering labels for pseudotime estimation.

#### scVI

The scVI model training was performed using the default parameters to generate a 10-dimensional latent space. This was run twice, with or without batch information provided, respectively. For D2, an additional factor “stage” was provided as a covariate along with the factor “sequencing.batch” provided as the batch key.

To examine the accuracy of the pseudotime estimated from different methods, for D1, the well-established marker genes along the excitatory neuron developmental trajectory were collected, including the markers for progenitors (*GLI3*, *TFAP2C*, *PAX6*, *SOX2*, *EOMES*, *JUND*, *NFE2L2*, *SOX9*, *EMX2*, and *FOS*) and excitatory neurons (*MEF2C*, *SATB2*, *STMN2*, *NEUROD2*, *NEUROD6*, *BHLHE22*, *POU2F2*, *ZBTB18*, *CHD3*, and *MYT1L*) [25, 26]. Spearman correlation coefficient was then calculated between the pseudotime estimates and the expression profiles of each of these genes. For D2, Spearman correlation coefficient was computed between the pseudotime estimates and the known developmental stages.

To check the consistency of the vector field across neighboring cells, consistency score, the same metric as defined in the scVelo publication, was computed here, which was the mean Pearson correlation coefficient calculated between the vector field of a given cell and its neighbors. To examine the correlation between scTour’s vector field and the RNA velocity, the cosine similarity was computed based on the projected velocities under the same UMAP embedding, that is, the weighted combination of unitary displacement vectors obtained from scTour and scVelo.

To evaluate the degree of batch correction and biological signal conservation, three metrics from scArches [80] and scIB [81] were used. The first one was the entropy of batch mixing, which measured the batch diversity in the neighboring cells. Fifteen nearest neighbors were considered for each cell. The second metric was the graph connectivity, which estimated the connectivity among all the cells in each cell type. These two metrics were used to assess the batch correction. The third metric was the cell type average silhouette width (ASW), which calculated the inter-cluster (the nearest cluster) versus intra-cluster distances and was used to assess the biological signal conservation.

### Analysis of human skeletal muscle development

The scRNA-seq data profiling the human limb muscle tissues across prenatal and postnatal development, as well as the hPSC-derived in vitro muscle cells from different protocols was downloaded at skeletal-muscle.cells.ucsc.edu [34, 82]. To investigate the full dynamics of human skeletal muscle ontogeny, a scTour model was trained using cells collected from all the developmental stages. The cell types with less than 1000 cells (skin cells, red blood cells, Schwann cells, white blood cells, and endothelial cells) were excluded from the model training, resulting in 58,021 cells as input for scTour. The training was done based on the top 2000 highly variable genes and 90% of all cells for 200 epochs, and the resulting model was used to infer the pseudotime and latent space (50% $z$ and 50% ${z}_{t}$) for all the cells.

Next, another scTour model was trained focusing on the skeletal muscle progenitor and stem cells (4412 prenatal myogenic progenitors and 404 postnatal satellite cells). The training was conducted on the basis of 90% of all cells, and 1791 genes which were the intersection of the top 2000 highly variable genes with genes expressed in the in vitro skeletal muscle progenitors. The resulting model was used for two purposes: (1) to infer the pseudotime and latent space (50% $z$ and 50% ${z}_{t}$) of the in vivo progenitor and stem cells; (2) to predict the pseudotime of the in vitro progenitor cells derived from different directed differentiation protocols and time points (14,996 cells). To identify genes showing dynamic expression changes along the trajectory, the same method defined in Monocle [6] was used. In detail, for each gene the cross-cell expression level was modelled as a function of the cells’ pseudotime by using cubic smoothing spline with three degrees of freedom in the R package VGAM [83]. The significance was estimated by the likelihood ratio test which compared the full model with the reduced model (i.e., intercept-only regression), with the *p*-value adjusted by the Bonferroni correction. The top 100 most significant genes are shown in Fig. 6e.

### Analysis of datasets with terminally differentiated cellular states

Two datasets with only terminally differentiated cells were used to test the ability of scTour’s vector field to detect the absence of velocity: PBMC 3 k (from 10x Genomics) [84] and human decidua [85, 86]. For the PBMC 3 k dataset, the data was downloaded from Scanpy. The scTour model was trained based on 2638 cells and 2000 highly variable genes to derive the pseudotime and vector field. Two additional models were trained based only on B cells (342 cells and 1000 highly variable genes) or CD14 + monocytes (480 cells and 1000 highly variable genes) to show the ability of scTour’s vector field to identify the absence of velocity when applied to only one terminal state. To estimate the RNA velocity using scVelo, the bam file was downloaded from 10x Genomics (https://support.10xgenomics.com/single-cell-gene-expression/datasets/1.1.0/pbmc3k) and velocyto [7] was used to obtain the spliced and unspliced count matrices as input for the stochastic model in scVelo. For the human decidua dataset, the raw count matrix and metadata were downloaded from ArrayExpress under the accession number E-MTAB-6701. Only cells from decidual stroma (dS), natural killer (dNK), T, and macrophages (dM) in donor D8 were considered here (7487 cells). The scTour model was trained based on 2000 highly variable genes to infer the pseudotime and vector field. As in the PBMC dataset, two more models were trained for the subtype dS1 (1386 cells, 1000 highly variable genes) and dNK2 (550 cells, 1000 highly variable genes), respectively. To obtain the RNA velocity, the fastq files were downloaded from E-MTAB-6701 and cellranger (version 6.0.1) together with velocyto were used to output the spliced and unspliced count matrices for running scVelo.

### Assessment of the parameter $\alpha$ in the objective function

To assess the impact of the parameter $\alpha$ in the objective function (balancing the reconstruction errors derived from $z$ and ${z}_{t}$) on the inferred cellular dynamics, I compared the developmental pseudotime, transcriptomic vector field, and latent representations derived from the scTour models trained using a series of $\alpha$ values (from 0.1 to 0.9 with a step size of 0.1) based on the two datasets shown in Fig. 2. Specifically, Spearman correlation coefficients were calculated between the pseudotime estimated with the default $\alpha$ (0.5) and those from other $\alpha$ values. To evaluate the vector field inferred, cosine similarities were computed between the projected velocities from the default $\alpha$ and those from other $\alpha$ values under the same UMAP embedding (the UMAP embedding from the default $\alpha$). For the latent representations, the cell type ASW was measured under each $\alpha$ value to quantify the biological signal conservation.

### Assessment of contributions of $z$ and ${z}_{t}$ to scTour’s latent representation

To assess the contributions of $z$ (weighted by $\omega$, 0.5 by default) and ${z}_{t}$ (weighted by $1-\omega$) when defining the final latent representation, the latent representations obtained by different combinations of $z$ and ${z}_{t}$ (with $\omega$ set from 0 to 1 with a step size of 0.1) inferred from the same model were compared. Here four datasets were used: granule cell development in the mouse dentate gyrus (linear process, Additional file 1: Fig. S23), excitatory neuron development in the human cortex (linear process, Additional file 1: Fig. S24), granule cell and pyramidal neuron development in the mouse hippocampus (bifurcation process, Additional file 1: Fig. S25), and endocrinogenesis in the mouse pancreas (multifurcation process, Additional file 1: Fig. S26). Cell type ASW was calculated for each combination to quantify the biological signal conservation.

## Supplementary Information

**Additional file 1: Fig. S1.** Inferred vector field of cells during granule cell differentiation in the mouse dentate gyrus using scTour and scVelo. **Fig. S2.** Application of scTour to the developing mouse hippocampus dataset. **Fig. S3.** scTour’s inference is robust to cell subsampling. **Fig. S4.** scTour captures the developmental cellular dynamics in embryonic organoids. **Fig. S5.** scTour captures the developmental cellular dynamics in the human thymic epithelial cells. **Fig. S6.** scTour captures the developmental cellular dynamics in human gastrulation. **Fig. S7.** scTour captures the developmental cellular dynamics in human preimplantation. **Fig. S8.** scTour captures the cellular dynamics during iPSC reprogramming in mice. **Fig. S9.** scTour captures the cellular dynamics during iPSC reprogramming in humans. **Fig. S10.** scTour captures the developmental cellular dynamics during hematopoiesis. **Fig. S11.** scTour captures the anatomical topography of brain endothelial cells. **Fig. S12.** Superiority of scTour’s pseudotime over scVelo’s latent time in characterizing a discontinued process. **Fig. S13.** Comparisons of scTour’s vector field with velocities from other methods. **Fig. S14.** scTour predicts the cellular dynamics of unseen cellular states regardless of their positions along the developmental process. **Fig. S15.** scTour reconstructs the transcriptomic space at different developmental stages. **Fig. S16.** scTour predicts the latent space of unseen datasets. **Fig. S17.** Benchmarking scTour against existing methods using the dataset profiling mouse gastrulation and early organogenesis. **Fig. S18.** Application of scTour to scATAC-seq data. **Fig. S19.** Delineation of the cycling process by scTour’s vector field and RNA velocity. **Fig. S20.** Application of scTour to terminally differentiated cells. **Fig. S21.** Assessment of the parameter alpha in the objective function in a dataset of granule cell differentiation. **Fig. S22.** Assessment of the parameter alpha in the objective function in a dataset of pyramidal neuron and granule cell development. **Fig. S23.** Assessment of contributions of *z* and *z*_t_ to scTour’s latent representation in a dataset of granule cell development. **Fig. S24.** Assessment of contributions of *z* and *z*_t_ to scTour’s latent representation in a dataset of excitatory neuron development. **Fig. S25.** Assessment of contributions of *z* and *z*_t_ to scTour’s latent representation in a dataset of granule cell and pyramidal neuron development. **Fig. S26.** Assessment of contributions of *z* and *z*_t_ to scTour’s latent representation in a dataset of pancreatic endocrinogenesis.

**Additional file 2: Table S1.** Neural networks used in scTour.

**Additional file 3: Table S2.** Hyperparameters in scTour model training.

**Additional file 4: Table S3.** Summary of the datasets used in this study.

**Additional file 5.** Review history.

## Data Availability

The source code of scTour is available on GitHub under a MIT license (https://github.com/LiQian-XC/sctour) [87], with the version used in the manuscript also deposited in Zenodo (10.5281/zenodo.7538567) [88]. All the datasets (21 datasets) used and referred in this study are publicly available and also summarized in Additional file 4: Table S3.
