# Supplementary material for: scTour: a deep learning architecture for robust inference and accurate prediction of cellular dynamics
Source: Genome Biol. 2023 Jun 23;24:149. doi: 10.1186/s13059-023-02988-9 (PMC10290357; doi:10.1186/s13059-023-02988-9)
Supplement: Supplementary file 1 — Additional file 1: Fig. S1. Inferred vector field of cells during granule cell differentiation in the mouse dentate gyrus using scTour and scVelo. Fig. S2. Application of scTour to the developing mouse hippocampus dataset. Fig. S3. scTour’s inference is robust to cell subsampling. Fig. S4. scTour captures the developmental cellular dynamics in embryonic organoids. Fig. S5. scTour captures the developmental cellular dynamics in the human thymic epithelial cells. Fig. S6. scTour captures the developmental cellular dynamics in human gastrulation. Fig. S7. scTour captures the developmental cellular dynamics in human preimplantation. Fig. S8. scTour captures the cellular dynamics during iPSC reprogramming in mice. Fig. S9. scTour captures the cellular dynamics during iPSC reprogramming in humans. Fig. S10. scTour captures the developmental cellular dynamics during hematopoiesis. Fig. S11. scTour captures the anatomical topography of brain endothelial cells. Fig. S12. Superiority of scTour’s pseudotime over scVelo’s latent time in characterizing a discontinued process. Fig. S13. Comparisons of scTour’s vector field with velocities from other methods. Fig. S14. scTour predicts the cellular dynamics of unseen cellular states regardless of their positions along the developmental process. Fig. S15. scTour reconstructs the transcriptomic space at different developmental stages. Fig. S16. scTour predicts the latent space of unseen datasets. Fig. S17. Benchmarking scTour against existing methods using the dataset profiling mouse gastrulation and early organogenesis. Fig. S18. Application of scTour to scATAC-seq data. Fig. S19. Delineation of the cycling process by scTour’s vector field and RNA velocity. Fig. S20. Application of scTour to terminally differentiated cells. Fig. S21. Assessment of the parameter alpha in the objective function in a dataset of granule cell differentiation. Fig. S22. Assessment of the parameter alpha in the objective function in a dataset of pyramidal [file 13059_2023_2988_MOESM1_ESM.pdf]

Supplemental Figures

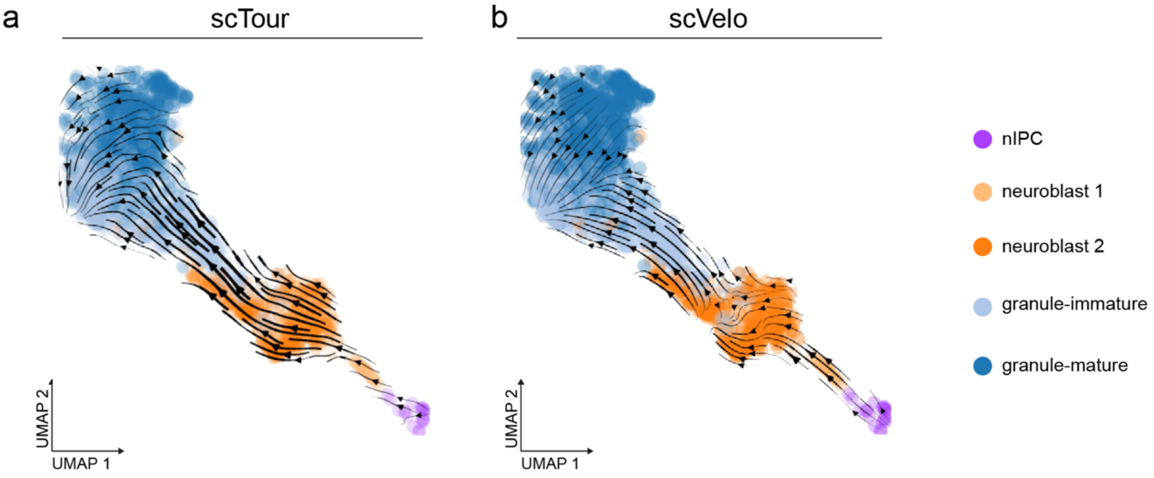

Fig. S1 Inferred vector field of cells during granule cell differentiation in the mouse dentate gyrus using scTour and scVelo. **a, b** UMAP visualizations of the transcriptomic vector field inferred by scTour (**a**) and the RNA velocity estimated by scVelo (**b**) using the cells from the granule cell lineage. Both are inferred based on the cells from the two time points P12 and P35 as in the scVelo publication.

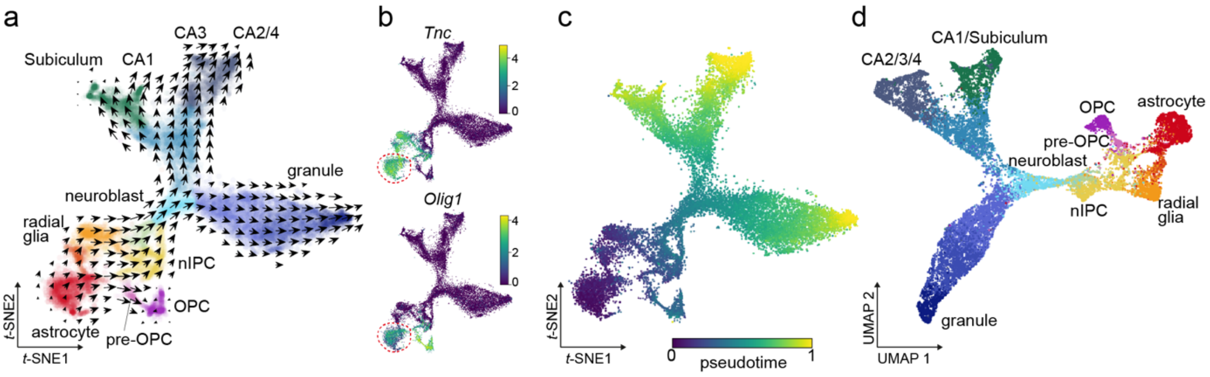

Fig. S2 Application of scTour to the developing mouse hippocampus dataset. **a** *t*-SNE plot of scTour's vector field with colours indicating the cell types. OPC, oligodendrocyte precursors. **b** *t*-SNE plot of the expression of glioblast marker *Tnc* (top) and OPC marker *Olig1* (bottom). Red dotted circle marks the astrocytes. The expression of *Tnc* and *Olig1* in the astrocytes indicates the possible glioblast state of these astrocytes, which probably results in the directional flow from the astrocytes to pre-OPCs as shown in **a**. **c** *t*-SNE plot of the pseudotime estimated by scTour. **d** UMAP visualization of the latent space derived from scTour with colours denoting the cell types as in **a**.

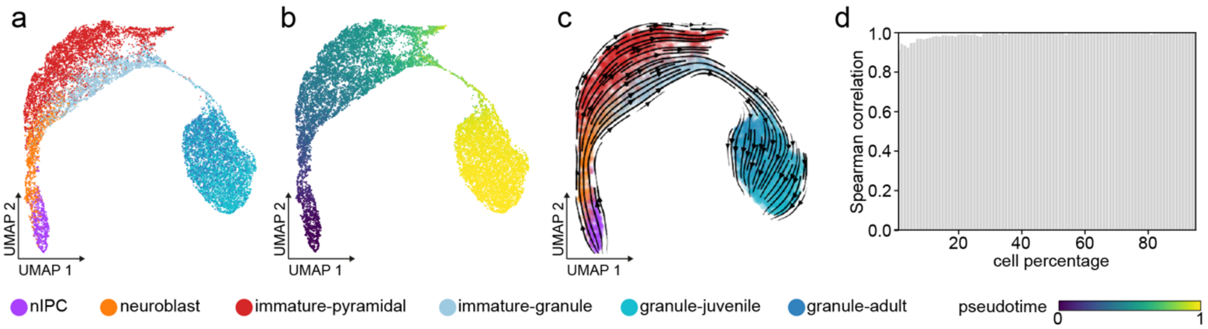

Fig. S3 scTour's inference is robust to cell subsampling. **a-c** UMAP visualizations of the latent space (**a,** coloured by cell types), developmental pseudotime (**b**), and transcriptomic vector field (**c**) inferred from the scTour model trained using 1% of cells from the pyramidal neuron and granule cell lineages. **d** The Spearman correlation coefficient calculated between the pseudotime estimated from the model trained using 95% of total cells and those from models trained with cell subsets (1% to 95% from left to right).

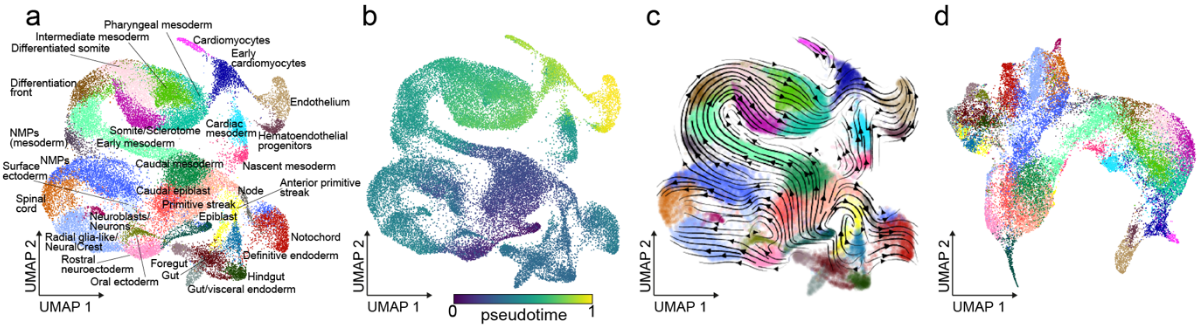

Fig. S4 scTour captures the developmental cellular dynamics in embryonic organoids. **a** UMAP visualization of the cell types from 30,496 cells collected from mouse gastruloids. The UMAP coordinates in **a-c** and cell type annotations are drawn from the original study. NMPs, neuromesodermal progenitors. **b-d** UMAP visualizations of the inferred developmental pseudotime (**b**), transcriptomic vector field (**c**), and latent space (**d**) from the scTour model trained using 60% of all cells.

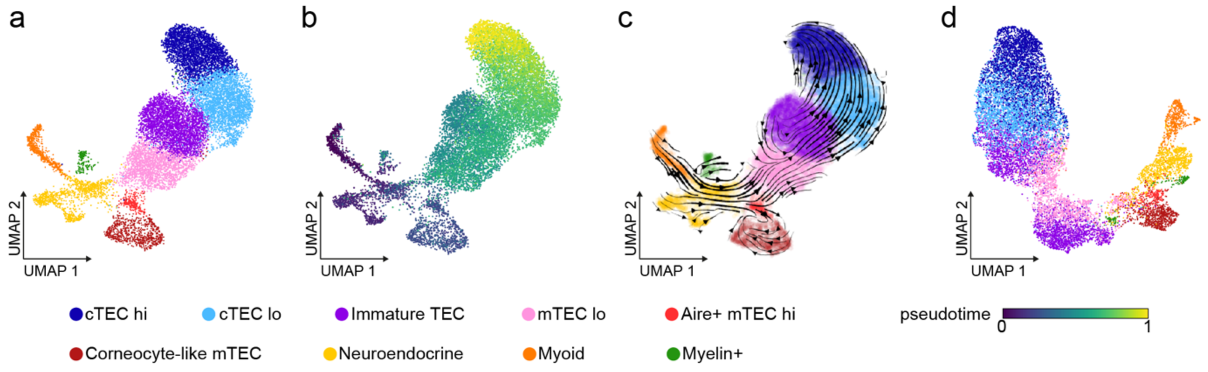

Fig. S5 scTour captures the developmental cellular dynamics in the human thymic epithelial cells. **a** UMAP visualization of the cell types from 14,217 cells collected from the human thymic epithelial compartment at different stages. The UMAP coordinates in **a-c** and cell type annotations are from the original study. TEC, thymic epithelial cells; cTEC, cortical TEC; mTEC, medullary TEC. **b-d** UMAP visualizations of the inferred developmental pseudotime (**b**), transcriptomic vector field (**c**), and latent space (**d**) from the scTour model trained using 60% of all cells.

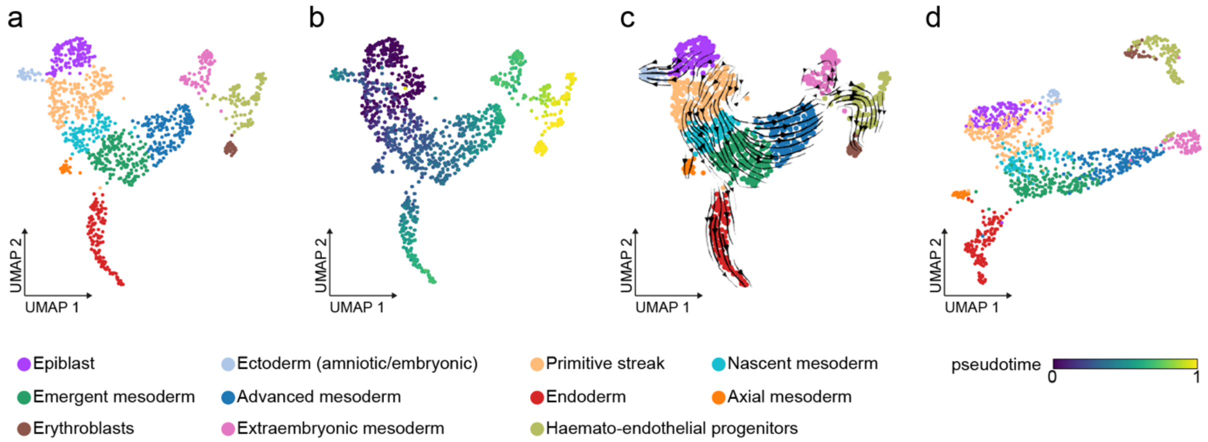

Fig. S6 scTour captures the developmental cellular dynamics in human gastrulation. **a** UMAP visualization of the cell types from 1,195 cells collected from a gastrulating human embryo. The UMAP coordinates in **a-c** and cell type annotations are from the original study. **b-d** UMAP visualizations of the inferred developmental pseudotime (**b**), transcriptomic vector field (**c**), and latent space (**d**) from the scTour model trained using 90% of all cells.

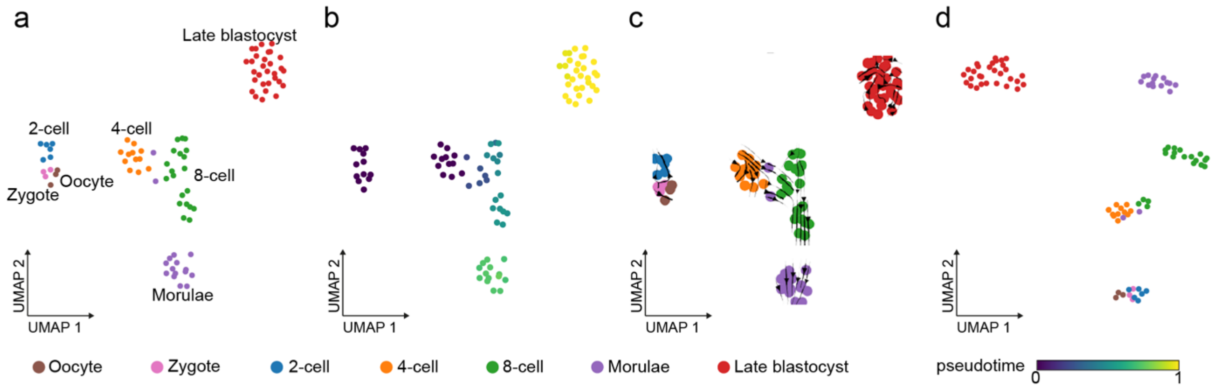

Fig. S7 scTour captures the developmental cellular dynamics in human preimplantation. **a** PCA space-based UMAP visualization of the 90 cells collected from human preimplantation embryos. **b-d** UMAP visualizations of the inferred developmental pseudotime (**b**), transcriptomic vector field (**c**), and latent space (**d**) from the scTour model trained using 90% of all cells.

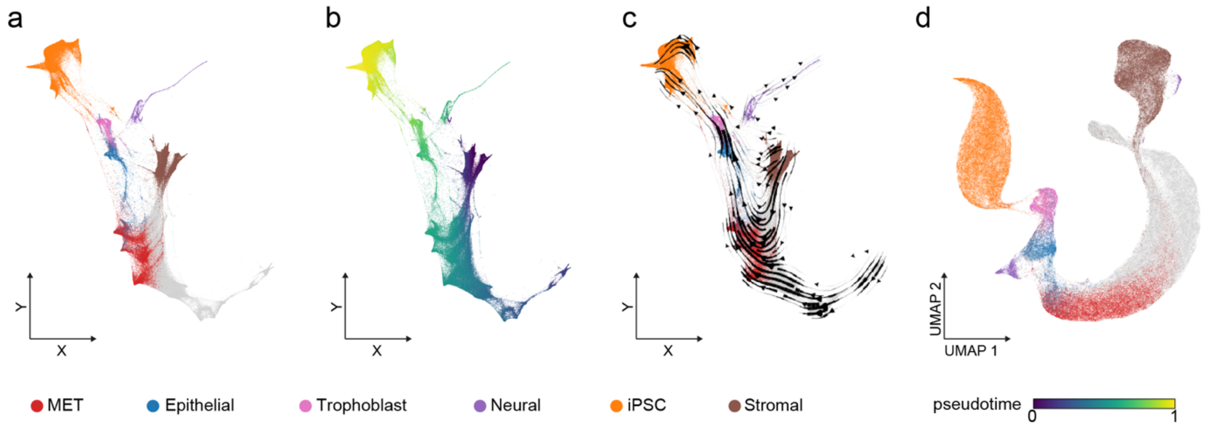

Fig. S8 scTour captures the cellular dynamics during iPSC reprogramming in mice. **a** Force-directed layout embedding (FLE) visualizing cell states from 251,203 cells collected during iPSC reprogramming. The FLE coordinates in **a-c** and cell annotations are from the original study. MET, mesenchymal-to-epithelial transition. **b-d** FLE (**b-c**) and UMAP (**d**) visualizations of the inferred developmental pseudotime (**b**), transcriptomic vector field (**c**), and latent space (**d**) from the scTour model trained using 20% of all cells.

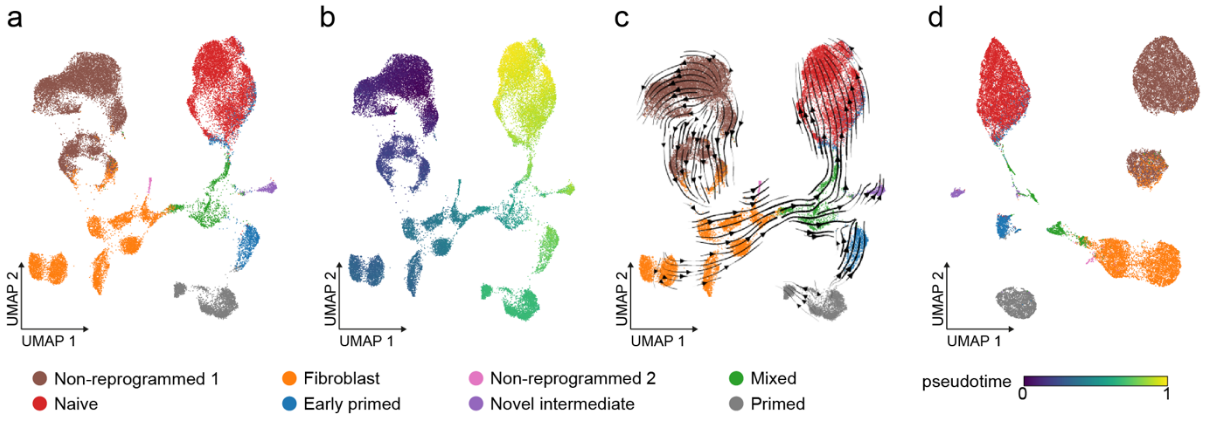

Fig. S9 scTour captures the cellular dynamics during iPSC reprogramming in humans. **a** UMAP visualization of the cell states from 36,597 nuclei collected during iPSC reprogramming and measured by snRNA-seq. The UMAP coordinates in **a-c** and cell annotations are from the original study. **b-d** UMAP visualizations of the inferred developmental pseudotime (**b**), transcriptomic vector field (**c**), and latent space (**d**) from the scTour model trained using 60% of all cells.

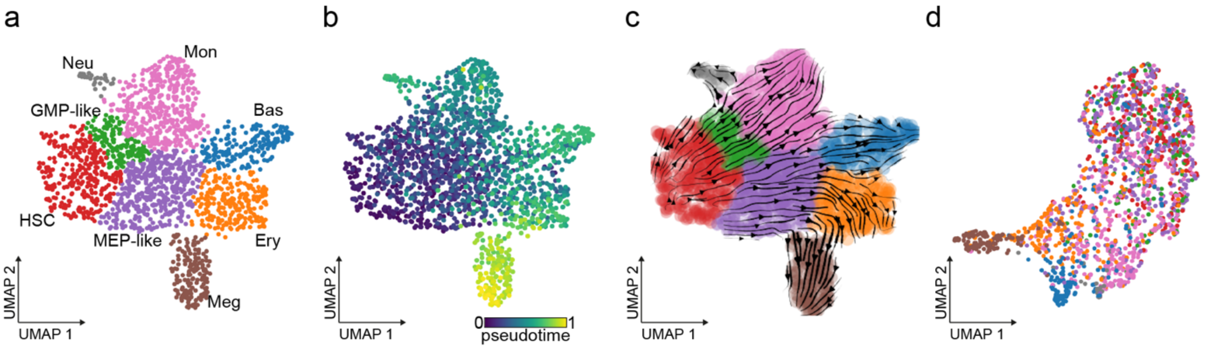

Fig. S10 scTour captures the developmental cellular dynamics during hematopoiesis. **a** UMAP visualization of the cell lineages from 1,947 cells profiled by scNT-seq during hematopoiesis. The UMAP coordinates in **a-c** and cell type annotations are from the original study. HSC, hematopoietic stem cell; Meg, megakaryocyte; Ery, erythrocyte; Bas, basophil; Mon, monocyte; Neu, neutrophil; GMP-like, granulocyte and monocyte progenitor-like cells; MEP-like, Meg and Ery progenitor-like cells. **b-d,** UMAP visualizations of the inferred developmental pseudotime (**b**), transcriptomic vector field (**c**), and latent space (**d**) from the scTour model trained using 90% of all cells.

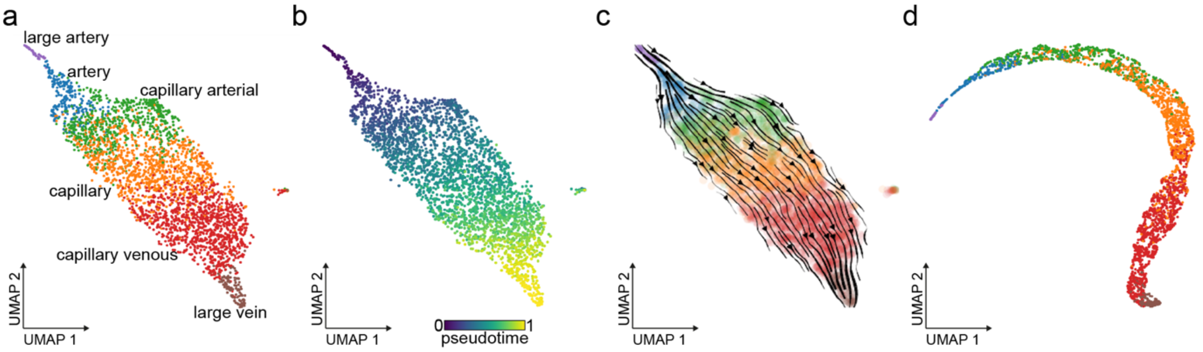

Fig. S11 scTour captures the anatomical topography of brain endothelial cells. **a** PCA space-based UMAP visualization of the cell clusters from 3,105 endothelial cells collected from the mouse brain. The cell type annotations are from the original study. **b-d** UMAP visualizations of the inferred developmental pseudotime (**b**), transcriptomic vector field (**c**), and latent space (**d**) from the scTour model trained using 90% of all cells.

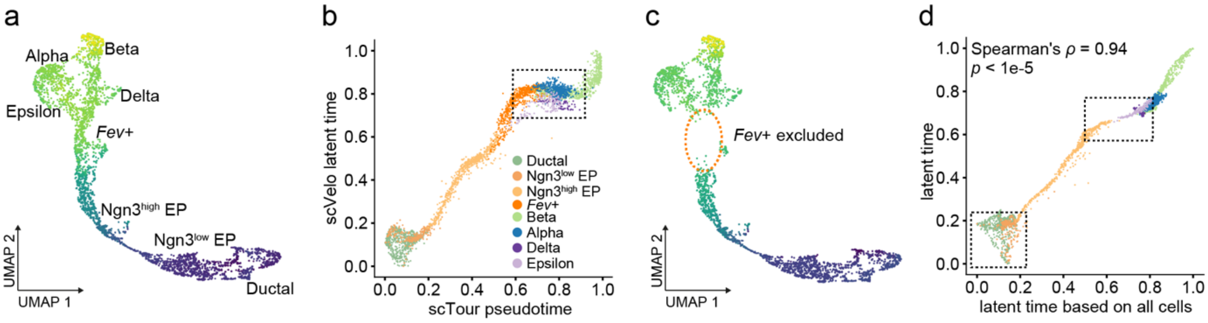

Fig. S12 Superiority of scTour's pseudotime over scVelo's latent time in characterizing a discontinued process. **a** UMAP showing the latent time inferred by scVelo for all the cells along the endocrinogenesis process. **b** Comparison of scTour's pseudotime (x axis) with scVelo's latent time (y axis). Dots represent cells coloured by cell identities. The rectangle marks the continuous differentiation process from *Fev*+ endocrine cells to terminal cell fates captured by scTour's pseudotime but not by scVelo's latent time. **c** UMAP showing the latent time inferred by scVelo when *Fev*+ endocrine cells are excluded. **d** Comparison of the latent time from **c** (inferred from cell subset with *Fev*+ endocrine cells held out, y axis) with that from **a** (inferred from all cells, x axis). The bottom-left rectangle shows the early window of progenitor cells which is not properly delineated by scVelo's latent time when *Fev*+ endocrine cells are held out. The rectangle on top marks the inability of scVelo to capture the transcriptomic discontinuity between $\text{Ngn3}^{\text{high}}$ EPs and terminally differentiated cells when the intermediate *Fev*+ endocrine cells are excluded.

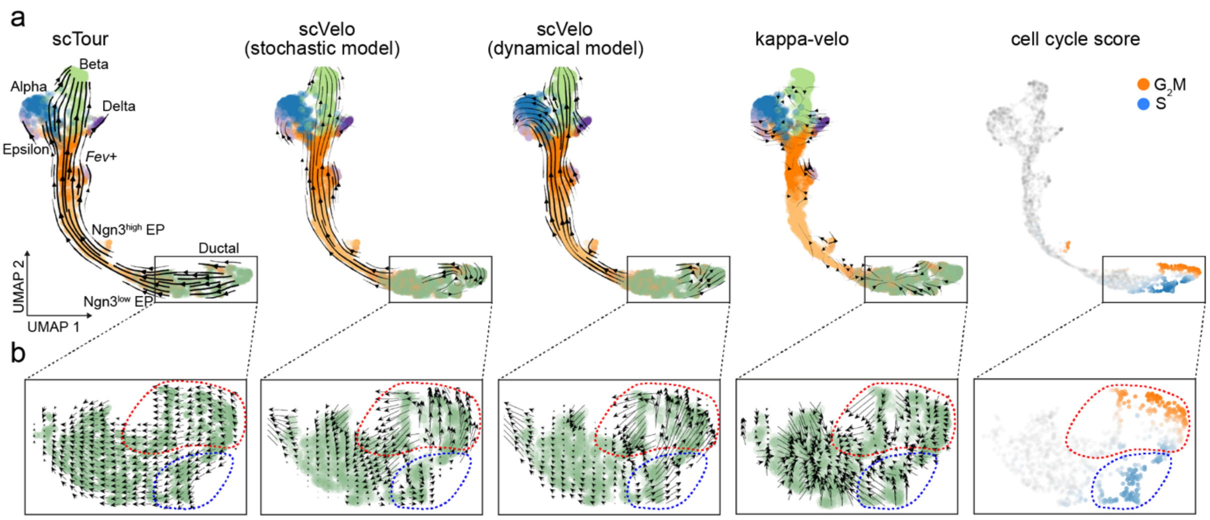

Fig. S13 Comparisons of scTour's vector field with velocities from other methods. **a** UMAP visualizations of scTour's vector field (left), the RNA velocity estimated from scVelo's stochastic and dynamical models (middle), the velocity derived from κ-velo (the fourth panel), and the cell cycle scores (right) based on the dataset of pancreatic endocrinogenesis. **b** Zoom-in view of the velocity estimates of the cycling cells. Blue and red dotted circles highlight the S-G_2M transition and exit of the cell cycle, respectively.

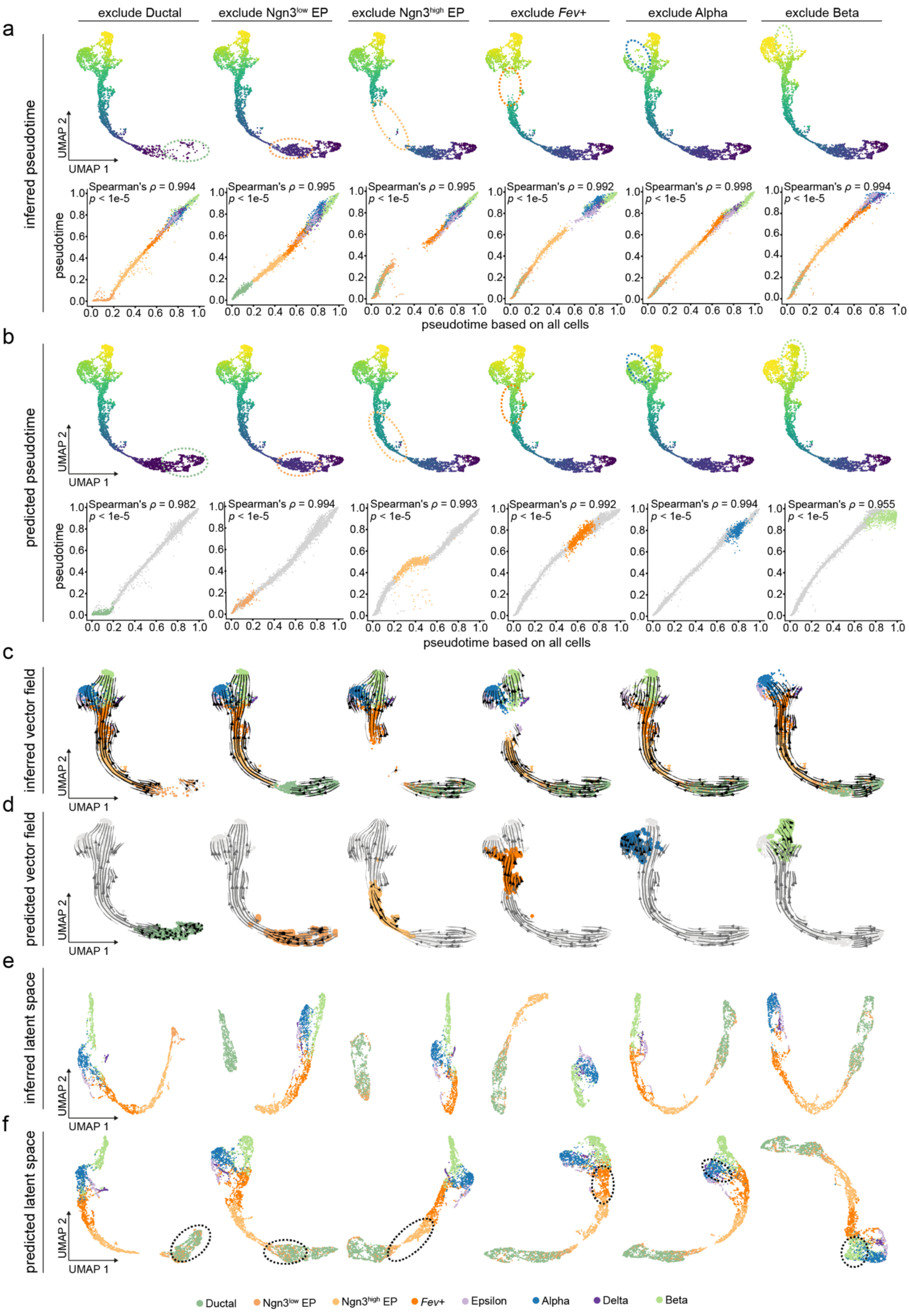
exclude Ductal
exclude Ngn3low EP
exclude Ngn3high EP
exclude Fev+
exclude Alpha
exclude Beta
a
inferred pseudotime
UMAP 2
UMAP 1
pseudotime
Spearman's ρ = 0.994
p < 1e-5
Spearman's ρ = 0.995
p < 1e-5
Spearman's ρ = 0.995
p < 1e-5
Spearman's ρ = 0.992
p < 1e-5
Spearman's ρ = 0.998
p < 1e-5
Spearman's ρ = 0.994
p < 1e-5
pseudotime based on all cells
b
predicted pseudotime
Spearman's ρ = 0.982
p < 1e-5
Spearman's ρ = 0.994
p < 1e-5
Spearman's ρ = 0.993
p < 1e-5
Spearman's ρ = 0.992
p < 1e-5
Spearman's ρ = 0.994
p < 1e-5
Spearman's ρ = 0.955
p < 1e-5
pseudotime based on all cells
c
inferred vector field
d
predicted vector field
e
inferred latent space
f
predicted latent space
Ductal
Ngn3low EP
Ngn3high EP
Fev+
Epsilon
Alpha
Delta
Beta

Fig. S14 scTour predicts the cellular dynamics of unseen cellular states regardless of their positions along the developmental process. **a** UMAP visualizations of the developmental pseudotime inferred by the scTour model trained with a certain cellular state held out (top panels). Scatter plots in the bottom panels show the comparisons with the pseudotime estimates from the model trained with all cells. The Spearman correlation coefficients and *p*-values calculated between the two sets of estimates are shown on top. **b** Top panel: UMAP visualizations showing the predicted pseudotime for the held-out cellular states (dotted circles). Bottom panel: scatter plot showing the comparisons of the prediction with the ground truth. The Spearman correlation coefficients and *p*-values calculated between pseudotime in x and y axes are shown on top. **c** UMAP representations of the transcriptomic vector fields inferred by the scTour model trained with a certain cellular state held out. **d** UMAP representations of the predicted transcriptomic vector fields for the held-out cellular states. **e** UMAP visualizations of the latent representation derived from the scTour model trained with a certain cellular state held out. **f** UMAP visualizations based on the predicted latent representations for the held-out cellular states (dotted circles) and those inferred from the training cells.

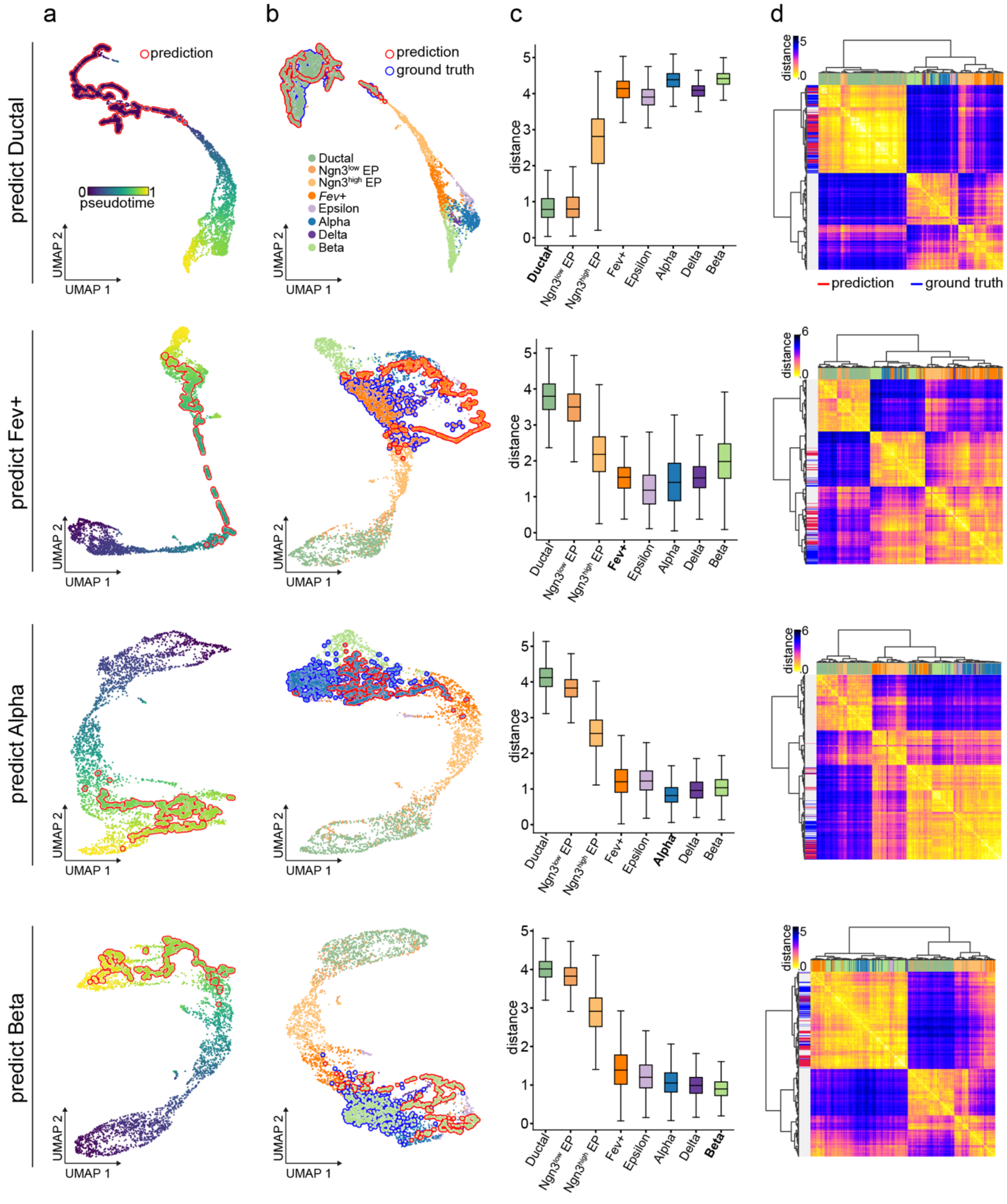

Fig. S15 scTour reconstructs the transcriptomic space at different developmental stages. **a** UMAP visualizations of the reconstructed latent representations for the held-out cellular states given their expected developmental pseudotime (red outline) and the latent space inferred from the training cells. **b** UMAP visualizations based on the latent representations from the reconstructed cells (red outline), ground-truth cells (blue outline), and remaining cells. Cells are colour-coded by cell types. **c** Box plots showing the Euclidean distances calculated between the reconstructed latent representations of the held-out cellular state and those from the ground

truth and remaining cellular states, with the medians, interquantile ranges, and 5th, 95th percentiles indicated by centre lines, hinges, and whiskers, respectively. **d** Unsupervised hierarchical clustering of the reconstructed cells along with all the other cells based on their Euclidean distances in the scTour latent space. Column colours of the heatmap mark the cell types and row colours denote the reconstructed (red), ground-truth (blue), and remaining (grey) cells. The colour shades of the heatmap indicates the Euclidean distance.

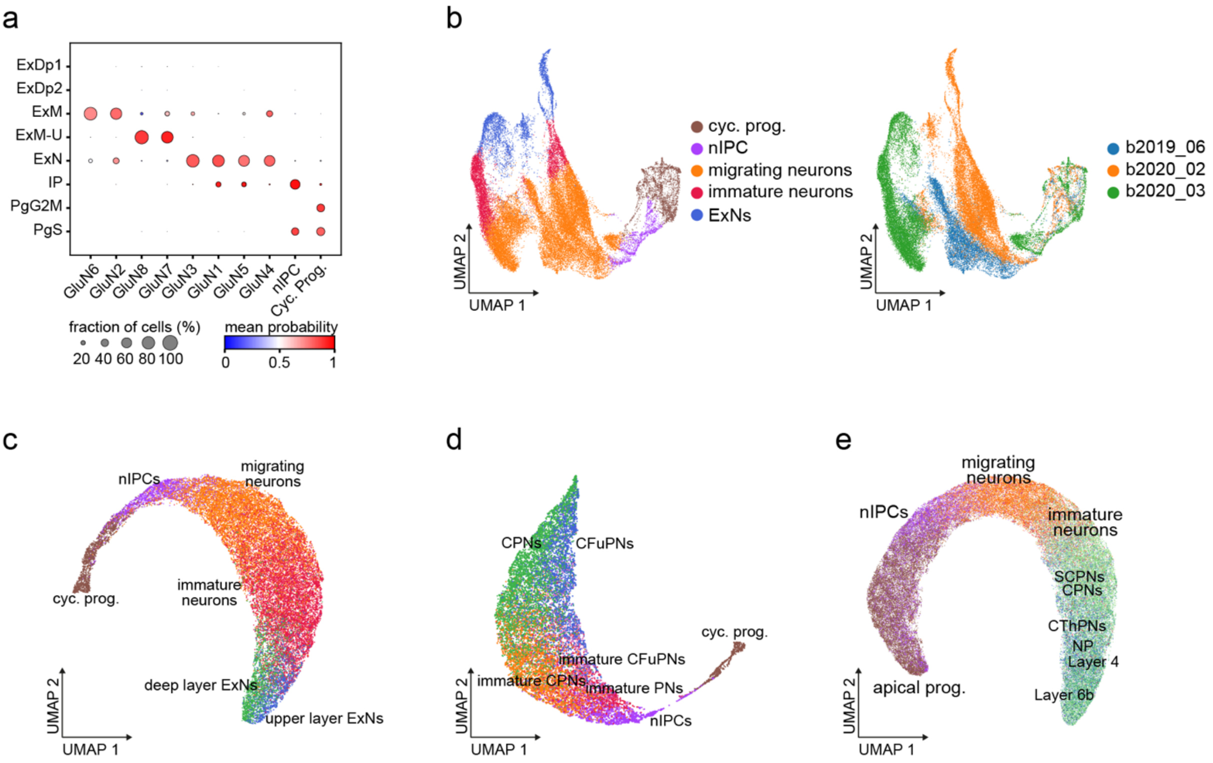

Fig. S16 scTour predicts the latent space of unseen datasets. **a** Dot plot showing the cell type label transfer from Polioudakis et al. (row) to cells from Trevino et al. (column). Across rows of each column, dot size indicates the proportion of cells assigned to a given cell type from Polioudakis et al. and colour represents the average probabilities calculated by CellTypist. **b** PCA space-based UMAP visualizations of the cell types from the training dataset as shown in Fig. 4a, with cells coloured by cell types (left) and sample batches (right). **c-e** UMAP visualizations of the predicted latent representations for the three test datasets (Fig. 4b-d): the developing human cortex (**c**), the human brain organoid (**d**), and the developing mouse cortex (**e**). GluN, glutamatergic neuron; ExDp, deep layer excitatory neuron; ExM, maturing excitatory neuron; ExM-U, upper-layer-enriched maturing excitatory neuron; ExN, migrating excitatory neuron; IP, intermediate progenitor; PgG2M, cycling progenitors (G_2/M phase); PgS, cycling progenitors (S phase); cyc. prog., cycling progenitors; nIPCs, neuronal intermediate progenitor cells; ExNs, excitatory neurons; PNs, projection neurons; CPNs, callosal projection neurons; CFuPNs, corticofugal projection neurons; CThPNs, corticothalamic projection neurons; NP, near projecting; SCPNs, subcerebral projection neurons; apical prog., apical progenitors.

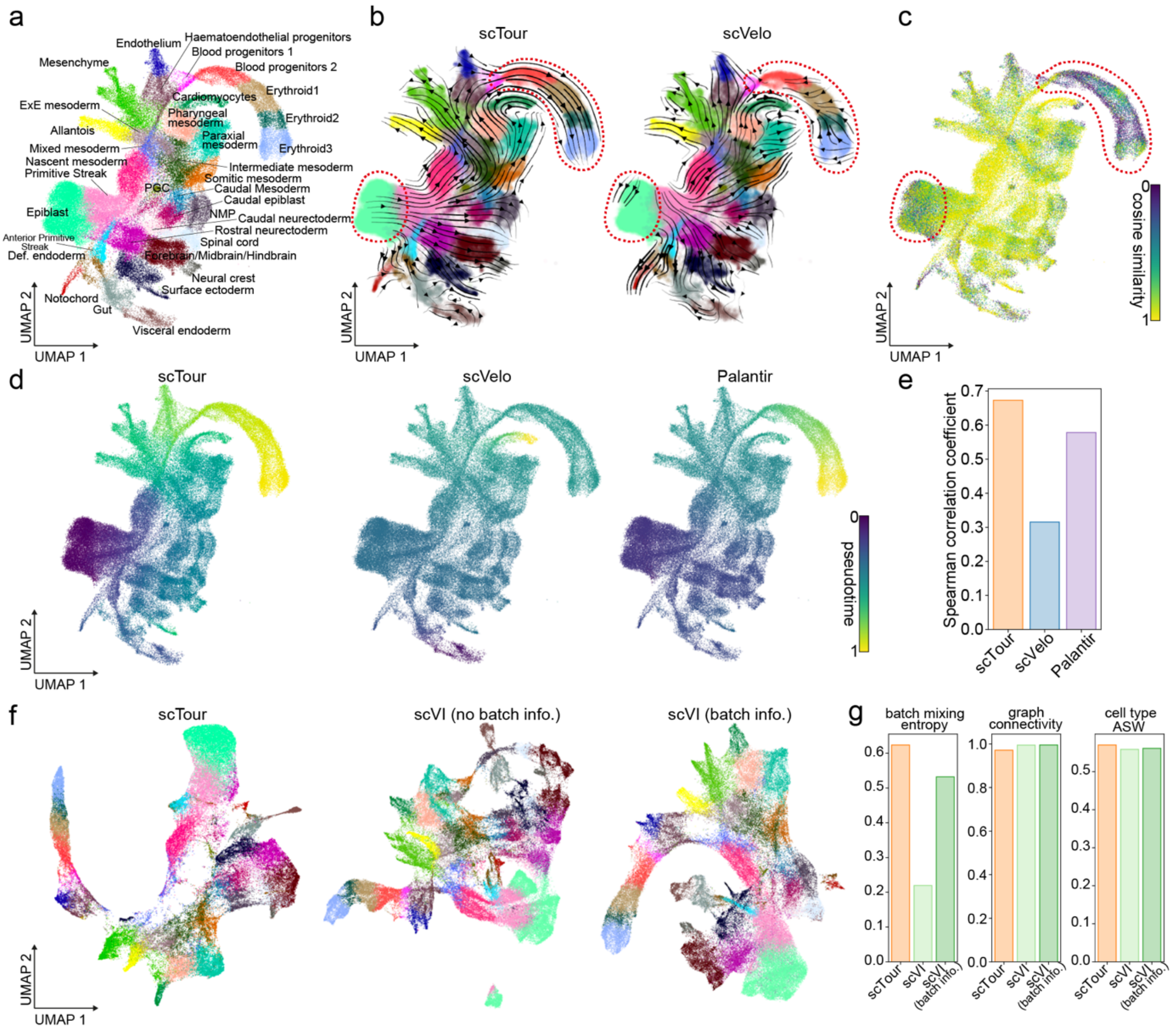

Fig. S17 Benchmarking scTour against existing methods using the dataset profiling mouse gastrulation and early organogenesis. **a** UMAP displaying the cell types identified from the stage of mouse gastrulation and early organogenesis. The UMAP coordinates and cell type annotations are from the original study. PGC, primordial germ cells; NMP, neuromesodermal progenitors; Def., definitive; ExE, extra-embryonic. **b** UMAP visualizations of the vector field inferred from scTour (left) and RNA velocity estimated by scVelo (right). Dotted circles mark the epiblast cells and cells from the erythroid lineage. **c** UMAP visualization of the cosine similarities calculated between the projected velocity from scTour and that from scVelo. **d** UMAP displaying the pseudotime estimated by scTour (left), scVelo (middle), and Palantir (right). **e** The Spearman correlation coefficients calculated between the pseudotime from the three methods shown in **d** and known developmental stages. **f** UMAP visualizations based on the latent space derived from scTour (left), scVI with no batch information provided (middle), and scVI with batch information incorporated (right). **g** Metrics quantifying the degree of batch

correction (batch mixing entropy and graph connectivity) and biological signal conservation (cell type ASW) for each method. ASW, average silhouette width.

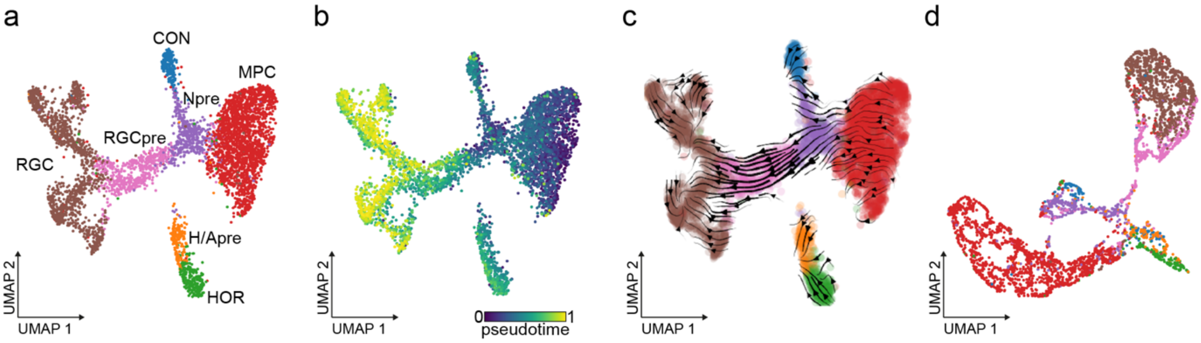

Fig. S18 Application of scTour to scATAC-seq data. **a** Latent semantic indexing (LSI)-based UMAP visualization of the cell types identified from 4,883 cells of the human fetal retina measured by scATAC-seq. RGC, retinal ganglion cells; CON, cones; Npre, neurogenic precursors; RGCpre, RGC precursors; H/Apre, the horizontal/amacrine precursors; MPC, multipotent progenitor cells; HOR, horizontal cells. **b-d** UMAP visualizations of the inferred developmental pseudotime (**b**), epigenetic vector field (**c**), and latent space (**d**) from the scTour model trained using 90% of cells and top 25% of peaks (34,670 genomic regions).

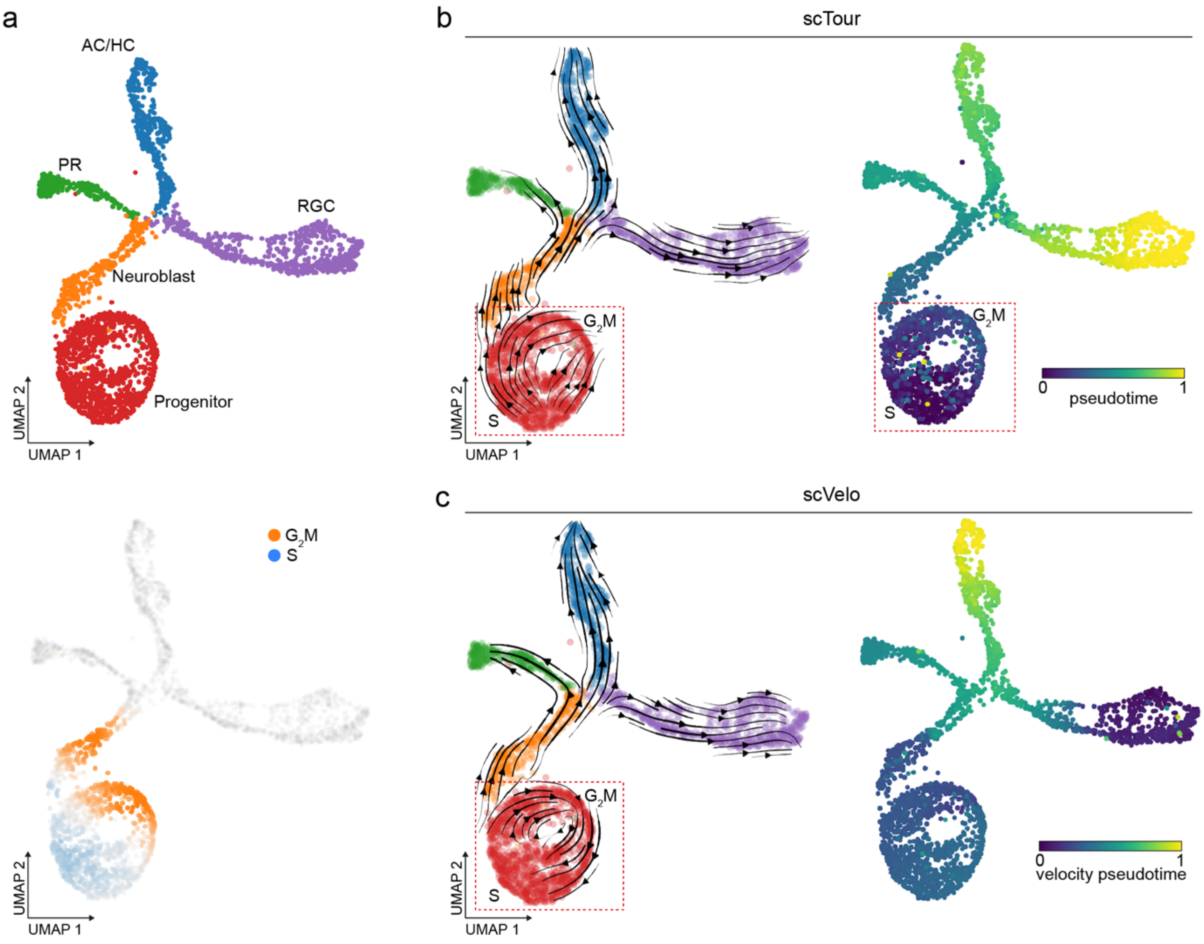

Fig. S19 Delineation of the cycling process by scTour's vector field and RNA velocity. **a** UMAP visualizations of the cell types from developing mouse retina (top) and the cell cycle scores (bottom). PR, photoreceptors; AC/HC, amacrine/horizontal cells; RGC, retinal ganglion cells. **b** UMAP visualizations of the vector field and pseudotime estimated by scTour. Dotted circle highlights the cycling cells. **c** As in **b**, but with the velocity and pseudotime estimated by scVelo.

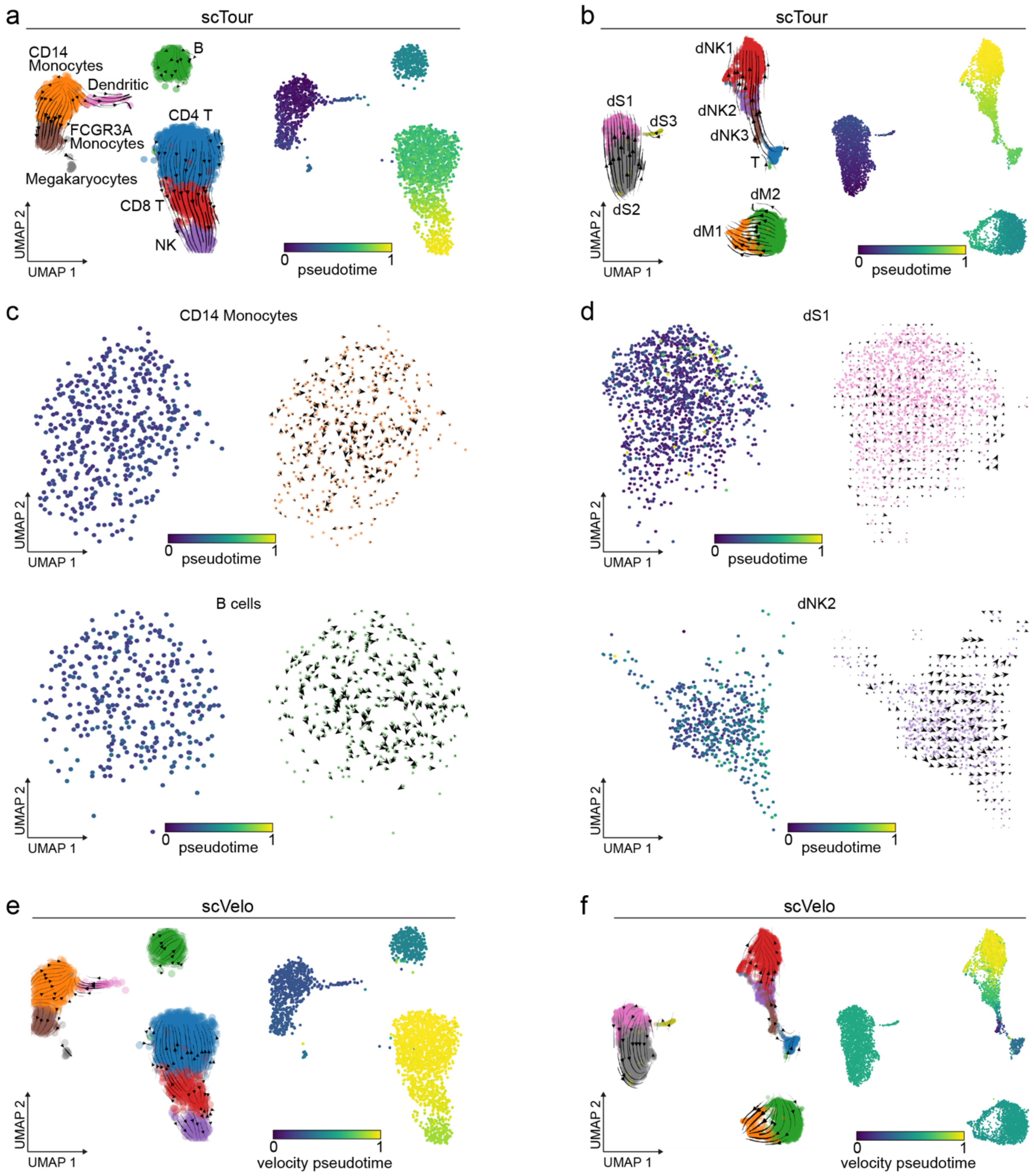

Fig. S20 Application of scTour to terminally differentiated cells. **a, b** UMAP visualizations of the transcriptomic vector field and developmental pseudotime inferred from the scTour model trained based on the PBMC 3k dataset (**a**, from 10x Genomics) and human decidual dataset (**b**, Vento-Tormo et al., 2018). dS, decidual stromal cells; dNK, decidual natural killer cells; dM, decidual macrophages. **c, d** UMAP visualizations of the pseudotime and vector field derived from the scTour model trained based only on CD14 monocytes (**c**, top), B cells (**c**, bottom),

and dS1 (**d**, top) and dNK2 (**d**, bottom). **e, f** UMAP visualizations of the RNA velocity and velocity pseudotime estimated by scVelo for the PBMC 3k (**e**) and human decidual (**f**) datasets.

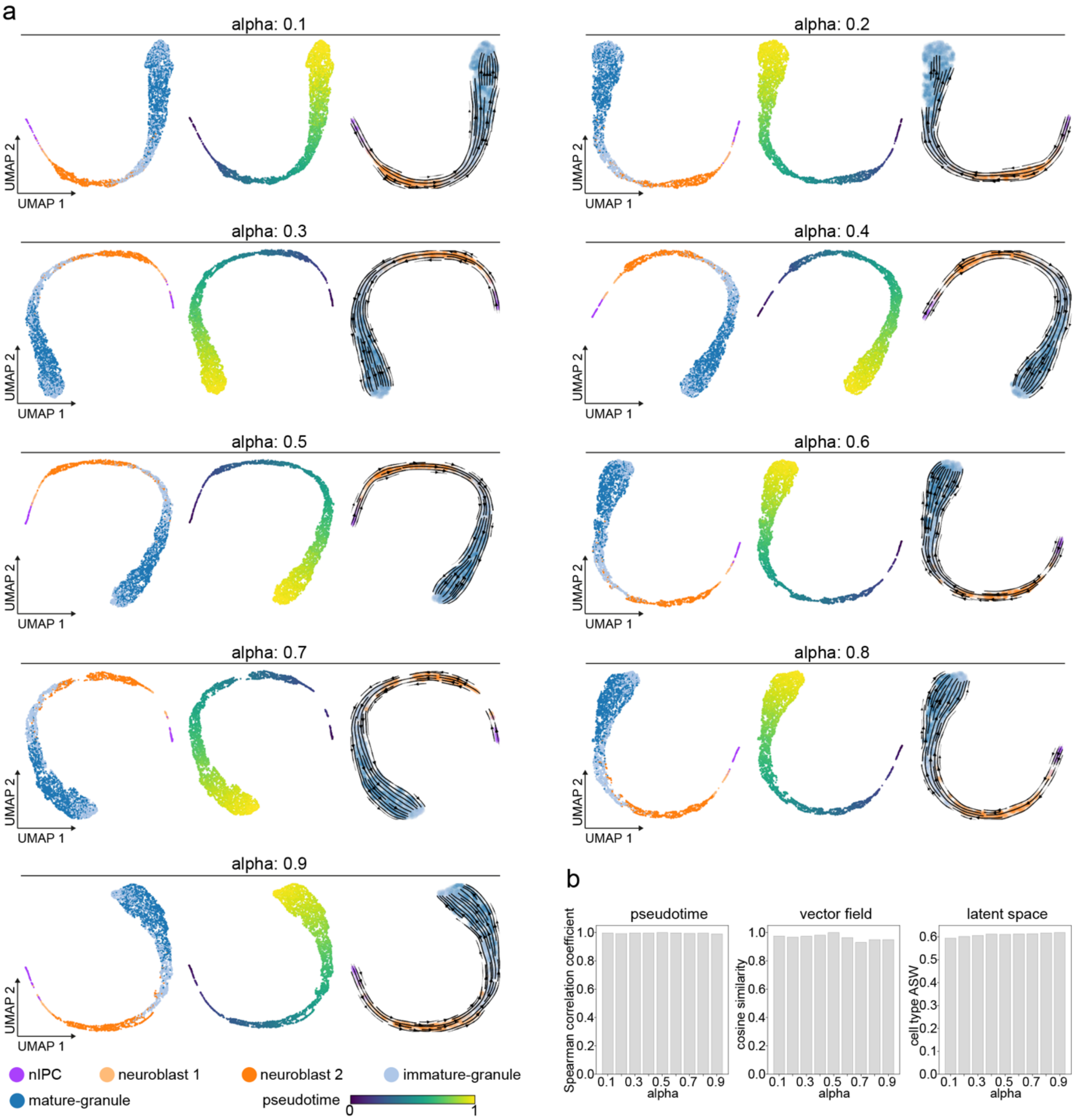

Fig. S21 Assessment of the parameter alpha in the objective function in a dataset of granule cell differentiation. **a** UMAP visualizations of the latent representations (left, coloured by cell types), pseudotime (middle), and vector field (right) inferred from the scTour models trained with a series of alpha values (the alpha in the objective function). **b** The Spearman correlation coefficients calculated between the pseudotime estimated from the model with alpha set to 0.5 (the default setting) and those from the models with other alpha settings (left), the cosine similarities computed between the projected velocities from the model with the default alpha (0.5) and those from the other models (middle), and the cell type ASW measuring the biological signal conservation under the latent space inferred from different alpha settings (right). ASW, average silhouette width.

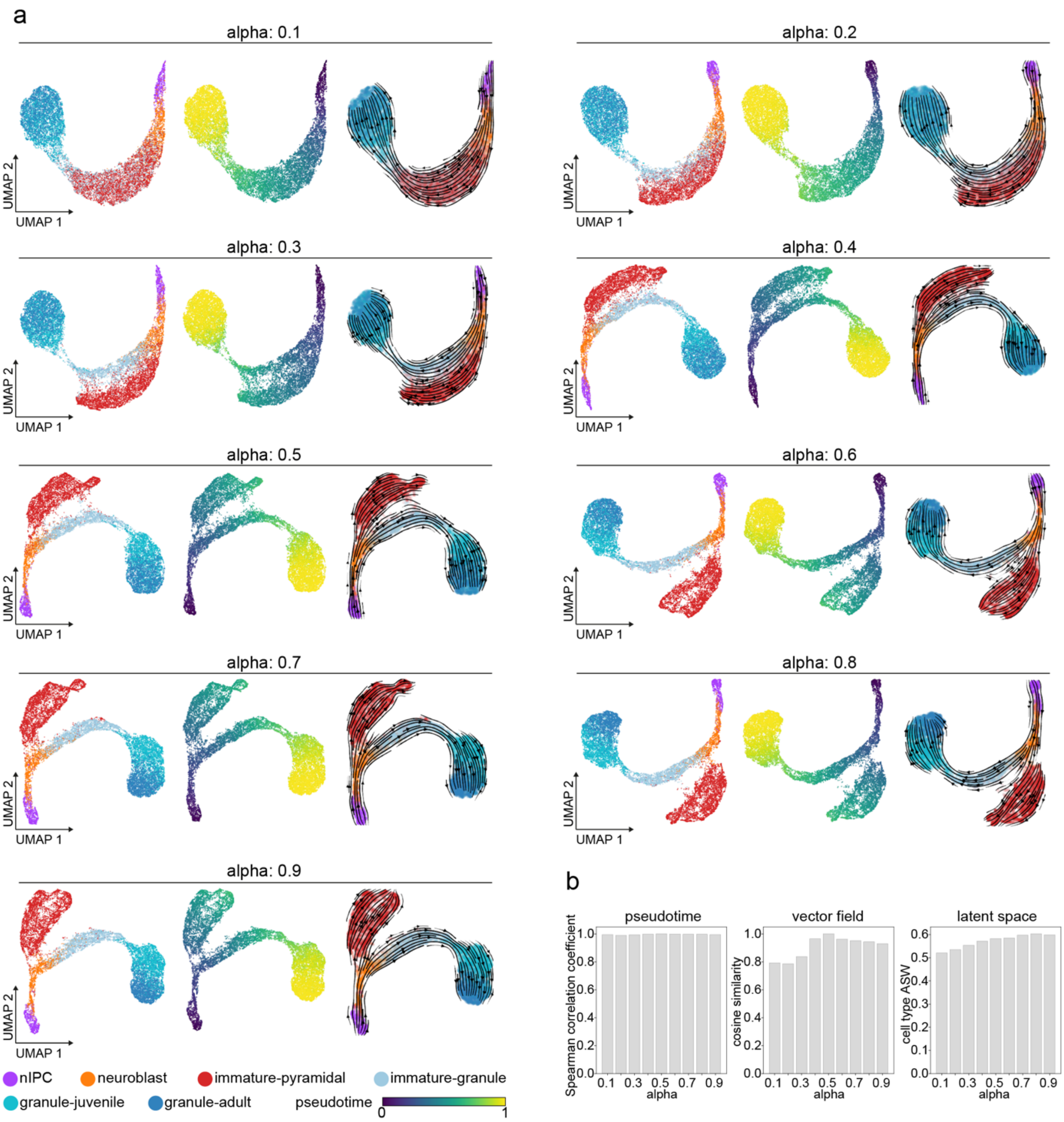

Fig. S22 Assessment of the parameter alpha in the objective function in a dataset of pyramidal neuron and granule cell development. **a** UMAP visualizations of the latent representations (left, coloured by cell types), pseudotime (middle), and vector field (right) inferred from the scTour models trained with a series of alpha values (the alpha in the objective function). **b** The Spearman correlation coefficients calculated between the pseudotime estimated from the model with alpha set to 0.5 (the default setting) and those from the models with other alpha settings (left), the cosine similarities computed between the projected velocities from the model with the default alpha (0.5) and those from the other models (middle), and the cell type ASW measuring the biological signal conservation under the latent space inferred from different alpha settings (right). ASW, average silhouette width.

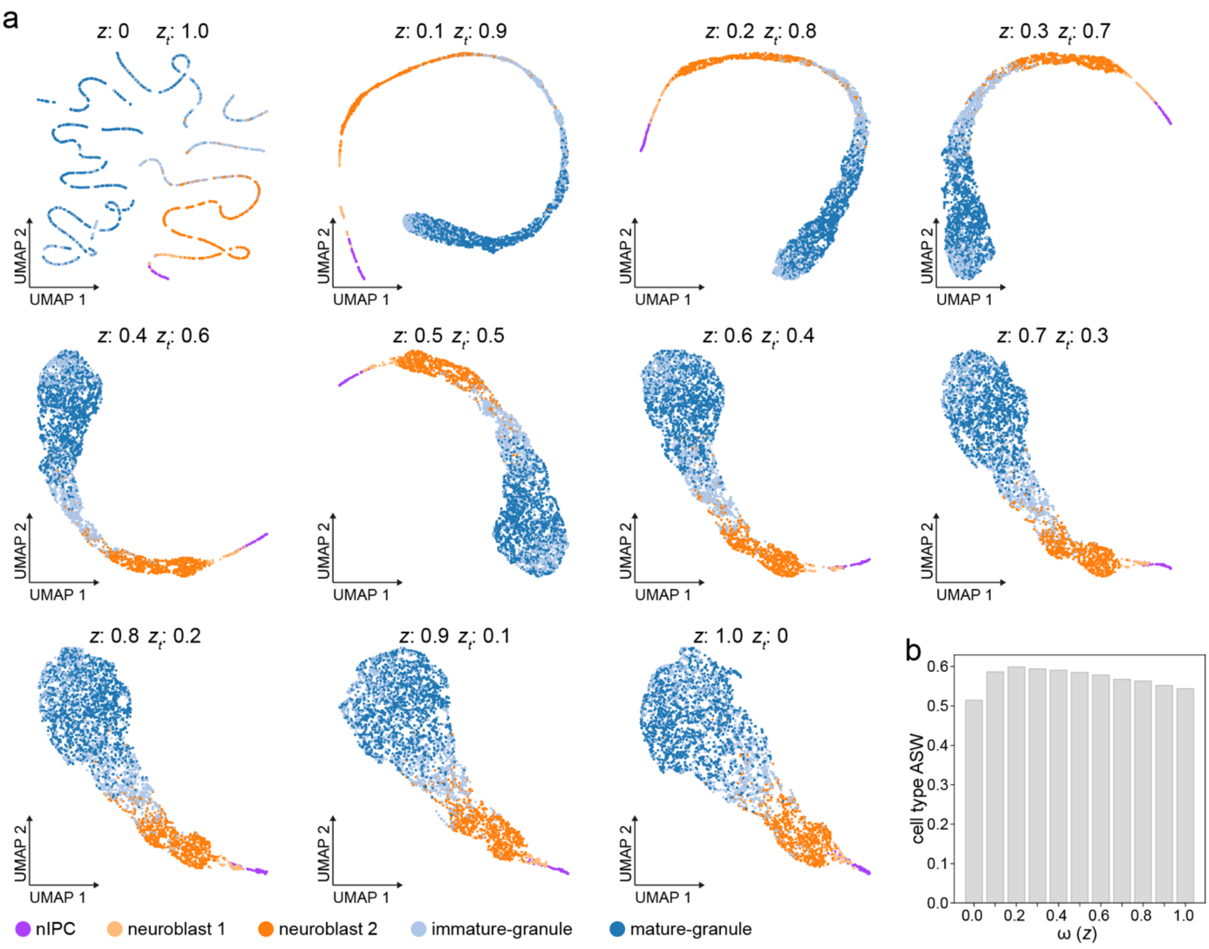

Fig. S23 Assessment of contributions of z and z_t to scTour's latent representation in a dataset of granule cell development. **a** UMAP visualizations of the latent representations derived from varying combinations of z (from the variational inference) and z_t (from the ODE solver) inferred based on the dataset of granule cell development in the mouse dentate gyrus. Colours of cells indicate cell types. **b** Cell type average silhouette width (ASW) quantifying the biological signal conservation under different latent representations shown in **a**.

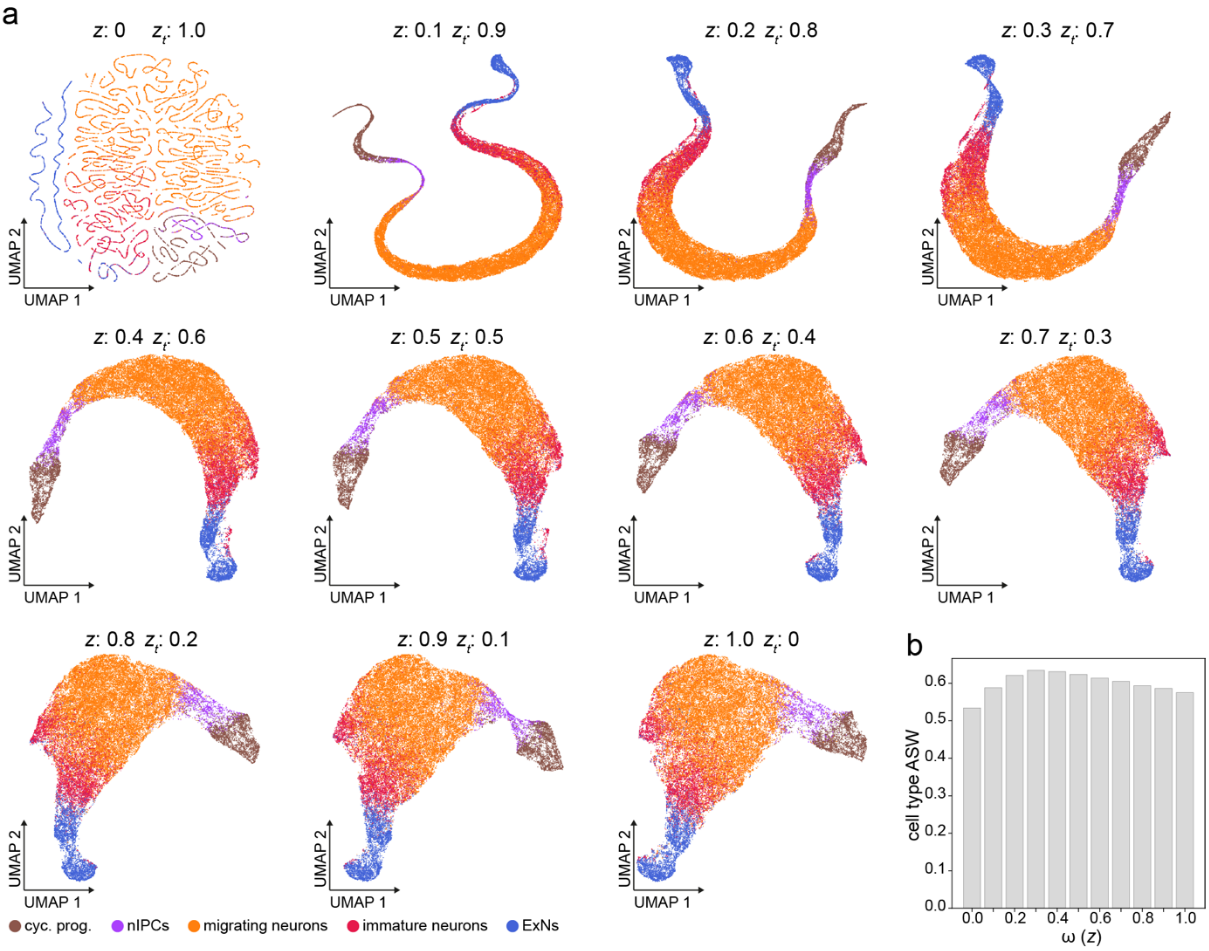

Fig. S24 Assessment of contributions of z and z_t to scTour's latent representation in a dataset of excitatory neuron development. **a** UMAP visualizations of the latent representations derived from varying combinations of z (from the variational inference) and z_t (from the ODE solver) inferred based on the dataset of excitatory neuron development in the human cortex. Colours of cells indicate cell types. cyc. prog., cycling progenitors; nIPCs, neuronal intermediate progenitor cells; ExNs, excitatory neurons. **b** Cell type average silhouette width (ASW) quantifying the biological signal conservation under different latent representations shown in **a**.

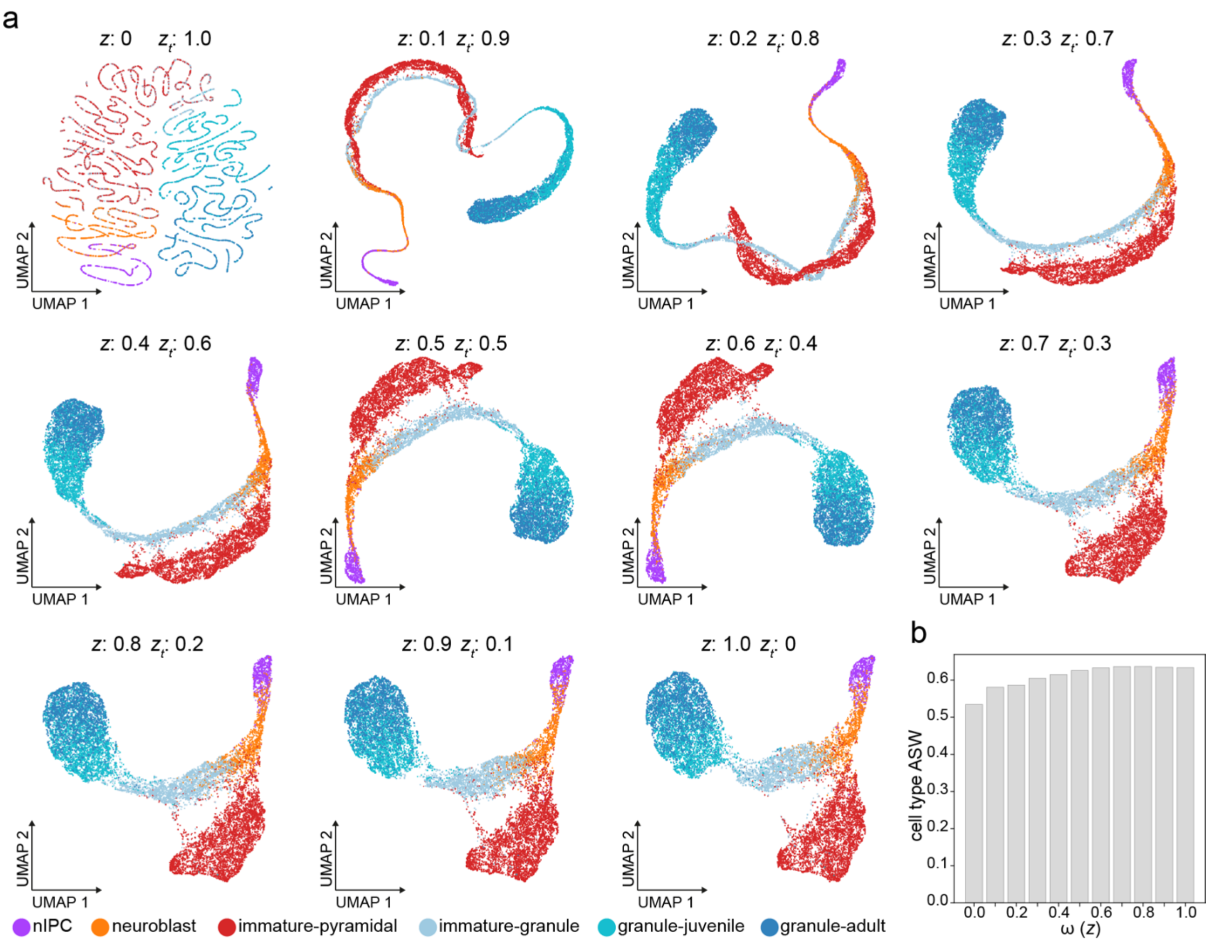

Fig. S25 Assessment of contributions of z and z_t to scTour's latent representation in a dataset of granule cell and pyramidal neuron development. **a** UMAP visualizations of the latent representations derived from varying combinations of z (from the variational inference) and z_t (from the ODE solver) inferred based on the dataset of granule cell and pyramidal neuron development in the mouse hippocampus. Colours of cells indicate cell types. **b** Cell type average silhouette width (ASW) quantifying the biological signal conservation under different latent representations shown in **a**.

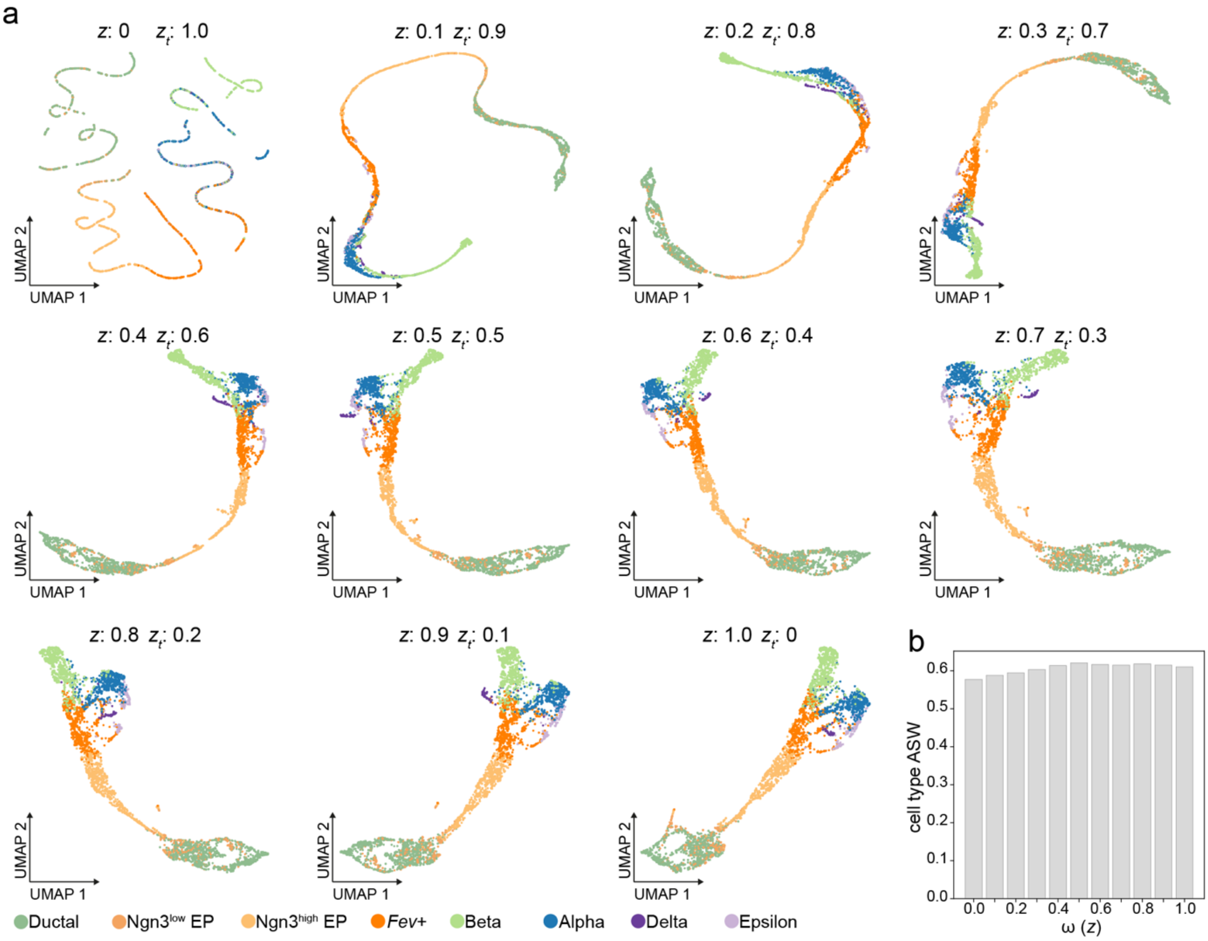

Fig. S26 Assessment of contributions of z and z_t to scTour's latent representation in a dataset of pancreatic endocrinogenesis. **a** UMAP visualizations of the latent representations derived from varying combinations of z (from the variational inference) and z_t (from the ODE solver) inferred based on the dataset of endocrinogenesis in the mouse pancreas. Colours of cells indicate cell types. **b** Cell type average silhouette width (ASW) quantifying the biological signal conservation under different latent representations shown in **a**.
