# Supplementary material for: scTour: a deep learning architecture for robust inference and accurate prediction of cellular dynamics
Source: Genome Biol. 2023 Jun 23;24:149. doi: 10.1186/s13059-023-02988-9 (PMC10290357; doi:10.1186/s13059-023-02988-9)
Supplement: Supplementary file 5 — Additional file 5. Review history. [file 13059_2023_2988_MOESM5_ESM.docx]

Review History

**First round of review**

**Reviewer 1**

**Were you able to assess all statistics in the manuscript, including the appropriateness of statistical tests used?**

No

**Were you able to directly test the methods?**

No

**Comments to author:**

The author presents an innovative approach to perform pseudotime estimation and in turn use this estimate to estimate RNA velocity by means of an twin encoder-decoder model incorporating a neural ODE model. While I think that this approach is conceptually promising, I have major concerns about the validity of the estimated quantities.

The proposed approach considers two encoder models. The first (VAE) encoder model generates a latent representation z_i of data matrix x = (x_i)_i (cells x genes). The second encoder model generates a latent time representation that feeds with the latter latent representation into an ODE model (neural ode) generating latent states z_ti with initial condition z_t0 (latent state with lowest pseudotime). The decoder model decodes to x_i using both z_i and z_ti according to an objective that trades off the importance of z_i and z_ti according to a weight alpha. It is not clear according to which criteria the parameter alpha is chosen. What is the sensitivity of the estimated latent representation, pseudotime and velocity with respect to this choice. The author could for instance demonstrate the impact on the presented case studies by showing results for a spectrum of alpha settings.

What are the correlations of the velocities estimated with f_ODE and RNA velocity estimates? So far there is little assessment of the validity of the inferred velocities.
Fig. 5 shows the benchmark for a single dataset (for excitatory neuron development). This dataset is a rather simple dataset with a linear topology for which pseudotime estimation will produce a trajectory spanning the support of the dataset that happens to coincide with the ground truth. It would be good to extend this assessment to other datasets with more elaborate trajectory topology, at least for all other datasets considered in this study.

The proposed approach does infer velocities only from static transcriptional profiles (spliced mRNA) and does not do so independently by modeling of transcriptional dynamics by means of e.g. including information about unspliced RNA. I wonder whether the proposed approach is able to disambiguate the many different data consistent ways to come up with pseudotime estimates for datasets with more elaborate topologies.

The analysis of the dentate gyrus dataset constitutes a good showcase (Fig. 2). However it seems that the author have deliberately simplified the dataset by only considering a subset of differentiation branches, in particular omitting data for most of the branches, i.e. those leading to astrocytes, oligodendrocyte precursors (OPCs) and the pyramidal neurons of the five fields of the hippocampus: the subiculum, CA1, CA2, CA3 and hilus. Why did the authors make this choice? How do the velocity estimates look for the full dataset. I would not be surprised that the estimated root state for the full dataset would not coincide with ground truth root state(s) in the center of the data support if RNA velocity is estimated without accounting for independent information from e.g. unspliced mRNA levels.

The study of the pancreatic endocrinogenesis dataset (Fig. 3) is yet another good showcase for a dataset with	complex topology. It happens that the cells around the root states are in cell cycle and that proper velocity estimation/trajectory inference should indicate cycling behaviour/topology. As far as I can judge from the stream plots the inferred velocities do not indicate this, and rather point to a linear topology in the root state region. Here it would be good to compare the estimated velocities with those estimated by "conventional" RNA velocity estimates, such as scVelo run with the stochastic/dynamic model as well as other new velocity estimation approaches such as kappa-velo (Marot-Lassauzaie et al. 2022).

Further, is the approach able to detect the absence of velocity in datasets of exclusively terminally differentiated cells (e.g. PBMCs). As far as I understand the approach, I would expect it to still estimate varying pseudotime and velocity in such a situation. Could the author demonstrate this on a suitable dataset?

The authors claim that the proposed approach can correct for batch effects as indicated in coming up with a coherent representation of the neural activation dataset, in contrast to scVI without batch information (Fig 5d). Is it possible that the proposed approach overcorrects? For instance in the analysis of the pancreatic endocrinogenesis dataset (Fig. 3) where the author assesses the pseudotime/velocity estimates of held-out (Fev+) cells, the author shows only the latent representation generated for the full dataset (including the Fev+ cells). How does the latent representation of the model trained without these cells look like. Does this representation preserve the gap of the missing cell states or does it artifactually merge the remaining cell groups that in the full dataset are dissected by the Fev+ cells?


Minor concern:

Methods section: Does the encoder take as input x (as suggested in the methods section) or take a single cell expression profile as input?

The author claims demonstration of the approach on 17 datasets in the abstract. The main figures cover seven datasets only. Is this a typo in the abstract, or do I overlook additional datasets.

**Reviewer 2**

**Were you able to assess all statistics in the manuscript, including the appropriateness of statistical tests used?**

**Yes:** na

**Were you able to directly test the methods?**

Yes

**Comments to author:**

Summary
In this article, with the implementation of single-cell genomic data, the authors construct a deep learning framework named scTour based on variational autoencoder and neural ordinary differential equation to infer and predict cellular dynamics. The experimental results on 17 datasets indicate that scTour is a batch-insensitive tool that can infer and predict cellular dynamics robustly. However, despite the good performance of scTour in estimating the developmental pseudotime and reconstructing the underlying dynamics of unseen cellular states, this manuscript still has flaws in terms of the following specific points of view.

Major issues
1. The p values for Spearman correlation coefficients are not provided in Fig. 3.
2. Although Fig.3 shows scTour can successfully predict pseudotime of the unseen cellular state, the accuracy of predictions remains unclear. It would be better if the accuracy could be quantified.
3. Fig.1 and the description in the methods part are not matching. While there are two encoder networks fz and ft, receiving gene expression matrix as input, Fig.1 shows a shared encoder network for latent embeddings and times.
4. The models in the encoder network and decoder network need further clarification. If there are fully connected neural networks, they should be stated in the methods part.
5. More details should be given and described for the ODESolve function.
6. The number of layers and parameters of the autoencoder network needs to be clarified.
7. Methods of calculating the probability term in the loss function is unclear.
8. More details should be added in the model training part, such as experimental settings and parameter selection.
9. The ablation experiment for z, zt, and zlatent should be provided. It is unclear what is the contributions of z and zt for zlatent.
10. Detailed explanations should be provided regarding why 1/n is subtracted in line 556.

Minor issues
1. There are some grammar issues. For example, in line 429, it should be "predictions" instead of "prediction".
2. Please define KL divergence, NB-conditioned likelihood, and ZINB-conditioned likelihood and provide their original citations.
3. Please provide original citations for ELU and Adam optimizer.

**Authors Response**

**Point-by-point responses to the reviewers’ comments:**

**Reviewer 1 Comments:**

The author presents an innovative approach to perform pseudotime estimation and in turn use this estimate to estimate RNA velocity by means of an twin encoder-decoder model incorporating a neural ODE model. While I think that this approach is conceptually promising, I have major concerns about the validity of the estimated quantities.

**1. The proposed approach considers two encoder models. The first (VAE) encoder model generates a latent representation z_i of data matrix x = (x_i)_i (cells x genes). The second encoder model generates a latent time representation that feeds with the latter latent representation into an ODE model (neural ode) generating latent states z_ti with initial condition z_t0 (latent state with lowest pseudotime). The decoder model decodes to x_i using both z_i and z_ti according to an objective that trades off the importance of z_i and z_ti according to a weight alpha. It is not clear according to which criteria the parameter alpha is chosen. What is the sensitivity of the estimated latent representation, pseudotime and velocity with respect to this choice. The author could for instance demonstrate the impact on the presented case studies by showing results for a spectrum of alpha settings.**

I thank the reviewer for this comment. The default alpha (with a range from 0 to 1) is set to 0.5 which assigns equal importance to the reconstruction error from 𝑧 (VAE, weighted by 𝛼) and that from 𝑧t (neural ODE, weighted by 1 − 𝛼). Following the reviewer’s suggestion, additional models were trained with a spectrum of alpha values (from 0.1 to 0.9 with a step size of 0.1) for the two datasets shown in Fig. 2. The results were shown here (Figs. R1 and R2 below) and in Figs. S21 and S22.

For the first dataset which profiled the linear progression from nIPCs to mature granule cells, the estimated pseudotime, latent representation, and vector field were robust to the choice of alpha values (Fig. R1 below, Fig. S21). For the second dataset which profiled the bifurcation process from nIPCs to pyramidal neurons and granule cells, the estimated pseudotime was not sensitive to alpha values as well; but the latent representation and vector field were impacted by small alpha values (Fig. R2 below, Fig. S22). Specifically, the latent representation derived from small alpha (e.g., 0.1 to 0.3 given to reconstruction errors from 𝑧) tended to order the cells based on their pseudotime and thus cannot well separate cell types with similar developmental orders (e.g., immature pyramidal neurons and immature granule cells here). With larger alpha (alpha > 0.4, 0.5 by default), the separation between the two cell types was more evident. This was because latent *z* accounted for the transcriptomic structure of cells while latent *z*_t_ was more representative of the pseudotime ordering of cells; when a small weight (small alpha) is assigned to the reconstruction errors from *z*, this will result in underemphasized *z* due to insufficient training for this term. I have now added this analysis and made the point clear in the Methods section (lines 552-562).

**Fig. R1 (Fig. S21)** Assessment of the parameter alpha in the objective function in a dataset of granule cell differentiation. **a** UMAP visualizations of the latent representations (left, coloured by cell types), pseudotime (middle), and vector field (right) inferred from the scTour models trained with a series of alpha values (the alpha in the objective function). **b** The Spearman correlation coefficients calculated between the pseudotime estimated from the model with alpha set to 0.5 (the default setting) and those from the models with other alpha settings (left), the cosine similarities computed between the projected velocities from the model with the default alpha (0.5) and those from the other models (middle), and the cell type ASW measuring the biological signal conservation under the latent space inferred from different alpha settings (right). ASW, average silhouette width.

**Fig. R2 (Fig. S22)** Assessment of the parameter alpha in the objective function in a dataset of pyramidal neuron and granule cell development. **a** UMAP visualizations of the latent representations (left, coloured by cell types), pseudotime (middle), and vector field (right) inferred from the scTour models trained with a series of alpha values (the alpha in the objective function). **b** The Spearman correlation coefficients calculated between the pseudotime estimated from the model with alpha set to 0.5 (the default setting) and those from the models with other alpha settings (left), the cosine similarities computed between the projected velocities from the model with the default alpha (0.5) and those from the other models (middle), and the cell type ASW measuring the biological signal conservation under the latent space inferred from different alpha settings (right). ASW, average silhouette width.

**2. What are the correlations of the velocities estimated with f_ODE and RNA velocity estimates? So far there is little assessment of the validity of the inferred velocities. Fig. 5 shows the benchmark for a single dataset (for excitatory neuron development). This dataset is a rather simple dataset with a linear topology for which pseudotime estimation will produce a trajectory spanning the support of the dataset that happens to coincide with the ground truth. It would be good to extend this assessment to other datasets with more elaborate trajectory topology, at least for all other datasets considered in this study.**

I thank the reviewer for this comment. The velocities estimated from ***_fode_*** are at the latent representation level while RNA velocities are at the RNA level. To examine the correlation between them, I considered the velocity visualized under a certain embedding (UMAP here) which was the weighted combination of unitary displacement vectors, and based on this, calculated the cosine similarity between the vector from ***_fode_*** and that from the RNA velocity (scVelo’s stochastic model was used here given its better performance on the dataset mentioned below compared to the dynamical model). Following the reviewer’s suggestion, here I selected the dataset profiling mouse gastrulation and early organogenesis to assess the validity of the inferred velocities under a more elaborate trajectory topology. As shown in Fig. R3 below and in Fig. S17, compared to the RNA velocity estimated by scVelo, the velocity derived from ***_fode_*** not only delineated the main cell lineages of mesoderm, endoderm, and ectoderm, but also the sub-lineages. Specifically, ***_fode_*** captured the developmental trajectory of the erythroid lineage (from haemato-endothelial progenitors, blood progenitors to erythroid) as well as the starting cells (epiblast) of the entire process, which were all reversed in the RNA velocity estimates. The remaining cells (excluding the erythroid lineage and epiblast) showed a high cosine similarity between thevelocity estimates from *"#$* and scVelo. Further comparing the inferred pseudotime with that derived from the RNA velocity (velocity pseudotime from scVelo) and that from Palantir (with better performance than Monocle3 and Slingshot as shown in Fig. 5), scTour’s pseudotime displayed the highest correlation with the known developmental stages. Lastly, for the latent space generated, scTour showed comparable performance with scVI even when scVI took additional information (that is, batch factors) into consideration. In summary, scTour also showed good performance when applied to biological processes with more complex topologies, as now described in lines 320-332 of the manuscript.

**Fig. R3 (Fig. S17)** Benchmarking scTour against existing methods using the dataset profiling mouse gastrulation and early organogenesis. **a** UMAP displaying the cell types identified from the stage of mouse gastrulation and early organogenesis. The UMAP coordinates and cell type annotations are from the original study. PGC, primordial germ cells; NMP, neuromesodermal progenitors; Def., definitive; ExE, extra-embryonic. **b** UMAP visualizations of the vector field inferred from scTour (left) and RNA velocity estimated by scVelo (right). Dotted circles mark the epiblast cells and cells from the erythroid lineage. **c** UMAP visualization of the cosine similarities calculated between the projected velocity from scTour and that from scVelo. **d** UMAP displaying the pseudotime estimated by scTour (left), scVelo (middle), and Palantir (right). **e** The Spearman correlation coefficients calculated between the pseudotime from the three methods shown in **d** and known developmental stages. **f** UMAP visualizations based on the latent space derived from scTour (left), scVI with no batch information provided (middle), and scVI with batch information incorporated (right). **g** Metrics quantifying the degree of batch correction (batch mixing entropy and graph connectivity) and biological signal conservation (cell type ASW) for each method. ASW, average silhouette width.

**3. The proposed approach does infer velocities only from static transcriptional profiles (spliced mRNA) and does not do so independently by modeling of transcriptional dynamics by means of e.g. including information about unspliced RNA. I wonder whether the proposed approach is able to disambiguate the many different data consistent ways to come up with pseudotime estimates for datasets with more elaborate topologies.**

I thank the reviewer for this point. As shown above for the dataset of mouse gastrulation and early organogenesis, as well as shown in Fig. S4 (Fig. R4 below) for a dataset of cardiogenesis captured in gastruloids and in Fig. S10 (Fig. R5 below) for another dataset profiling haematopoiesis, the estimated pseudotime and velocity from scTour correctly captured their underlying trajectories.

**Fig. R4 (Fig. S4a-c)** scTour captures the developmental cellular dynamics in embryonic organoids. **a** UMAP visualization of the cell types from 30,496 cells collected from mouse gastruloids. The UMAP coordinates and cell type annotations are drawn from the original study. NMPs, neuromesodermal progenitors. **b-c** UMAP visualizations of the inferred developmental pseudotime (**b**) and transcriptomic vector field (**c**) from the scTour model trained using 60% of all cells.

**Fig. R5 (Fig. S10a-c)** scTour captures the developmental cellular dynamics during hematopoiesis. **a** UMAP visualization of the cell lineages from 1,947 cells profiled by scNT-seq during hematopoiesis. The UMAP coordinates and cell type annotations are from the original study. HSC, hematopoietic stem cell; Meg, megakaryocyte; Ery, erythrocyte; Bas, basophil; Mon, monocyte; Neu, neutrophil; GMP-like, granulocyte and monocyte progenitor-like cells; MEP-like, Meg and Ery progenitor-like cells. **b-c** UMAP visualizations of the inferred developmental pseudotime (**b**) and transcriptomic vector field (**c**) from the scTour model trained using 90% of all cells.

**4. The analysis of the dentate gyrus dataset constitutes a good showcase (Fig. 2). However it seems that the author have deliberately simplified the dataset by only considering a subset of differentiation branches, in particular omitting data for most of the branches, i.e. those leading to astrocytes, oligodendrocyte precursors (OPCs) and the pyramidal neurons of the five fields of the hippocampus: the subiculum, CA1, CA2, CA3 and hilus. Why did the authors make this choice? How do the velocity estimates look for the full dataset. I would not be surprised that the estimated root state for the full dataset would not coincide with ground truth root state(s) in the center of the data support if RNA velocity is estimated without accounting for independent information from e.g. unspliced mRNA levels.**

I thank the reviewer for this question. This dataset was simplified as a demonstration of a bifurcation process. Following the reviewer’s suggestion, I have now incorporated all the cell types as described in the RNA velocity paper (Manno et al. 2018, Nature). Again, the inferred pseudotime, velocity and latent representation recapitulated the process from radial glia, nIPC, to neuroblasts, branching out further into either granule cells or pyramidal neurons, with the latter from the five fields of the hippocampus also captured (Fig. R6 here, Fig. S2). The trend of differentiation from pre-OPCs to OPCs was also reflected. There was a directional flow from astrocytes to pre-OPC, indicating the possible glioblast state of these astrocytes as they expressed the glioblast marker *Tnc* and OPC marker *Olig1*, and *Olig1* was previously shown to be expressed in intermediate glial progenitor cells with possible bipotential to astrocytes and oligodendrocytes (Weng et al. 2019, Cell Stem Cell).

However, as the reviewer mentioned, the root state for this dataset was not defined unambiguously, as the astrocytes showed similar or a bit lower pseudotime compared with the radial glia. This is probably due to the shared glia-like traits of radial glia and glioblasts (or immature astrocytes here) that blur their transcriptomic distinctions and thus pseudotime ordering without the intervention from other complementary information such as unspliced RNAs. Nonetheless, when applied to another independent dataset profiling this similar process from radial glia to either glial or neuronal lineages (part of the dataset of the developing mouse brain atlas from Manno et al. 2021 Nature, Fig. R7 below), the estimated pseudotime and velocity correctly delineated the developmental trajectory from radial glia to either glioblasts which further developed into astrocytes and OPCs, or to the neuronal lineage. The improved performance for this dataset was possibly due to a clearer developmental order between the radial glia and glioblast as the latter was detected much later (after about E14).

In summary, without additional information from unspliced RNAs, scTour still has the capacity to resolve the branching events particularly when temporal information is imprinted in the transcriptomic properties of cells. I have now added the results from the full dataset in the Results section (lines 147-151).

**Fig. R6 (Fig. S2)** Application of scTour to the developing mouse hippocampus dataset. **a** *t*-SNE plot of scTour’s vector field with colours indicating the cell types. OPC, oligodendrocyte precursors. **b** *t*-SNE plot of the expression of glioblast marker *Tnc* (top) and OPC marker *Olig1* (bottom). Red dotted circle marks the astrocytes. **c** *t*-SNE plot of the pseudotime estimated by scTour. **d** UMAP visualization of the latent space derived from scTour with colours denoting the cell types as in **a**.

**Fig. R7** Application of scTour to the developing mouse brain dataset. **a** *t*-SNE plot showing one neurogenic and the gliogenic lineages from the developing mouse brain dataset (bottom inset). The *t*-SNE coordinates from the original study are used here. **b** *t*-SNE plot visualizing scTour’s vector field with colours indicating the cell types as shown in **a**. **c** *t*-SNE plot of the pseudotime estimated by scTour. **d** UMAP visualization of the latent space derived from scTour with colours representing the cell types as in **a**.

**5. The study of the pancreatic endocrinogenesis dataset (Fig. 3) is yet another good showcase for a dataset with complex topology. It happens that the cells around the root states are in cell cycle and that proper velocity estimation/trajectory inference should indicate cycling behaviour/topology. As far as I can judge from the stream plots the inferred velocities do not indicate this, and rather point to a linear topology in the root state region. Here it would be good to compare the estimated velocities with those estimated by "conventional" RNA velocity estimates, such as scVelo run with the stochastic/dynamic model as well as other new velocity estimation approaches such as kappa-velo (Marot-Lassauzaie et al. 2022).**

I thank the reviewer for this comment. Following the reviewer’s suggestion, I compared the velocity from scTour with that estimated from the stochastic and dynamical models of scVelo and that from kappa-velo. As shown in Fig. S13 (Fig. R8 below) and now described in lines 197-204 of the manuscript, scTour and scVelo were able to delineate the entire differentiation course of endocrinogenesis while kappa-velo only illustrated a partial view of this process when no prior knowledge was provided. Focusing on the cycling cells, the two models of scVelo captured both the S to G2M transition and the exit of the cell cycle (highlighted in blue and red dotted circles in Fig. R8). For scTour, it captured the partial S-G2M transition and full cell cycle exit (red dotted circle in Fig. R8). Neither of these two processes were properly delineated by kappa-velo.

To further test the ability of scTour’s vector field to depict cycling processes, I used another dataset profiling the developing mouse retina (Fig. S19, Fig. R9 below). Similar with the RNA velocity estimated by scVelo, the velocity from scTour clearly characterized the three branching lineages (PR, AC/HC, and RGC). In this process, scTour captured the S-G2M transition but failed to reflect the subsequent transition from M to G1/S. Further examination of the pseudotime inferred by scTour showed that cells from the S phase displayed lower pseudotime than those from the G2M phase. Given the fact that the vector field from scTour (the derivative of the latent states with respect to time) followed the direction of time, it thus cannot point cells with higher pseudotime to those with lower pseudotime, that is, it cannot cycle from G2M (higher pseudotime) back to S (lower pseudotime) phase. I have now discussed this point in Discussion (lines 430-434). Despite this limitation, scTour’s vector field can be advantageous as well. For instance, it can be applied to scATAC-seq data as it only relies on the cell by gene/peak abundance matrix. When applying scTour to the same process of retinal development but profiled by scATAC-seq, scTour also captured the underlying dynamics by the inferred pseudotime and velocities (Fig. S18, Fig. R10 below).

**Fig. R8 (Fig. S13)** Comparisons of scTour’s vector field with velocities from other methods. **a** UMAP visualizations of scTour’s vector field (left), the RNA velocity estimated from scVelo’s stochastic and dynamical models (middle), the velocity derived from κ-velo (the fourth panel), and the cell cycle scores (right) based on the dataset of pancreatic endocrinogenesis. **b** Zoom-in view of the velocity estimates of the cycling cells. Blue and red dotted circles highlight the S-G2M transition and exit of the cell cycle, respectively.

**Fig. R9 (Fig. S19)** Delineation of the cycling process by scTour’s vector field and RNA velocity. **a** UMAP visualizations of the cell types from the developing mouse retina (top) and the cell cycle scores (bottom). PR, photoreceptors; AC/HC, amacrine/horizontal cells; RGC, retinal ganglion cells. **b** UMAP visualizations of the vector field and pseudotime estimated by scTour. Dotted circle highlights the cycling cells. **c** As in **b**, but with the velocity and pseudotime estimated by scVelo.

**Fig. R10 (Fig. S18)** Application of scTour to scATAC-seq data. **a** Latent semantic indexing (LSI)-based UMAP visualization of the cell types identified from 4,883 cells of the human fetal retina measured by scATAC-seq. RGC, retinal ganglion cells; CON, cones; Npre, neurogenic precursors; RGCpre, RGC precursors; H/Apre, the horizontal/amacrine precursors; MPC, multipotent progenitor cells; HOR, horizontal cells. **b-d** UMAP visualizations of the inferred developmental pseudotime (**b**), epigenetic vector field (**c**), and latent space (**d**) from the scTour model trained using 90% of cells and top 25% of peaks (34,670 genomic regions).

**6. Further, is the approach able to detect the absence of velocity in datasets of exclusively terminally differentiated cells (e.g. PBMCs). As far as I understand the approach, I would expect it to still estimate varying pseudotime and velocity in such a situation. Could the author demonstrate this on a suitable dataset?**

I thank the reviewer for this point. Following the reviewer’s suggestion, I applied scTour to one PBMC dataset and an additional dataset profiling the cellular states in the human decidua. Cell types from these two datasets were terminally differentiated cells, and no velocities were expected. However, scTour still detected dynamics across cell types with varying pseudotime and velocity observed as the reviewer expected (Fig. S20, Fig. R11 below). The varying pseudotime was caused by the distinct transcriptomes in different cell types as the pseudotime was encoded from the transcriptome of each cell. This further resulted in velocities since scTour’s vector field was the derivative of the latent states with respect to time. However, scTour should detect no dynamics when applied to one terminal state exclusively. Indeed, when training a model based only on one terminal state (e.g., B cells), scTour assigned similar pseudotime to these cells and no obvious velocities were detected (Fig. S20, Fig. R11 below).

I further tested whether modelling the transcriptional kinetics using RNA velocity can avoid these erroneous dynamics across terminal cell types. Similar with scTour, RNA velocity (from scVelo) also displayed dynamics for these two datasets, indicating a possible common issue when detecting the absence of velocity across terminal cell types (Fig. S20, Fig. R11 below). I have now discussed this point in Discussion (lines 434-440).

**Fig. R11 (Fig. S20)** Application of scTour to terminally differentiated cells. **a, b** UMAP visualizations of the transcriptomic vector field and developmental pseudotime inferred from the scTour model trained based on the PBMC 3k dataset (**a**, from 10x Genomics) and human decidual dataset (**b**, Vento-Tormo et al. 2018, Nature). dS, decidual stromal cells; dNK, decidual natural killer cells; dM, decidual macrophages. **c, d** UMAP visualizations of the pseudotime and vector field derived from the scTour model trained based only on CD14 monocytes (**c**, top), B cells (**c**, bottom), and dS1 (**d**, top) and dNK2 (**d**, bottom). **e, f** UMAP visualizations of the RNA velocity and velocity pseudotime estimated by scVelo for the PBMC 3k (**e**) and human decidual (**f**) datasets.

**7. The authors claim that the proposed approach can correct for batch effects as indicated in coming up with a coherent representation of the neural activation dataset, in contrast to scVI without batch information (Fig 5d). Is it possible that the proposed approach overcorrects? For instance in the analysis of the pancreatic endocrinogenesis dataset (Fig. 3) where the author assesses the pseudotime/velocity estimates of held-out (Fev+) cells, the author shows only the latent representation generated for the full dataset (including the Fev+ cells). How does the latent representation of the model trained without these cells look like. Does this representation preserve the gap of the missing cell states or does it artifactually merge the remaining cell groups that in the full dataset are dissected by the Fev+ cells?**

I thank the reviewer for this point. Based on the pancreatic endocrinogenesis dataset, the latent representation from the model trained by excluding the intermediate *Fev*+ cells preserved the expected gap between endocrine progenitors and the terminal states (lines 218-221 of the manuscript; Fig. S14, Fig. R12 below). Similar results were obtained when excluding other cellular states along this process (Fig. S14, Fig. R12 below), demonstrating the ability of scTour’s latent space in reflecting the true topologies without overcorrection.

**Fig. R12 (Fig. S14e)** UMAP visualizations of the latent representation derived from the scTour model trained with a certain cellular state held out. EP, endocrine progenitor.

**Minor concern:**

**1.Methods section: Does the encoder take as input x (as suggested in the methods section) or take a single cell expression profile as input?**

The encoder and decoder networks consider each cell independently (a single cell expression profile), but the neural ODE needs to consider all the cells (cells within a mini-batch here) by taking the pseudotime of the cells and the latent at the initial time point as input. I have now made this clear in the Methods section (line 467).

**2. The author claims demonstration of the approach on 17 datasets in the abstract. The main figures cover seven datasets only. Is this a typo in the abstract, or do I overlook additional datasets.**

There are 17 datasets (19 datasets in the revised version after adding the mouse gastrulation and developing mouse retina datasets) demonstrated in the manuscript, with eight of them shown in the main figures and the remaining ones in the supplemental figures (lines 166­181, 320-332 of the manuscript and Figs. S4-11, S17-19).

**Reviewer 2 Comments:**

In this article, with the implementation of single-cell genomic data, the authors construct a deep learning framework named scTour based on variational autoencoder and neural ordinary differential equation to infer and predict cellular dynamics. The experimental results on 17 datasets indicate that scTour is a batch-insensitive tool that can infer and predict cellular dynamics robustly. However, despite the good performance of scTour in estimating the developmental pseudotime and reconstructing the underlying dynamics of unseen cellular states, this manuscript still has flaws in terms of the following specific points of view.

**Major issues:**

**The p values for Spearman correlation coefficients are not provided in Fig. 3.**

I thank the reviewer for pointing out this. I have now added the *p* values (*p* < 1e-5) in Fig 3.

**Although Fig.3 shows scTour can successfully predict pseudotime of the unseen cellular state, the accuracy of predictions remains unclear. It would be better if the accuracy could be quantified.**

I thank the reviewer for this comment. I have now quantified the prediction accuracy by charting the receiver operating characteristic (ROC) curve based on the predicted pseudotime of the held-out cell type. Specifically, I tested whether the predicted pseudotime here for *Fev*+ cells placed those cells correctly along the differentiation path (*Fev*+ cells were intermediate cells between endocrine progenitors and terminal states). I thus computed the AUC for the binary classifications of progenitors versus *Fev*+ cells, as well as *Fev*+ versus terminal cells. This calculation was based on the predicted pseudotime for *Fev*+ cells and inferred pseudotime for the other cellular states from the model trained through excluding *Fev*+ cells. This analysis showed that both classifications yielded high AUC scores (0.996 and 0.938), demonstrating the accuracy of the predicted pseudotime by scTour. I have now added this result to Fig. 3 (Fig. R13 below).

**Fig. R13** (bottom panel in **Fig. 3c**) ROC curve for the binary classifications of endocrine progenitors versus *Fev*+ cells (green) and *Fev*+ versus terminal cells (blue) based on the predicted pseudotime for *Fev*+ cells and inferred pseudotime for the other cellular states. Area under the ROC curve (AUC) is shown in the plot.

**Fig.1 and the description in the methods part are not matching. While there are two encoder networks fz and ft, receiving gene expression matrix as input, Fig.1 shows a shared encoder network for latent embeddings and times.**

I thank the reviewer for this point and sorry for the confusion. There are two layers (excluding the input layer) for each encoder network 𝑓z and 𝑓t, with the first hidden layer (128 hidden units by default) shared between 𝑓z and 𝑓t. I have now made this clear in the Methods section (lines 477-478) to match Fig. 1, and also have provided a supplemental table (Table S1, shown here below question 4) to show the detailed structure of scTour.

**The models in the encoder network and decoder network need further clarification. If there are fully connected neural networks, they should be stated in the methods part.**

I thank the reviewer for pointing out this. The layers in the encoder and decoder networks are fully connected neural networks. I have now provided further clarifications in the Methods section (lines 467-468, 477, 483, 497-498) and a supplemental table (Table S1) for the neural networks used in scTour.

**Table S1**. Neural networks used in scTour.

**More details should be given and described for the ODESolve function.**

I thank the reviewer for this point. I have now added more details including the formula and description for the ODESolve function in lines 487-495 of the manuscript.

**The number of layers and parameters of the autoencoder network needs to be clarified.**

I thank the reviewer for this comment. I have now added a supplemental table (Table S1) to provide detailed information for the entire structure of the neural networks involved. I have also expanded the descriptions regarding the neural networks used in the Methods section (lines 467-468, 477, 483, 497-498).

**Table S1**. Neural networks used in scTour.

**Methods of calculating the probability term in the loss function is unclear.**

I thank the reviewer for pointing out this. There are three modes for calculating the probabilities (i.e., the reconstruction errors from latent z and 𝑧t) in scTour. I have now added the formulae and descriptions for the probability term under each mode (MSE, lines 509-514; NB, lines 515-527; ZINB, lines 528-535).

**More details should be added in the model training part, such as experimental settings and parameter selection.**

I thank the reviewer for this comment. I have now added more details in the model training part, including the requirements for data input under different reconstruction modes (lines 540-544), the methods for solving ODE (lines 551-552), and hyperparameter selections (lines 552-566). I have also provided a table summarizing the default parameters for model training (Table S2).

**Table S2**. Hyperparameters in scTour model training.

| **hyperparameter** | **default value** |
| --- | --- |
| # hidden units in layer 1 of encoder | 128 |
| latent dimension | 5 |
| # hidden units in layer 1 of neural ODE | 25 |
| # hidden units in layer 1 of decoder | 128 |
| mini-batch size | 1024 |
| optimizer | Adam |
| learning rate | 0.001 |
| weight decay | 1.00E-06 |
| *!* in the Adam optimizer | 0.01 |
| alpha for reconstruction errors from z | 0.5 |
| # epoch | min(400, 10000 / #cells * 400) |
| ODE solver | Euler |
| reconstruction mode | nb |
| percentage of cells used for training | 0.9 (if #cells ≤ 10000) or 0.2 (if #cells > 10000) |

**The ablation experiment for z, zt, and zlatent should be provided. It is unclear what is the contributions of z and zt for zlatent.**

I thank the reviewer for this point. z*_iatent_* is the weighted combination of (𝜔) and z*_t_* (1 − 𝜔). To assess the contributions of z and z*_t_* to z*_iatent_*_,_ I tested a range of 𝜔 values (from 0 to 1 with a step size of 0.1) based on four datasets: granule cell development in the mouse dentate gyrus (linear process, Fig. 2a), excitatory neuron development in the human cortex (linear process, Fig. 4a), granule cell and pyramidal neuron development in the mouse hippocampus (bifurcation process, Fig. 2b), and endocrinogenesis in the mouse pancreas (multifurcation process, Fig. 3a). As shown in Figs. S23-26 (two out of the four datasets are shown in Fig. R14 and Fig. R15 below as a demonstration here), z*_t_* derived from the neural ODE captures the developmental order of the cells as reflected in the UMAP as atemporal spectrum; while *z* derived from the encoder depicts the global transcriptomic structure which tends to place cells from the same cell type together as a cluster in the UMAP. The weighted combination of the two (the default value of 𝜔 is 0.5) can chart finer cell trajectories by considering both aspects.

**Fig. R14 (Fig. S24)** Assessment of contributions of *z* and *z*_t_ to scTour’s latent representation in a dataset of excitatory neuron development. **a** UMAP visualizations of the latent representations derived from varying combinations of *z* (from the variational inference) and *z*_t_ (from the ODE solver) inferred based on the dataset of excitatory neuron development in the human cortex. Colours of cells indicate cell types. cyc. prog., cycling progenitors; nIPCs, neuronal intermediate progenitor cells; ExNs, excitatory neurons. **b** cell type average silhouette width (ASW) quantifying the biological signal conservation under different latent representations shown in **a**.

**Fig. R15 (Fig. S25)** Assessment of contributions of *z* and *z_t_* to scTour’s latent representation in a dataset of granule cell and pyramidal neuron development. **a** UMAP visualizations of the latent representations derived from varying combinations of *z* (from the variational inference) and *z_t_* (from the ODE solver) inferred based on the dataset of granule cell and pyramidal neuron development in the mouse hippocampus. Colours of cells indicate cell types. **b** cell type average silhouette width (ASW) quantifying the biological signal conservation under different latent representations shown in **a**.

**10. Detailed explanations should be provided regarding why 1/n is subtracted in line 556.**

I thank the reviewer for this comment. This operation is used to control for the non-uniform density of the data points under an embedding. I have now added the explanation for this in lines 633-634 of the Methods section.

**Minor issues:**

**1.There are some grammar issues. For example, in line 429, it should be "predictions" instead of "prediction".**

I thank the reviewer for pointing out the grammar errors. I have now carefully checked the grammars and corrected the errors throughout the manuscript.

**Please define KL divergence, NB-conditioned likelihood, and ZINB-conditioned likelihood and provide their original citations.**

I thank the reviewer for this comment. I have now added in the Methods section the definitions for KL divergence (lines 503-506), NB-conditioned likelihood (lines 515-527), and ZINB-conditioned likelihood (lines 528-535), and provided the corresponding citations (lines 504, 508-509).

**Please provide original citations for ELU and Adam optimizer.**

I thank the reviewer for pointing out these missing citations. I have now added the original citations for ELU (line 537) and Adam optimizer (line 562) in the manuscript.

**Second round of review**

**Reviewer 1**

The author has addressed most of my comments. However, a few concerns remain. Please see below.

The author assessed the correlation of the velocities estimated with f_ODE and RNA velocity estimates for a specific admittedly complex dataset profiling mouse gastrulation and early organogenesis. It is reassuring to see that there mostly is good agreement with the velocity estimates with scvelo based velocity estimates. However, and as noted by the author RNA velocity does not suggest coherent transitions across the different erythroid types. It would be helpful to see how this comparison plays out for all other datasets considered in this study.

Further, it seems that the scTour model by design will favor fitting a neural ODE model that "pushes" until the boundaries of the populated expression space and along the way suggest coherent velocities. This behaviour would preclude identifying regions of expression space where a developmental process is happening in a less coherent fashion, as it might be the case - as the RNA velocity analysis suggests - for the sequence of erythroid cell states. Can the author show examples where scTour is able to identify regions with less coherent velocity?

The analysis of terminally differentiated cells, such as in the PBMC dataset indicates that scTour as well as scvelo report non-zero velocities, e.g. for the the different NK/T-cell subtypes. Stream plots tend to suggest coherent velocity where the individual velocities exhibit high local entropy/variance. What is the local entropy/variance of the velocities reported by scTour/scvelo when applied to the whole PBMC dataset? The analysis of the separate cell type clusters seems to result in less coherent velocity? What was the choice of the start state for the model fit? Are the resulting velocity estimates sensitive to the choice of the start state?

The analysis of the pancreatic endocrinogenesis dataset with respect to the resolution of the cell cycle topology demonstrates that scTour cannot identify the cyclic arrangement of velocities. This is an inherent limitation by the definition of the scTour model and is now discussed in the Discussion section.

The reanalysis of the full developing mouse hippocampus dataset also shows that in the absence of independent estimates of velocity, wrong root states are chosen and consequently leading to an erroneous ordering of cells along some branches of the developmental process. The author had to add another example application dataset (developing mouse brain atlas) to demonstrate that there are cases where this unbiased analysis can deliver the right ordering along complex developmental hierarchies. How can a user in an exploratory setting decide whether one can trust the ordering of scTour?

**Reviewer 2**

The authors have addressed all my comments for this paper and answered the technical questions I have about the method. The paper has been significantly improved after revision. I recommend the editor accept this paper and publish it.

**Authors Response**

**Point-by-point responses to the reviewers’ comments:**

**The author assessed the correlation of the velocities estimated with f_ODE and RNA velocity estimates for a specific admittedly complex dataset profiling mouse gastrulation and early organogenesis. It is reassuring to see that there mostly is good agreement with the velocity estimates with scvelo based velocity estimates. However, and as noted by the author RNA velocity does not suggest coherent transitions across the different erythroid types. It would be helpful to see how this comparison plays out for all other datasets considered in this study.**

Firstly, I would like to point out that RNA velocity produced erroneous ordering (reversed order) for the different erythroid types while scTour correctly delineated this lineage (see Fig. S17b, lines 324-328 in the manuscript). Secondly, most public scRNA-seq datasets do not provide spliced and unspliced count matrices which are needed for RNA velocity analysis. Deriving such matrices from FASTQ files (including downloading those files) for all the 19 datasets used in this study is, in practice, not realistic given the cost of time and effort. Moreover, although these datasets were not directly compared with RNA velocity (which is not the only focus of this study as velocity analysis is only one of the several functionalities in scTour; other functionalities include pseudotime ordering, latent representations, and prediction of cellular dynamics), they showed expected vector fields that are in line with the ground truth by using scTour.

Nevertheless, I performed such comparisons for three additional datasets with spliced and unspliced information available (in addition to the mouse gastrulation and organogenesis dataset): 1) endocrinogenesis in mouse pancreas; 2) excitatory neuron development in human cortex; 3) hematopoiesis. As shown in the figure below, all three datasets showed a good agreement between scTour’s vector field and RNA velocity. Notably, for the last dataset profiling hematopoiesis, scVelo cannot delineate this process correctly; instead, deriving proper RNA velocity has to rely on Dynamo on the basis of new/total RNA from metabolic labelling (Qiu et al., Cell, 2022). Thus, scTour was compared with Dynamo for this dataset, and the result showed that scTour’s vector field displayed high concordance with that from Dynamo.

**Fig. R1** UMAP visualizations of the cosine similarities calculated between the projected velocity from scTour and that from scVelo/Dynamo for the datasets profiling endocrinogenesis in mouse pancreas (**a**), excitatory neuron development in human cortex (**b**), and hematopoiesis (**c**).

**Further, it seems that the scTour model by design will favor fitting a neural ODE model that "pushes" until the boundaries of the populated expression space and along the way suggest coherent velocities. This behaviour would preclude identifying regions of expression space where a developmental process is happening in a less coherent fashion, as it might be the case - as the RNA velocity analysis suggests - for the sequence of erythroid cell states. Can the author show examples where scTour is able to identify regions with less coherent velocity?**

Firstly, I would like to reiterate that for the erythroid example mentioned by the reviewer, scVelo-based RNA velocity failed to delineate the differentiation course proposed by the original study (Pijuan-Sala et al., 2019, Nature). scVelo gave the wrong order (reversed order) for this erythroid lineage while scTour correctly depicted this lineage (see Fig. S17b).

Secondly, regarding the ability of scTour to identify regions with less coherent velocity, the example shown in Fig. 3 already demonstrates that scTour can correctly depict a discontinuous process where the intermediate cellular state (*Fev*+) was excluded from the endocrinogenesis process. In this example, the pseudotime (Fig. 3b), vector field (Fig. 3d), and latent representations (Fig. S14e) inferred by scTour accurately reflected this gap, that is, a non-coherent region. The results confirm the ability of scTour to capture such regions.

**The analysis of terminally differentiated cells, such as in the PBMC dataset indicates that scTour as well as scvelo report non-zero velocities, e.g. for the different NK/T-cell subtypes. Stream plots tend to suggest coherent velocity where the individual velocities exhibit high local entropy/variance. What is the local entropy/variance of the velocities reported by scTour/scvelo when applied to the whole PBMC dataset? The analysis of the separate cell type clusters seems to result in less coherent velocity? What was the choice of the start state for the model fit? Are the resulting velocity estimates sensitive to the choice of the start state?**

When performing velocity analysis for the whole PBMC dataset using scTour or scVelo, the distribution of local variance (defined here as 1 - cosine similarity averaged across the 30 nearest neighbours for each cell) is arbitrary (see Fig. R2 below). For example, the B, CD4 T, and CD14+ monocytes exhibited high local variance based on scTour’s velocity. Regarding the velocity of scVelo, it has high local variance in cell types such as CD8 T and NK cells. Thus, performing a velocity analysis, using either scTour or scVelo, is trivial in terms of deriving biological relevance in this PBMC case, as both tools lead to misinterpretation. The analysis of the separate cell type clusters (CD14+ monocytes as an example here) resulted in less coherent velocity (higher local variance, Fig. R2b below) by using scTour, consistent with the observation reported in the round-one rebuttal letter. In general, applying velocity analysis to mature cell types and interrogating its resulting details are possibly trivial in practical applications.

As with scVelo, scTour does not need the users to specify the start state, and therefore the resulting velocity estimates are not sensitive (more precisely, not related) to the choice of the start state. This has been mentioned in the original manuscript (e.g., lines 49 and 300).

**Fig. R2** Local variance for velocities estimated by scTour and scVelo for the PBMC dataset. **a** UMAP visualizations of the cell types in the PBMC dataset (left), the local variance of the velocities derived from scTour (middle) and scVelo (right). The local variance was defined as 1 - cosine similarity averaged across the 30 nearest neighbours for each cell based on the projected velocity. **b** The local variance of the velocities for CD14+ monocytes derived from the analysis of the whole PBMC dataset (blue) and the analysis of only CD14+ monocytes (orange).

**The analysis of the pancreatic endocrinogenesis dataset with respect to the resolution of the cell cycle topology demonstrates that scTour cannot identify the cyclic arrangement of velocities. This is an inherent limitation by the definition of the scTour model and is now discussed in the Discussion section.**

**The reanalysis of the full developing mouse hippocampus dataset also shows that in the absence of independent estimates of velocity, wrong root states are chosen and consequently leading to an erroneous ordering of cells along some branches of the developmental process. The author had to add another example application dataset (developing mouse brain atlas) to demonstrate that there are cases where this unbiased analysis can deliver the right ordering along complex developmental hierarchies. How can a user in an exploratory setting decide whether one can trust the ordering of scTour?**

I think this is a common question for all trajectory inference methods. As emphasised in a study benchmarking different trajectory inference methods (Saelens et al.*,* Nature Biotechnology, 2019), “there is no ‘one-size-fits-all’ method that works well on every dataset”.

For instance, back to the example of erythroid cell ordering in the dataset profiling mouse gastrulation and organogenesis, scVelo fails to chart its trajectory. This raises a similar question: whether the users can trust the RNA velocity and the ordering from scVelo in an exploratory setting? Thus, it is usually not possible to find a tool whose results can be fully trusted without any post-inference inspection. scTour, as with other methods such as scVelo, needs post-inference examination and inspection based on knowledge/observations from other kinds of analyses.

Actually, even without including information from both spliced and unspliced mRNAs, the ability of scTour to infer the underlying dynamics (pseudotime, vector field, latent representations) of diverse processes including neurogenesis, endocrinogenesis, gastrulation and early organogenesis, skeletal muscle development, hematopoiesis, and reprogramming has been demonstrated in 19 datasets used in this study.

**Third round of review**

**Reviewer 1**

The author addressed all comments to my satisfaction.

One minor issue remains: it would be pertinent to add to the discussion section a further discussion of the limitation of identifying root states and correct pseudotime orderings according to the author's demonstration in the application of scTour to the developing mouse hippocampus dataset (now Fig. R6 (Fig. S2)). A fully differentiated cell type (instead a stem like cell type) was erroneously assigned root state status with lowest pseudotime.
